# Substitution effect and effect of axle’s flexibility at (pseudo-)rotaxanes

**DOI:** 10.3762/bjoc.10.131

**Published:** 2014-06-05

**Authors:** Friedrich Malberg, Jan Gerit Brandenburg, Werner Reckien, Oldamur Hollóczki, Stefan Grimme, Barbara Kirchner

**Affiliations:** 1 Mulliken Center for Theoretical Chemistry, Rheinische Friedrich-Wilhelms-Universität Bonn, Beringstr. 4, 53115 Bonn, Germany

**Keywords:** dispersion interaction, hydrogen bond, supramolecular chemistry, template, theoretical chemistry

## Abstract

This study investigates the effect of substitution with different functional groups and of molecular flexibility by changing within the axle from a single C–C bond to a double C=C bond. Therefore, we present static quantum chemical calculations at the dispersion-corrected density functional level (DFT-D3) for several Leigh-type rotaxanes. The calculated crystal structure is in close agreement with the experimental X-ray data. Compared to a stiffer axle, a more flexible one results in a stronger binding by 1–3 kcal/mol. Alterations of the binding energy in the range of 5 kcal/mol could be achieved by substitution with different functional groups. The hydrogen bond geometry between the isophtalic unit and the carbonyl oxygen atoms of the axle exhibited distances in the range of 2.1 to 2.4 Å for six contact points, which shows that not solely but to a large amount the circumstances in the investigated rotaxanes are governed by hydrogen bonding. Moreover, the complex with the more flexible axle is usually more unsymmetrical than the one with the stiff axle. The opposite is observed for the experimentally investigated axle with the four phenyl stoppers. Furthermore, we considered an implicit continuum solvation model and found that the complex binding is weakened by approximately 10 kcal/mol, and hydrogen bonds are slightly shortened (by up to 0.2 Å).

## Introduction

**Figure 1:**
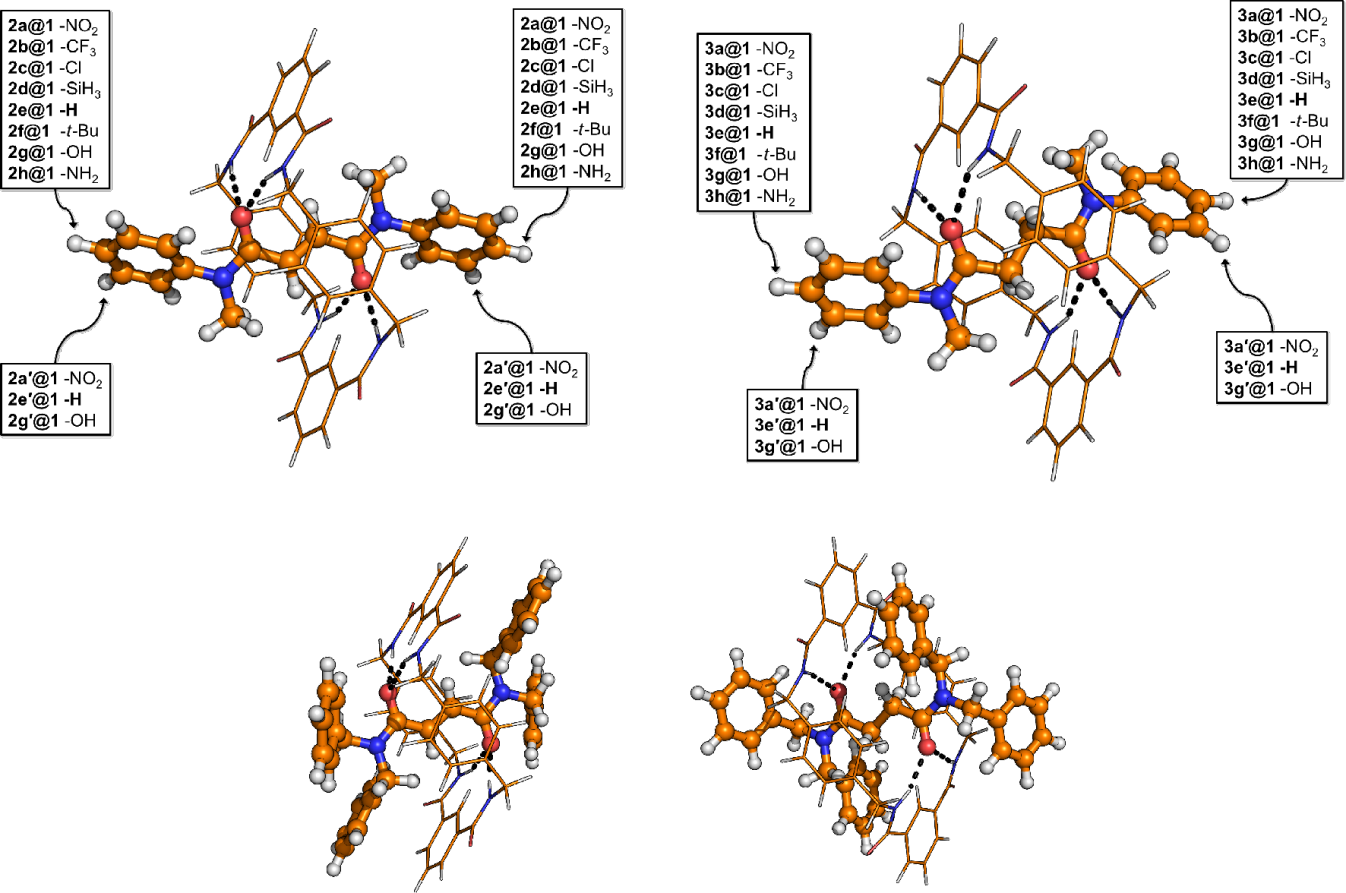
Chemical structure of the investigated systems. Left: Double bond within the axle; Right: Single bond within the axle. Red marks oxygen atoms, blue nitrogen atoms, orange carbon atom and hydrogen atoms are given in white. The labeling according to the substitution is given in bold letters. Structures below will be denoted **4@1** (left) and **5@1** (right).

Rotaxanes are prototypes for molecular machines and molecular switches [1–3]. They are mechanically interlocked molecules consisting of a macrocycle, called “wheel”, threaded on a linear chain, termed “axle”, see Figure 1 for examples. Typically, the axle has at least one recognition site – often hydrogen bond donors or acceptors [4–5] – for the wheel, because most rotaxanes are obtained from template synthesis [6–7]. Bulky stopper groups at the ends of the axle prevent the wheels from dethreading. Rotaxanes without their stopper groups are often referred to as pseudorotaxanes. It is implicitly assumed that these stopper functionalities have no further influence on the electronic structure of the axle, hence neither on the axle–wheel interaction.

Applications of rotaxanes are many-fold, for example there is an interest in understanding the motions carried out by both entities with respect to each other. This can lead to molecular machines via pirouetting [8], or molecular shuttles [9–10] via shifting the axle back and forth within the wheel. The Stoddart group synthesized the first rotaxane-based molecular shuttle in 1991 [11]. It consisted of a tetracationic wheel, which was able to move back and forth between two identical hydroquinol stations. These symmetrically surrounded a polyether axle, which was terminated at the ends by large triisopropylsilyl stoppers [11]. Other rotaxane systems were also studied. For instance, Leigh and coworkers synthesized several rotaxane shuttles in the last years [12]. Many of these rotaxanes are based on a benzylic amide macrocycle with isophtalamide units building up twofold hydrogen bonds to an acceptor axle. The Schalley group often used a similar hydrogen bond motif for the design of molecular shuttles with the Vögtle–Hunter tetralactam macrocycle next to several other combinations [13–15]. Fernandes et al. recently published a further interesting application of rotaxanes [16–17]. The axle consisted of a peptide, which can be released from the wheel by the according reaction, thus allowing the rotaxane to function as a high-precision delivery system. The authors introduced a system, which – in contrast to the first generation of these kinds of rotaxanes – showed water solubility and contained appropriate locations for substitutions in order to improve its properties [16].

Theoretical investigations on rotaxanes accompanied or even preceded experimental work frequently, thus showing that theory offers many viable tools for the understanding and the development of rotaxanes. Zerbetto et al. showed that the shuttling motion can be separated from the other degrees of freedom, and that the effective coordinate of the motion can be described as a double-minimum potential [18]. The co-conformer stability for rotaxane based molecular shuttles was investigated by means of molecular modeling [19]. The Peyerimhoff group has carried out an in depth study of the rotaxane formation [20]. A later study investigated the shuttling motion of the wheel as a one-dimensional translation, together with the influence of the Kohn–Sham frontier orbitals of wheel and axle upon conductivity and electron tunneling along the rotaxane [21]. A quantum chemical shuttling motion study of rotaxane-based molecular switching devices has revealed how the modification of the redox states of both entities results in changes of the computational energy profile [22]. The formation of α-cyclodextrin-based [3]pseudorotaxanes in the gas phase was studied by means of density functional calculations [23]. Molecular mechanics calculations were used for a free energy calculation of an α-cyclodextrin rotaxane system and for the investigation of low-barrier molecular rotary motors with rotaxane architecture [24]. The co-conformational selectivity of two dibenzo-24-crown-8 macrocycles to ammonia binding sites in a [3]rotaxane [25], and the hydrogen bonding strength in polymeric urethane rotaxanes in a mean-field model [26] were investigated by semiempirical methods.

In our groups, we investigated the main binding motif for rotaxane systems of the Vögtle [27–30], Schalley- [29–31], and Leigh-type [27]. We performed an energetic and vibrational analysis for the twofold hydrogen bonds in order to understand the binding pattern [28]. A close relationship between the strength of the hydrogen bond and the charge of the acceptor oxygen was detected [32]. Substitution with electron-withdrawing groups weakens the twofold hydrogen bond, whereas substitution with electron-donating groups led to an increase of interaction energy. In the vibrational spectra, the red shift for both the C=O stretching mode and the N–H stretching mode was correlated to the binding energies of the hydrogen bonds [27]. Compared with single hydrogen bonds, the twofold hydrogen bonds showed shorter red shifts for the N–H stretch modes but larger red shifts for the C=O stretch mode [27]. Different density functionals, including one functional with an empirical correction for dispersion interaction, for the treatment of such rotaxane complexes were studied. We compared these density functional theory (DFT) results with Møller–Plesset second-order perturbation theory (MP2) calculations [29]. The contribution of the London dispersion interaction to the total interaction energy in the gas phase is of the same magnitude as the hydrogen bonding interaction (about −14 kcal/mol).

The molecular functionality of rotaxanes is solely based on the interplay of different non-covalent interactions between the axle and the wheel. Therefore, the understanding of these (mostly) attractive forces is crucial for the development of the field. Also, by understanding how one can modify or even tune the axle–wheel interplay, rotaxanes for different purposes can be designed enhancing the applicability of such materials. This study aims to understand rotaxanes with respect to its non-covalent interactions on the molecular level and to contribute to a more rational design of new molecular machines. Therefore, we investigated the energetics by substitution the rotaxanes with different functional groups and by changing the degree of molecular flexibility.

## Computational methodologies

The structures of all compounds were fully optimized without any symmetry constraints. Density functional theory (DFT) with the gradient-corrected meta-functional TPSS combined with the resolution of identity technique (RI) and the def2-TZVP basis set were applied [33–34] together with the dispersion correction D3 [35–36]. This level of theory is abbreviated as TPSS-D3/def2-TZVP. All molecular calculations were performed by using the TURBOMOLE 6.4 program package [33]. The convergence criterion for the geometry optimization was set to 10^−4^ atomic units for the norm of the Cartesian gradient. The SCF-convergence was set to 10^−6^ atomic units.

The adiabatic complex interaction energies Δ*E*^int^ were calculated according to the supramolecular approach by subtracting the energies of the relaxed monomers 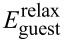, 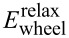 from the total complex energy *E*^tot^ [37–38].

[1]
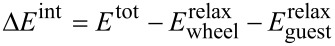


Interaction energies were counterpoise-corrected by the procedure introduced by Boys and Bernardi. The basis set superposition error (BSSE) does not exceed 3 kcal/mol (about 5% of Δ*E*^int^) for any of the complexes calculated. In order to confirm the nature of the stationary point obtained, we performed an analytical frequency analysis with the aoforce module [39–41] resulting in only positive values for the minima. As a first approximation to solvation, we applied the conductor-like screening model (COSMO) [42]. This a continuum solvation model, where the solute molecule forms a cavity within the dielectric continuum of permittivity ε that represents the solvent, and which neglects the cavitation and the solute–solvent dispersion term. For ε we chose 4.806 which is the value of chloroform. The distance of solvents to van der Waals radii of the atom (standard values were chosen here) was set to 1.3 Å. The Hammett parameters are taken from [43].

### Computational details for periodic calculation

The periodic calculations were carried out with the Vienna ab-initio simulation package VASP 5.3 [44–45]. We utilized the GGA functional PBE [46] in combination with a projector-augmented plane wave basis set (PAW) [47–48] with energy cutoff of 1000 eV. The Brillouin zone was sampled with a Γ-centered 2 *×* 1 *×* 1 *k*-mesh. The crystal was fully optimized (including cell parameters) until all forces were below 0.005 eV/Å. The PBE functional was corrected for missing non-local correlation interactions through the atom-pairwise London dispersion correction D3 in the Becke–Johnson damping scheme [35–36]. A single (isolated) dimer was optimized with the same technical setup in a large unit cell with minimum intermolecular atom-atom distance of 16 Å. This method combination provides reliable results for both the gas phase and the solid state as shown in a number of publications by us [49–51] and other groups [45,52].

## Structures under study

The hereby considered pseuodorotaxanes (Figure 1) consist of an amide axle inside the cavity of a macrocycle, which contains two isophtalamide units. One kind of the investigated axles is a fumaramide derivative with a C=C double bond and two connected amide groups (labeled as Leigh-type-DB, **DB** throughout the article) [2,53], and the other kind is a succinic amide derivative with a C–C single bond, and two connected amide groups (labeled as Leigh-type-SB, **SB** throughout the article) [2,53]. Due to the aforementioned structure of the wheel and the axle, and since the wheel-O=C*···*NH-axle type interplay is prohibited by the substitution of the corresponding hydrogen atoms by either a methyl group (Figure 1 top) or a benzyl group (Figure 1 bottom), only four hydrogen bonds can be formed between the subunits through wheel–NH*···*O=C-axle interactions. At the two different axles with single and double bond, the phenyl groups of the axle will be substituted symmetrically in order to investigate the substitution effect. Moreover, the influence of the flexibility of the axle, see Figure 1 upper part, will be investigated. The rotaxanes with di-phenyl groups are analyzed in order to allow comparison to experimental data.

## Results and Discussion

### Crystal structure

The fully optimized crystal structure agrees very well with the experimental X-ray structure, see Table 1. The unit cell volume (Vol) is smaller than the experimental value by only 1.7%. Typical thermal cell expansions (from calculated 0 K to measured 100 K) are 2–3%. A recent study showed that PBE-D3 (with a large basis set) overestimates molecular sizes by approximately 1% [54]. Therefore, the calculated cell volume is in a reasonable agreement with the experimental value when thermal expansion effects are considered in the comparison. The optimization is performed without symmetry constraints, and the correct space group (monoclinic) is reproduced, i.e., all cell angles differ by less than 0.3° from the X-ray structure.

**Table 1:** Comparison of the X-ray structure of the rotaxane with the computed crystal and gas phase geometries. The structures are optimized at the PBE-D3/1000 eV level (TPSS-D3 with the def2-TZVP basis). The first block shows the cell parameters describing the intermolecular packing, whereas the second block highlights some intramolecular distances and angles (compare with Figure 2). Distances in parentheses denote the corresponding length to the heavy (non-hydrogen) atom.

	Reference	Crystal	Gas phase	
	X-ray	PBE-D3	PBE-D3	TPSS-D3

*a* /Å	9.79	9.69	—	—
*b* /Å	16.16	16.16	—	—
*c* /Å	16.87	16.78	—	—
β /*^°^*	105.0	105.3	—	—
*Vol* /Å^3^	2579	2535	—	—
*R*_1_ /Å	2.01(2.98)	1.97(2.97)	2.11(3.10)	2.11(3.10)
*R*_2_ /Å	2.24(3.16)	2.11(3.11)	2.30(3.28)	2.35(3.31)
*R*_3_ /Å	2.31(3.13)	2.19(3.09)	2.25(3.28)	2.28(3.30)
*R*_4_ /Å	8.24	8.19	9.25	9.11
φ_1_ /*^°^*	−1.7	−1.2	−4.0	−2.2
φ_2_ /*^°^*	5.16	4.4	−9.7	−7.0

**Figure 2:**
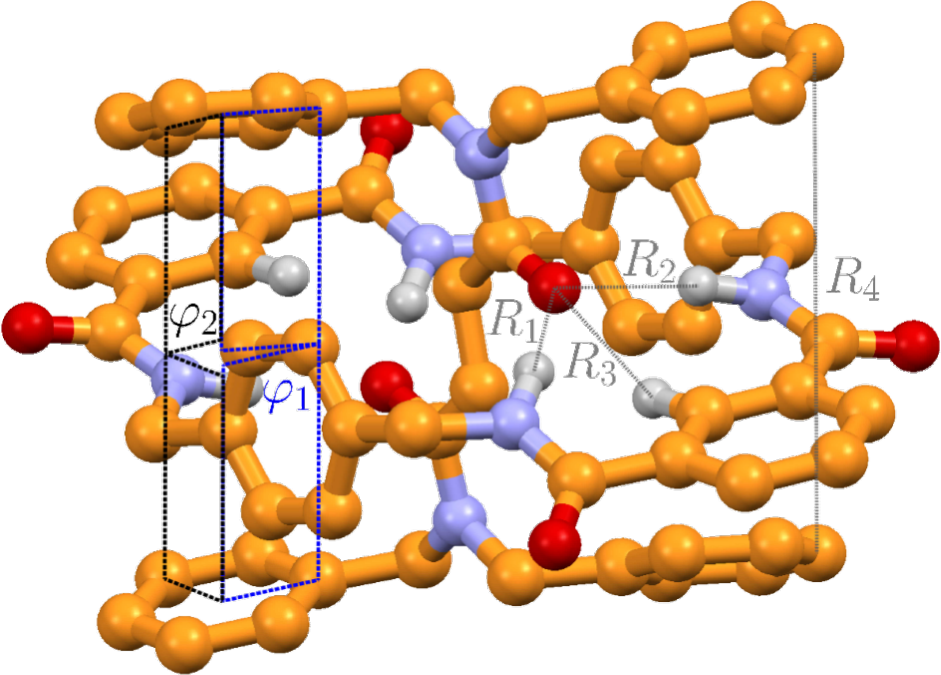
Molecular geometry of one rotaxane optimized in periodic boundaries at the PBE-D3/1000 eV level. Hydrogen atoms are omitted for clarity. Some intramolecular distances and angles are highlighted.

Because the molecular structure is rather flexible, we observe interesting crystal packing effects. We compared the highlighted intramolecular distances and angles from experiment and theory in Figure 2. As a result, we exemplified the influence of non-covalent interactions. The distances *R*_1_, *R*_2_, and *R*_3_ are significantly smaller in the crystal compared to the gas phase structure. The torsion angles φ_1_ and φ_2_ describe the relative tilting between the flexible phenyl rings, which differs by more than 10° between crystal and gas phase. All these geometrical data are very well reproduced by the PBE-D3/1000 eV calculations, see third column in Table 1. However, the gas phase calculations (5th and 6th column in Table 1) show that one has to be careful when comparing calculated gas phase structures with measured crystal geometries.

The calculated lattice energy (for one rotaxane, excluding phonon contributions) of 77.7 kcal/mol is quite large but in a reasonable range for a molecule of this size. Recent benchmark studies showed that lattice energies on the PBE-D3/1000 eV level deviate by less than 9% from (thermal back-corrected) experimental sublimation energies [55]. The excellent agreement of the utilized theoretical method with the X-ray experiment justifies its application in the following sections. Mostly for technical reasons we have chosen PBE-D3 in the solid state calculations but TPSS-D3 in the molecular treatments. According to many benchmark calculations (see, e.g., [49,51,56]), both functionals perform very similar for non-covalent interaction (TPSS-D3 being even somewhat better for hydrogen bonding), which supports the above conclusion.

### Substitution effect

The interaction energies in Table 2 show that the more flexible axle (**SB**) binds stronger to the wheel than the less flexible ethylene-containing axle (**DB**) for all pseudorotaxanes studied. The difference between the substituted **DB** and **SB** amounts to 1–3 kcal/mol. Substituents with −M/−I effect bind more weakly than those with +M/+I effect, which fits neatly to the fact that the axle in this investigated system accepts the hydrogen bond and therefore prefers electrons to be shifted towards the functional group. Interestingly, the substitution effects seem to be almost additive, i.e., for both the **SB** and the **DB** structure changes in the energy range of 5 kcal/mol can be obtained with the appropriate functional group, compare for example **2a@1** to **2h@1** and **3a@1** to **3h@1**.

**Table 2:** Interaction energies *E*_int_ for the different pseudorotaxane systems, labeling see Figure 1. The first two columns list the substituents succeeded by their effects (mesomeric or inductive). The last line gives the values for the di-phenyl structures. In the last column, the Hammett-parameters are given.

			*E*_int_		*E*_int_	σ
			kcal/mol		kcal/mol	

−I, −M	*p*-NO_2_	**2a@1**	−41.2	**3a@1**	−43.1	0.78
−I	*p*-CF_3_	**2b@1**	−42.4	**3b@1**	−44.9	0.54
−I,(+M)	*p*-Cl	**2c@1**	−43.0	**3c@1**	−45.4	0.23
+I,(−M)	*p*-SiH_3_	**2d@1**	−44.1	**3d@1**	−45.9	0.10
—	*p*-H	**2e@1**	−44.7	**3e@1**	−46.5	0.00
+I	*p*-*t*-Bu	**2f@1**	−44.9	**3f@1**	−47.6	−0.20
−I, +M	*p*-OH	**2g@1**	−45.7	**3g@1**	−46.8	−0.37
−I, +M	*p*-NH_2_	**2h@1**	−46.5	**3h@1**	−48.4	−0.66
−I, −M	*m*-NO_2_	**2a**'**@1**	−43.7	**3a**'**@1**	−45.5	0.71
−I, +M	*m*-OH	**2g**'**@1**	−45.1	**3g**'**@1**	−47.5	0.12
	*p*-2Ph	**4@1**	−56.1	**5@1**	−58.7	—

**Figure 3:**
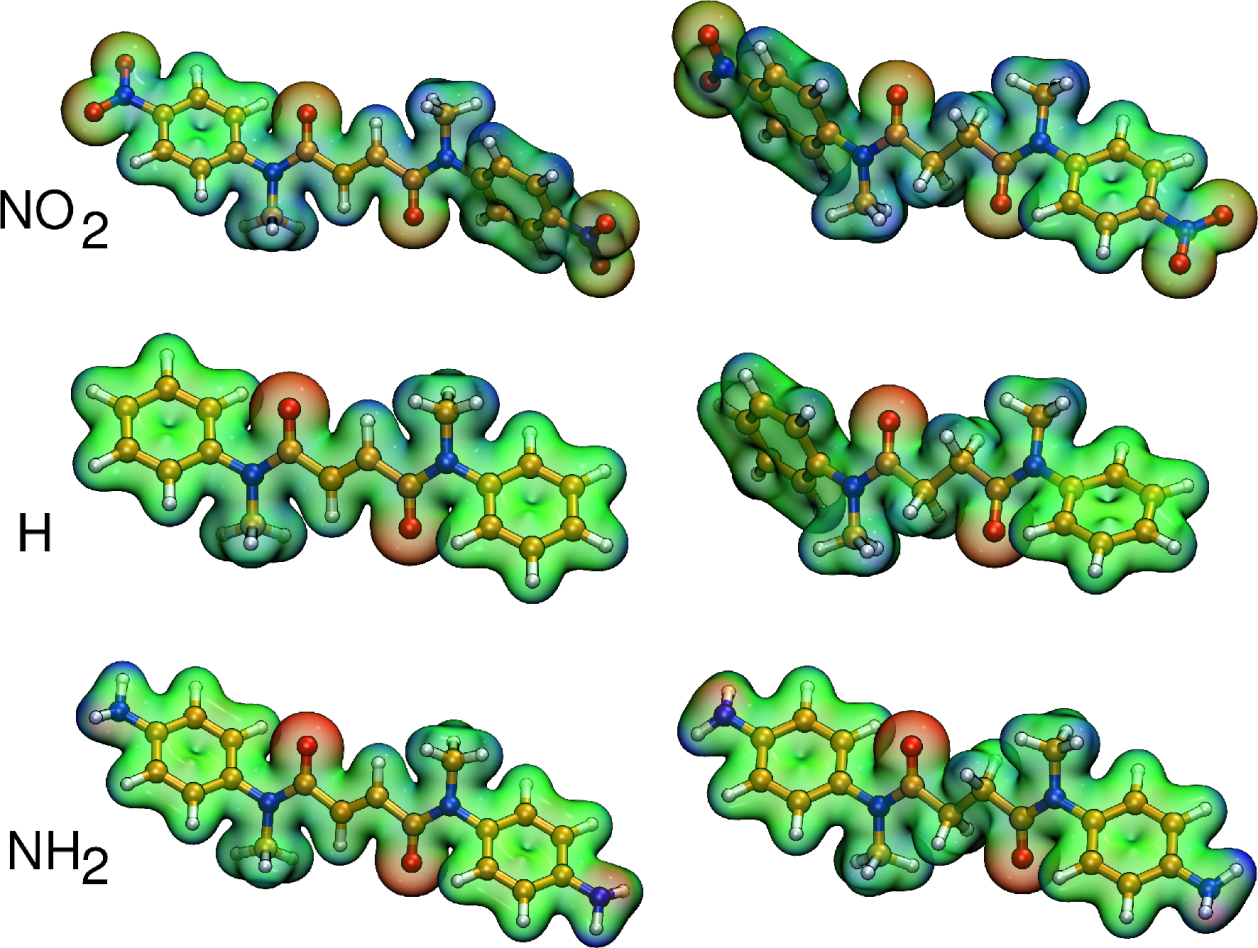
Electrostatic potential for the complexes **2a@1** (top left), **2e@1** (middle left), **2h@1** (lower left) and **3a@1** (top right), **3e@1** (middle right), **3h@1** (lower right).

A qualitative insight of the varying binding situation can be gained from the electrostatic potential shown for six rotaxanes in Figure 3. The electron withdrawing groups reduce the hydrogen bond accepting character of the oxygen atoms (see reduced red color and increase in blue color of **2a@1** in Figure 3 compared to **2e@1**), the π-electron donating groups, on the other hand, increase the hydrogen bond accepting character (see more pronounced red areas and less pronounced blue color of **2f@1** in Figure 3 compared to **2e@1**).

In order to understand the origin of the different binding energies, we consider the most direct interaction between axle and wheel, namely the hydrogen bond accepted by the axle and donated from the wheel, as possible influence. Since the wheel and the axle are symmetric, and there are two recognition sites between axle and wheel, the latter sites strongly resemble each other in geometrical parameters. Thus, we only consider one binding site (isophtalic unit) with its hydrogen bonds. Note, that the hydrogen bond in the **DB** systems are more symmetrical than in **SB** systems. As the choice of the binding site is sort of arbitrary, we always choose the one with the shortest N–H*···*O distance. The full data can be found in the Supporting Information File 1.

The hydrogen bonds listed in Table 3 fall in the range of 2.1 to 2.4 Å, and their angles range from 150 to 180 degrees. The hydrogen bonds are only roughly correlated to the strength of the interaction between axle and wheel, i.e., the variations within different substitutions are too small to discuss them within the error of the method. However, a general shortening of the hydrogen bonds with increasing energies is visible, compare **2a@1**, **2e@1** and **2h@1**. Moreover, there are more symmetrical hydrogen bonding situations in the middle of the listed data in Table 3 (e.g., **2e@1**). Furthermore, there is a significant difference (0.1–0.2 Å) between **SB** and **DB**. Considering these two(three)-fold hydrogen bonds, **SB** is less symmetric indicated by the shorter short N–H*···*O bond and the longer long N–H*···*O bond compared to **DB**. The di-phenyl rotaxanes exhibit the opposite trend, the shortest and longest hydrogen bond is given in **4@1**. The given energy trend is maintained, the longest N–H*···*O bond in **4@1** is much longer (2.5 Å) than other long N–H*···*O bonds. This also shows the importance of such stopper groups for the interactions within the rotaxanes, as they have notable electronic influence. Thus, their role is not necessarily restricted to the mere mechanical prevention of a dethreading of the axle, which was also witnessed for diketopiperazine-based amide rotaxanes [57].

**Table 3:** Hydrogen bond distances in Å for the different pseudorotaxane systems, for labeling see Figure 2. The second and third last lines show the substitution at the meta-position.

		*R*_1_	*R*_2_	*R*_3_		*R*_1_	*R*_2_	*R*_3_

*p*-NO_2_	**2a@1**	2.24	2.40	2.29	**3a@1**	2.18	2.42	2.26
*p*-CF_3_	**2b@1**	2.21	2.38	2.27	**3b@1**	2.16	2.43	2.24
*p*-Cl	**2c@1**	2.23	2.29	2.26	**3c@1**	2.15	2.35	2.22
*p*-SiH_3_	**2d@1**	2.17	2.35	2.22	**3d@1**	2.15	2.34	2.21
*p*-H	**2e@1**	2.21	2.22	2.20	**3e@1**	2.14	2.33	2.20
*p*-*t*-Bu	**2f@1**	2.21	2.27	2.24	**3f@1**	2.11	2.39	2.20
*p*-OH	**2g@1**	2.08	2.18	2.34	**3g@1**	2.14	2.30	2.19
*p*-NH_2_	**2h@1**	2.15	2.22	2.17	**3h@1**	2.11	2.34	2.18
*m*-NO_2_	**2a**'**@1**	2.23	2.44	2.32	**3a**'**@1**	2.14	2.63	2.31
*m*-OH	**2g**'**@1**	2.26	2.22	2.22	**3g**'**@1**	2.13	2.38	2.22
*p*-2Ph	**4@1**	2.04	2.50	2.27	**5@1**	2.11	2.35	2.28

**Figure 4:**
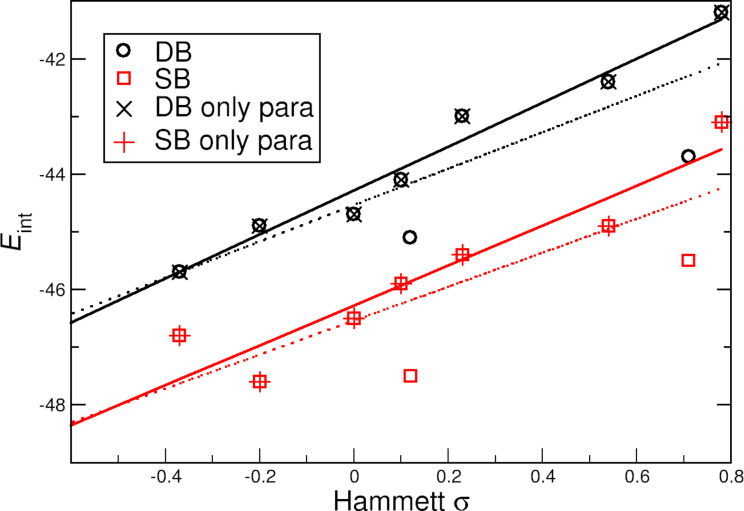
Interaction energies plotted against the Hammett σ parameters. The values are given in Table 1. Black curves: **DB** and red curves: **SB**. The solid lines are fits to all energies of the para-substitution only, the dotted lines are linear regressions to all interaction energies.

In Table 2, also the Hammett σ parameters are given. These substituent parameters [43] are the difference of the p*K*_a_ values of substituted and non-substituted benzoic acids, they can be correlated with the interaction energies resulting in good correlation coefficients of 0.9880 (**DB**) and 0.9596 (**SB**) if only the para-positions are considered, see Figure 4. This fitted linear regression curve are as follows:

[2]
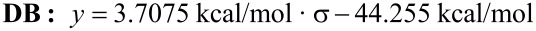


[3]
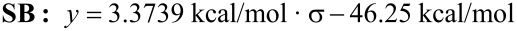


Equation 2 and Equation 3 can be used to estimate the contributions of different substituents given the σ-values are provided.

### Solvent effects

As expected, the presence of the solvent decreases the binding energy by 11–14 kcal/mol, see Table 4. Even though the trends in the difference between single and double bond binding energy is constantly 1–4 kcal/mol. By comparison of the total energies of the complex, separated wheel, and axle, this can be assigned to the stabilization of the complex and wheel by approximately 20 kcal/mol, whereas the axle is only stabilized by 10 kcal/mol.

**Table 4:** Interaction energies *E*_int_ for the different pseudorotaxane systems applying a solvent model, labeling see Figure 1. The first two columns list the substituents succeeded by their effects (mesomeric or inductive) as in Table 2.

			*E*_int_		*E*_int_
			kcal/mol		kcal/mol

−I, −M	*p*-NO_2_	**2a@1**	−29.6	**3a@1**	−31.0
—	*p*-H	**2e@1**	−32.4	**3e@1**	−33.8
−I, +M	*p*-NH_2_	**2h@1**	−33.7	**3h@1**	−35.1
	*p*-2Ph	**4@1**	−42.1	**5@1**	−45.6

In the following, we focus on the hydrogen bonded systems and repeat the previously described distances (Table 3) for the corresponding solvated systems in Table 5. Again the distances roughly follow the trend that with increasing binding energy the distances are shorter.

**Table 5:** Hydrogen bond geometry in Å for the different pseudorotaxane systems with solvent model, labeling see Figure 2. The second and third last lines show the substitution at the meta-position.

		*R*_1_	*R*_2_	*R*_3_		*R*_1_	*R*_2_	*R*_3_

*p*-NO_2_	**2a@1**	2.19	2.41	2.30	**3a@1**	2.12	2.47	2.27
*p*-H	**2e@1**	2.11	2.16	2.16	**3e@1**	2.12	2.28	2.19
*p*-NH_2_	**2h@1**	2.08	2.14	2.13	**3h@1**	2.09	2.18	2.14
*p*-2Ph	**4@1**	2.02	2.43	2.20	**5@1**	2.07	2.27	2.23

The hydrogen bonding situation in the complexes with single bond is still less symmetrical, but the shorter N-H*···*O bond for the solvated is not shorter than in the **DB** complexes. Typically, the distances in the solvated complexes are shorter up to 0.16 Å compared to the unsolvated systems. Considering the reduced binding energies, this is unusual. Comparing different intra- and intermolecular bonds, it appears that this arrangement of shorter and thus stronger hydrogen bonds stems from a more bowed axle with respect to the wheel.

## Conclusion

We investigated several rotaxanes by static quantum chemical calculations in order to gain insight into the interplay of different non-covalent interactions. Therefore, we studied the substitution of the rotaxanes with different functional groups and the degree of molecular flexibility by changing within the axle from a single C–C bond to a double C=C bond. In order to assess the methodology used, we calculated the crystal structure and found a very good agreement with the experiment. For instance, deviations of the unit cell volume were less than 2%. However, care has to be taken when comparing results calculated in the gas phase with those obtained in the condensed phase due to non-local crystal packing effects.

The computed DFT-D3 formation energies of the non-covalently bound rotaxanes in the gas phase range from about −41 to −58 kcal/mol which is typical for supramolecular complexes of this size [50]. For the investigated modified axles, we found that – as expected – a more flexible axle binds stronger than the stiffer axle. Exchanging a double with a single bond leads to an increase of absolute value in binding energy of 1–3 kcal/mol. Alterations of the binding energy in the range of 5 kcal/mol could be achieved for substitution with different functional groups. Thus, it is possible to modulate the rotaxane binding by changing different chemical parts in the region of 1–5 kcal/mol, which should show an influence of the inter-related motion as well. We also investigated the hydrogen bond geometry between the isophtalic unit and the carbonyl oxygen atoms of the axle and found distances in the range of 2.1 to 2.4 Å for 6 contact points. This shows that to a large amount the interactions in the investigated rotaxanes are governed by hydrogen bonding. On the one hand, the single bound complex usually is less symmetric in exhibiting one short and one long N-H*···*O bond than the double bond containing complex. On the other hand, the opposite is observed for the experimentally investigated axle with the four phenyl stoppers. One might assume that the terminal groups play a minor role in the interplay within the rotaxane and serve only to prevent the axle mechanically from dethreading. However, we clearly demonstrated the importance of such rotaxanes parts as the stopper groups also for intra-molecular interactions of the rotaxanes.

Considering an implicit solvent model (COSMO), the complex binding is weakened by approximately 10 kcal/mol. This is due to the fact that the individual parts of the rotaxane are differently stabilized in the solvent. Thus, the axle is less stabilized than the wheel and the complex. Interestingly, we observed slightly shortened (for up to 0.2 Å) hydrogen bonds for all investigated systems. This is supported by a more tilted axle in the solvent.

In future, we plan to explicitly study the different motions within such complex systems. The main focus is on the influence of simple chemical differences such as substitution or dealkylation.

## Acknowledgements

We would like to thank the DFG in the framework of the collaborative research center SFB 624 “Templates” at the University of Bonn for the funding of this research. Furthermore, the financial support of the DFG project KI 768/7-1 is gratefully acknowledged. Finally, the authors would like to add personal thanks to Christoph A. Schalley for helpful discussions and Barbara Intemann for correcting the manuscript.

## Supporting Information

File 1 Geometry and structure data.
