# Supplementary material for: Substitution effect and effect of axle’s flexibility at (pseudo-)rotaxanes
Source: Beilstein J Org Chem. 2014 Jun 5;10:1299–307. doi: 10.3762/bjoc.10.131 (PMC4077404; doi:10.3762/bjoc.10.131)
Supplement: File 1 — Geometry and structure data. [file Beilstein_J_Org_Chem-10-1299-s001.pdf]

Supporting Information

for

Substitution effect and effect of axle's flexibility at (pseudo-)rotaxanes

Friedrich Mahlberg, Jan Gerit Brandenburg, Werner Reckien, Oldamur Hollóczki, Stefan Grimme* and Barbara Kirchner*

Address: Mulliken Center for Theoretical Chemistry, Rheinische Friedrich-Wilhelms-Universität Bonn, Beringstr. 4, 53115 Bonn, Germany

Email: Stefan Grimme* - grimme@thch.uni-bonn.de,

Barbara Kirchner* - kirchner@thch.uni-bonn.de

* Corresponding author

Geometry and structure data

Appendix

0.1 H-bond geometry data

H-bond data is collected starting with the lower label number of the axles oxygens, from this the left and then the right is taken when the oxygen is oriented from above, after this the CH$\cdots$O bond in the middle is collected.

	subst.	label 1	$R_{(NH\cdots O)}$	$a_{(NH\cdots O)}$	$R_{(NH\cdots O)}$	$a_{(NH\cdots O)}$	$R_{(CH\cdots O)}$	$a_{(CH\cdots O)}$	label 2	$R_{(NH\cdots O)}$	$a_{(NH\cdots O)}$	$R_{(NH\cdots O)}$	$a_{(NH\cdots O)}$	$R_{(CH\cdots O)}$	$a_{(CH\cdots O)}$
	NO2	12	227.2	158	234.8	155	229.4	169	15	239.5	152	224.2	162	229.1	172
	CF3	41	221.1	163	238.0	153	226.8	173	55	234.2	156	224.7	158	227.3	169
	Cl	12	227.9	163	223.1	157	225.7	172	14	223.3	157	227.9	162	225.7	172
	SiH3	41	216.6	160	235.1	156	221.7	171	54	235.1	156	216.6	160	221.7	171
p	H	12	221.2	160	222.3	163	220.1	176	13	222.0	164	221.3	160	220.1	177
	tBu	69	226.5	164	221.3	159	224.2	174	76	221.0	160	226.9	164	224.3	174
	OH	41	208.1	167	217.8	172	234.0	142	62	218.0	172	208.4	167	233.3	143
	NH2	41	222.0	165	215.4	162	216.6	178	54	215.3	162	222.3	165	216.6	177
	NO2	12	243.8	155	222.8	157	231.8	172	15	222.7	157	243.5	155	231.7	172
	OH	12	226.2	159	223.0	160	223.9	171	14	226.1	162	221.6	160	222.0	177
	2-Ph	20	203.7	166	249.8	163	226.5	172	21	249.8	163	203.7	166	226.5	172

Table 1: Double bond

p

subst.	label 1	$R_{(NH\cdots O)}$	$a_{(NH\cdots O)}$	$R_{(NH\cdots O)}$	$a_{(NH\cdots O)}$	$R_{(CH\cdots O)}$	$a_{(CH\cdots O)}$	label 2	$R_{(NH\cdots O)}$	$a_{(NH\cdots O)}$	$R_{(NH\cdots O)}$	$a_{(NH\cdots O)}$	$R_{(CH\cdots O)}$	$a_{(CH\cdots O)}$
NO2	41	246.2	155	221.5	156	227.0	174	46	217.8	161	241.9	154	225.9	176
CF3	12	216.4	157	243.3	156	224.4	172	18	241.7	156	217.2	157	225.1	172
Cl	41	240.3	158	218.7	156	223.8	174	46	215.2	162	235.4	157	221.9	176
SiH3	41	239.1	159	219.2	157	223.1	174	46	215.1	163	234.3	157	221.3	176
H	41	238.4	159	217.3	157	222.8	173	46	213.8	163	233.2	158	220.3	176
tBu	78	238.7	159	211.1	159	220.0	174	98	212.3	159	237.3	159	219.0	174
OH	41	239.8	161	215.8	158	222.8	174	46	213.9	164	230.2	158	218.9	178
NH2	41	212.6	159	231.9	160	217.0	172	46	233.5	161	211.2	159	218.1	171
NO2	11	240.1	155	225.2	156	227.8	177	23	213.8	155	262.5	149	230.5	171
OH	41	213.9	158	237.8	158	221.8	172	48	237.9	158	213.3	159	221.6	172
2-ph	41	235.0	158	211.3	163	227.7	156	50	211.3	163	235.0	158	227.7	156

Table 2: Single bond

	subst.	label 1	$R_{(NH\cdots O)}$	$a_{(NH\cdots O)}$	$R_{(NH\cdots O)}$	$a_{(NH\cdots O)}$	$R_{(CH\cdots O)}$	$a_{(CH\cdots O)}$	label 2	$R_{(NH\cdots O)}$	$a_{(NH\cdots O)}$	$R_{(NH\cdots O)}$	$a_{(NH\cdots O)}$	$R_{(CH\cdots O)}$	$a_{(CH\cdots O)}$
	NO2	12	221.6	157	236.5	152	228.8	165	15	240.9	152	218.9	160	229.8	167
p	H	12	215.9	159	210.6	166	215.9	168	13	210.5	166	216.0	159	215.9	168
	NH2	41	208.3	168	213.7	161	213.0	171	54	213.5	162	208.3	168	213.0	171
	2-ph	20	201.6	165	242.6	158	220.1	169	21	242.7	158	201.5	165	220.0	169

Table 3: Double bond Cosmo (Chloroform 4.806 Dielektrizitätskonstante)

	subst.	label 1	$R_{(NH \cdots O)}$	$a_{(NH \cdots O)}$	$R_{(NH \cdots O)}$	$a_{(NH \cdots O)}$	$R_{(CH \cdots O)}$	$a_{(CH \cdots O)}$	label 2	$R_{(NH \cdots O)}$	$a_{(NH \cdots O)}$	$R_{(NH \cdots O)}$	$a_{(NH \cdots O)}$	$R_{(CH \cdots O)}$	$a_{(CH \cdots O)}$
	NO2	41	248.1	153	216.2	154	226.0	169	46	212.0	160	247.1	152	226.8	171
p	H	41	234.1	159	211.7	157	220.7	167	46	211.6	162	228.4	159	219.1	171
	NH2	41	208.8	159	218.4	163	213.5	165	46	218.3	163	208.9	159	213.4	165
	2-ph	41	226.9	159	206.8	165	223.1	156	50	207.0	165	226.5	160	223.2	156

Table 4: Single bond Cosmo (Chloroform 4.806)

0.2 Gas phase results on the structures with the C–C axle

Table 5: Cartesian coordinates and total energy in a.u. of complex **3a@1**

E(RI-TPSS-D3/def2-TZVP)=-3125.071271770

C	-4.7537372	1.1897337	0.2959751
C	-4.0276960	0.0620943	0.6927430
C	-4.6972972	-1.1558679	0.8654380
C	-6.0700522	-1.2391037	0.6437989
C	-6.7869868	-0.1136356	0.2418172
C	-6.1330795	1.1132058	0.0750797
C	-2.5346512	0.0691819	0.8782682
O	-1.8923135	-0.9825600	0.9040394
C	-6.9636989	2.2917994	-0.3459145
O	-8.0813913	2.1451032	-0.8502234
N	-1.9375638	1.2955074	0.9975546
C	-0.4946982	1.4408843	0.8699998
C	-0.0698294	1.8084577	-0.5432165
C	-0.8003609	1.3736840	-1.6536947
C	-0.4034925	1.7158613	-2.9443095
C	0.7346284	2.4992629	-3.1598752
C	1.4825127	2.9151022	-2.0509767
C	1.0811197	2.5728514	-0.7608695
C	1.1100964	2.9506154	-4.5553602
N	0.5157026	4.2532882	-4.8791104
C	1.1335651	5.4179659	-4.5249358
O	2.2549705	5.4342303	-4.0051093
C	0.3849942	6.6900911	-4.8032805
C	-0.9964192	6.7225921	-5.0157883
C	-1.6644815	7.9341537	-5.2206931

C	-0.9299610	9.1267587	-5.2177636
C	0.4485265	9.1004623	-5.0131813
C	1.1055695	7.8905595	-4.7965594
C	-3.1551780	8.0300543	-5.3974852
O	-3.7382460	9.1153788	-5.3396111
N	-3.8181011	6.8528414	-5.6207781
C	-5.2771595	6.7847600	-5.6045164
C	-5.8007779	6.2684432	-4.2784636
C	-5.4169723	6.8990761	-3.0882840
C	-5.8694311	6.4248471	-1.8602998
C	-6.7232429	5.3182580	-1.7902368
C	-7.1247272	4.6994755	-2.9804754
C	-6.6598481	5.1670696	-4.2098126
C	-7.1550574	4.7546580	-0.4539488
N	-6.4282641	3.5284656	-0.1128396
O	-3.0641254	4.0640980	-0.0491510
C	-2.3187594	4.6983950	-0.8089146
C	-2.4901921	4.6392552	-2.3186512
C	-3.5448362	3.6101748	-2.6943357
C	-3.5617044	3.3271016	-4.1826586
O	-2.8267367	3.9260681	-4.9822290
N	-1.2626828	5.4563279	-0.3459509
C	-0.3877489	6.1755605	-1.2954230
C	-0.8463330	5.3963762	1.0134834
C	-1.7707348	5.3350344	2.0700865
C	-1.3235166	5.2564547	3.3828202
C	0.0450347	5.2484971	3.6388315
C	0.9793744	5.3322649	2.6109709
C	0.5295938	5.4069368	1.2996425
N	0.5179797	5.1579977	5.0354958

N	-4.4250436	2.3321557	-4.5867541
C	-4.4794486	1.9058203	-5.9450525
C	-4.5059379	2.8329615	-6.9974619
C	-4.5889164	2.3923600	-8.3114964
C	-4.6469630	1.0239879	-8.5661487
C	-4.6252710	0.0857610	-7.5389050
C	-4.5390330	0.5325360	-6.2262611
N	-4.7393531	0.5550406	-9.9651726
C	-5.2330665	1.5880754	-3.5979534
H	1.0127657	10.0286727	-5.0164109
H	2.1750116	7.8496473	-4.6152470
H	-1.5607636	5.7968476	-4.9680256
H	-1.4637096	10.0596666	-5.3695574
H	-3.3210783	5.9704051	-5.5470676
H	-0.4423386	4.2601857	-5.2105872
H	0.7596410	2.2327615	-5.3024159
H	2.1929542	3.0598357	-4.6490627
H	-5.6333062	7.8022293	-5.7912805
H	-5.6191251	6.1446388	-6.4246249
H	-0.9880015	1.3743589	-3.7966455
H	-1.6820044	0.7568865	-1.5071081
H	1.6705616	2.9022424	0.0916892
H	2.3674902	3.5245396	-2.2102565
H	-4.7657621	7.7686010	-3.1326285
H	-5.5587880	6.9207833	-0.9429997
H	-7.7928833	3.8432290	-2.9316759
H	-6.9704554	4.6709307	-5.1272004
H	-0.1458391	2.2029121	1.5747324
H	-0.0571459	0.4796180	1.1583057
H	-8.2150663	4.4898164	-0.4684464

H	-6.9833422	5.4857850	0.3420343
H	-2.4866704	2.1421039	0.8891655
H	-5.4715621	3.6187295	0.2099611
H	-4.2339559	2.1248570	0.1088963
H	-6.5839262	-2.1861222	0.7822057
H	-7.8548621	-0.1587132	0.0524814
H	-4.1162919	-2.0235758	1.1617694
H	-1.5301592	4.3822762	-2.7795676
H	-4.5368936	3.9493063	-2.3789176
H	1.2539512	5.4488241	0.4947337
H	2.0373151	5.3265414	2.8451611
H	-2.0211322	5.2114492	4.2107229
H	-2.8308171	5.3527587	1.8633748
H	-4.5042261	-0.1909585	-5.4183671
H	-4.6659425	-0.9707507	-7.7757487
H	-4.6202805	3.0911561	-9.1390314
H	-4.4687525	3.8922813	-6.7846760
H	-0.9863903	6.5998711	-2.0999212
H	0.0952252	6.9959829	-0.7642876
H	0.3745421	5.5170822	-1.7247427
H	-6.0313455	1.0686019	-4.1266282
H	-4.6319624	0.8620799	-3.0402713
H	-5.6921582	2.2864135	-2.8985698
H	-3.3566483	2.6771888	-2.1522641
H	-2.7703627	5.6263006	-2.7046405
O	-0.3354054	5.0618804	5.9227071
O	1.7379307	5.1830580	5.2281114
O	-4.8027769	-0.6634852	-10.1558896
O	-4.7479599	1.4109724	-10.8549863

Table 6: Cartesian coordinates and total energy in a.u. of complex **3b@1**

E(RI-TPSS-D3/def2-TZVP)=-3390.259594367

C	-4.8467921	0.6542763	-0.3364292
C	-4.2873751	-0.3909424	0.4137781
C	-5.0382962	-0.9818834	1.4376406
C	-6.3170431	-0.5175326	1.7330106
C	-6.8564519	0.5406269	1.0049002
C	-6.1210861	1.1158962	-0.0351095
N	-2.9673145	-0.8700214	0.1588425
C	-2.7058517	-2.3071282	0.3764675
C	-8.2217024	1.0883679	1.3145463
F	-8.8504425	0.4026172	2.3012944
C	-1.9289263	0.0036329	-0.0682072
O	-2.1095588	1.2184595	-0.2449534
C	-0.5336251	-0.5953858	-0.0500728
C	0.5212255	0.4956392	-0.1304152
C	1.9239390	-0.0745176	-0.0498180
N	2.9446251	0.8342885	-0.1988450
C	2.6563344	2.2657284	-0.4233843
O	2.1328715	-1.2865744	0.1183083
C	4.3004943	0.4060058	-0.3251879
C	4.8408197	-0.5652805	0.5287721
C	6.1670942	-0.9500843	0.3846384
C	6.9682658	-0.3716176	-0.6042800
C	6.4375216	0.6006874	-1.4488583
C	5.1072972	0.9866594	-1.3102990
C	8.4027834	-0.8060482	-0.7215349

F	9.0500440	-0.2012935	-1.7484562
F	9.1082023	-0.5282014	0.4119188
F	8.5091184	-2.1506249	-0.9167810
F	-9.0373373	1.0593115	0.2231677
F	-8.1629305	2.3918013	1.7109038
N	-2.2090153	3.0650193	2.2738582
C	-2.6657176	2.1890180	3.3459783
C	-1.7726217	0.9704880	3.5006350
C	-2.3201444	-0.2964972	3.7226808
C	-1.5034052	-1.4190786	3.8498555
C	-0.1119087	-1.2970063	3.7488799
C	0.4376271	-0.0264662	3.5482359
C	-0.3806831	1.0942441	3.4281734
C	0.7685848	-2.5273887	3.7953581
N	0.7877904	-3.2385304	2.5126988
C	-0.1055856	-4.2351398	2.2386482
O	-0.9370042	-4.6195085	3.0683919
C	-0.0069232	-4.8646662	0.8790768
C	0.6284830	-4.2411955	-0.1990911
C	0.6935964	-4.8623235	-1.4498986
C	0.1055215	-6.1219627	-1.6188716
C	-0.5325439	-6.7464506	-0.5490372
C	-0.5973927	-6.1205594	0.6945722
C	1.3222007	-4.2130899	-2.6522824
O	1.1024061	-4.6321861	-3.7921438
N	2.1341158	-3.1406180	-2.4073612
C	2.6099111	-2.2830000	-3.4873862
C	1.7530707	-1.0392800	-3.6357654
C	2.3326122	0.2270230	-3.7568937
C	1.5412971	1.3684082	-3.8812213

C	0.1446735	1.2656993	-3.8719493
C	-0.4352027	-0.0027372	-3.7643540
C	0.3572874	-1.1423477	-3.6525697
C	-0.7126639	2.5121007	-3.9201884
N	-0.7575250	3.2026138	-2.6274833
C	0.1163523	4.2086200	-2.3259703
O	0.9645211	4.6065660	-3.1319273
C	-0.0287871	4.8338616	-0.9685593
C	0.5291993	6.1025554	-0.7716150
C	0.4166317	6.7298453	0.4677629
C	-0.2362272	6.0938140	1.5215142
C	-0.7909010	4.8206761	1.3410480
C	-0.6787003	4.1982358	0.0940551
C	-1.4303442	4.1593376	2.5314083
O	-1.2443384	4.5862622	3.6740114
H	-0.9843584	-7.7248728	-0.6854871
H	0.1539918	-6.5846447	-2.5997453
H	1.0328492	-3.2414395	-0.0741243
H	-1.0997836	-6.5837760	1.5379919
H	1.3880899	-2.8746989	1.7809514
H	2.2215233	-2.7679634	-1.4671185
H	3.6501557	-2.0040665	-3.2906547
H	2.5765404	-2.8851445	-4.4005570
H	0.4036272	-3.2369391	4.5418793
H	1.7987943	-2.2531006	4.0421907
H	3.4164095	0.3182398	-3.7546237
H	1.9961956	2.3511795	-3.9755939
H	-1.5194205	-0.0992360	-3.7708238
H	-0.1072869	-2.1226928	-3.5850170
H	-1.9351642	-2.4030358	4.0132846

H	-3.3997986	-0.4031368	3.7944032
H	0.0632679	2.0756403	3.2839531
H	1.5185196	0.0869184	3.4845867
H	-1.7390954	2.2598253	-4.2035593
H	-0.3132747	3.2288921	-4.6420092
H	-2.6606695	2.7883093	4.2619135
H	-3.6949660	1.8768220	3.1399515
H	-1.3723158	2.8291312	-1.9130277
H	-2.2734726	2.6917528	1.3319745
H	-1.0566624	3.1891390	-0.0390066
H	0.8429669	7.7182928	0.6136400
H	-0.3209295	6.5572514	2.4995673
H	1.0439631	6.5747119	-1.6024853
H	-0.4145423	-1.2918193	-0.8877892
H	0.3688721	1.2123109	0.6839796
H	-4.6204560	-1.7937755	2.0227947
H	-6.8864620	-0.9730171	2.5356753
H	-6.5518045	1.9251846	-0.6166237
H	-4.2862157	1.1001369	-1.1455646
H	4.6966512	1.7354216	-1.9796434
H	7.0559286	1.0511500	-2.2172993
H	6.5868711	-1.6986281	1.0496903
H	4.2253244	-1.0095961	1.2991954
H	-1.9427025	-2.6548336	-0.3197449
H	-2.3750979	-2.5122300	1.3994311
H	-3.6258578	-2.8565450	0.1745615
H	3.5636767	2.8328864	-0.2161013
H	2.3318010	2.4604754	-1.4499065
H	1.8799288	2.6029274	0.2643432
H	0.4156615	1.0611965	-1.0626557

H	-0.4038158	-1.1825044	0.8655324

Table 7: Cartesian coordinates and total energy in a.u. of complex **3c@1**

E(RI-TPSS-D3/def2-TZVP)=-3635.082636617

C	-4.7269338	1.1172264	0.2692051
C	-4.0097508	-0.0251824	0.6383571
C	-4.6922078	-1.2371154	0.8026703
C	-6.0692428	-1.2991171	0.6008610
C	-6.7778932	-0.1579474	0.2292807
C	-6.1106131	1.0627652	0.0709085
C	-2.5144880	-0.0367872	0.8058918
O	-1.8846100	-1.0971511	0.8188545
C	-6.9336106	2.2612763	-0.3080864
O	-8.0744848	2.1404119	-0.7657015
N	-1.9024848	1.1812612	0.9251132
C	-0.4596992	1.3118766	0.7824027
C	-0.0493622	1.6905652	-0.6322269
C	-0.7991815	1.2767575	-1.7377060
C	-0.4192802	1.6345822	-3.0291802
C	0.7212916	2.4125031	-3.2505077
C	1.4895783	2.8058784	-2.1473324
C	1.1047076	2.4483933	-0.8564433
C	1.0745693	2.8888306	-4.6432827
N	0.4978333	4.2093281	-4.9213341
C	1.1533034	5.3536399	-4.5679454
O	2.2958286	5.3380445	-4.0960533

C	0.4205629	6.6456773	-4.7940041
C	-0.9633900	6.7064080	-4.9810715
C	-1.6135463	7.9326304	-5.1510105
C	-0.8594311	9.1128088	-5.1339990
C	0.5213608	9.0589177	-4.9512311
C	1.1613765	7.8335726	-4.7732718
C	-3.1044145	8.0540290	-5.3084481
O	-3.6696174	9.1479220	-5.2263141
N	-3.7878232	6.8915328	-5.5446492
C	-5.2475351	6.8475735	-5.5137082
C	-5.7637262	6.3099849	-4.1934054
C	-5.3627126	6.9151238	-2.9959232
C	-5.8033098	6.4192604	-1.7723076
C	-6.6618110	5.3158666	-1.7139924
C	-7.0798943	4.7221295	-2.9111255
C	-6.6272910	5.2114655	-4.1365275
C	-7.0793215	4.7278074	-0.3840441
N	-6.3648835	3.4845875	-0.0839942
O	-3.0321439	3.9548500	-0.0410562
C	-2.2800533	4.6049364	-0.7861571
C	-2.4502797	4.5847419	-2.2977940
C	-3.5134906	3.5767424	-2.7044975
C	-3.5344273	3.3364113	-4.2013140
O	-2.8057032	3.9663418	-4.9873007
N	-1.2303930	5.3472984	-0.3043662
C	-0.3398803	6.0779428	-1.2284100
C	-0.8339326	5.2660018	1.0677342
C	-1.7723142	5.2678252	2.1078690
C	-1.3529018	5.1709493	3.4318328
C	0.0076324	5.0820148	3.7231024

C	0.9545975	5.0997928	2.7015154
C	0.5297529	5.1902297	1.3786302
Cl	0.5311272	4.9561163	5.3842758
N	-4.3934839	2.3583266	-4.6319273
C	-4.4451242	1.9764684	-6.0109723
C	-4.5600238	2.9347982	-7.0229918
C	-4.6340963	2.5425622	-8.3560566
C	-4.6005974	1.1854913	-8.6774572
C	-4.4917289	0.2185208	-7.6804041
C	-4.4091872	0.6196925	-6.3488449
Cl	-4.6992614	0.6925051	-10.3498194
C	-5.2087585	1.5853001	-3.6737032
H	1.1011874	9.9775140	-4.9425008
H	2.2331390	7.7703278	-4.6130185
H	-1.5436100	5.7904120	-4.9439566
H	-1.3796391	10.0573779	-5.2584440
H	-3.3061250	5.9980841	-5.4909518
H	-0.4769626	4.2399358	-5.2010062
H	0.6930156	2.1953012	-5.3982717
H	2.1567666	2.9809938	-4.7597146
H	-5.5885601	7.8747791	-5.6734897
H	-5.6093839	6.2315211	-6.3437281
H	-1.0212891	1.3131545	-3.8770975
H	-1.6853291	0.6676067	-1.5864856
H	1.7080824	2.7624759	-0.0078939
H	2.3756383	3.4126438	-2.3112648
H	-4.7049501	7.7801323	-3.0310848
H	-5.4775050	6.8938481	-0.8490044
H	-7.7501058	3.8669008	-2.8713332
H	-6.9488647	4.7330004	-5.0595270

H	-0.0952226	2.0636933	1.4899203
H	-0.0292340	0.3428415	1.0549736
H	-8.1429577	4.4773755	-0.3857445
H	-6.8839775	5.4391790	0.4246229
H	-2.4414047	2.0369262	0.8293697
H	-5.3901963	3.5548777	0.1885597
H	-4.1972673	2.0480348	0.0896843
H	-6.5933007	-2.2416942	0.7319738
H	-7.8494920	-0.1853516	0.0584974
H	-4.1180223	-2.1164299	1.0774324
H	-1.4919435	4.3299650	-2.7635357
H	-4.5026680	3.9154715	-2.3797490
H	1.2706390	5.1851569	0.5866928
H	2.0114692	5.0354755	2.9360523
H	-2.0799882	5.1717566	4.2365311
H	-2.8278250	5.3425127	1.8848703
H	-4.3097627	-0.1328323	-5.5723110
H	-4.4635920	-0.8334680	-7.9418904
H	-4.7255346	3.2837757	-9.1425038
H	-4.5911988	3.9864211	-6.7694169
H	-0.9263258	6.5545012	-2.0133644
H	0.1721926	6.8575900	-0.6641573
H	0.4005213	5.4149739	-1.6888579
H	-5.9939815	1.0738296	-4.2295708
H	-4.6104143	0.8463159	-3.1291962
H	-5.6814865	2.2582878	-2.9575680
H	-3.3342618	2.6274712	-2.1884927
H	-2.7178173	5.5844563	-2.6592173

Table 8: Cartesian coordinates and total energy in a.u. of complex **3d@1**

E(RI-TPSS-D3/def2-TZVP)=-3297.275183438

C	-4.7564519	1.2737280	0.3609315
C	-4.0279531	0.1529453	0.7717149
C	-4.6963926	-1.0617621	0.9696481
C	-6.0709174	-1.1476759	0.7601460
C	-6.7910000	-0.0277826	0.3480893
C	-6.1379295	1.1956259	0.1552026
C	-2.5338451	0.1654169	0.9499554
O	-1.8915953	-0.8864280	1.0021914
C	-6.9738189	2.3706191	-0.2672961
O	-8.1135558	2.2202901	-0.7191540
N	-1.9372391	1.3938830	1.0334937
C	-0.4953698	1.5376259	0.8923888
C	-0.0830307	1.8696447	-0.5334908
C	-0.8257385	1.4127444	-1.6267868
C	-0.4439158	1.7291909	-2.9285295
C	0.6914415	2.5081993	-3.1721531
C	1.4522397	2.9452801	-2.0803238
C	1.0658750	2.6285108	-0.7793181
C	1.0465992	2.9387088	-4.5793513
N	0.4513925	4.2387606	-4.9101145
C	1.0854885	5.4059744	-4.5948718
O	2.2241739	5.4269298	-4.1138368
C	0.3338814	6.6764394	-4.8755644
C	-1.0483741	6.7060010	-5.0814049
C	-1.7176963	7.9132393	-5.3048418
C	-0.9842982	9.1063671	-5.3219129

C	0.3946548	9.0837354	-5.1193860
C	1.0536643	7.8772648	-4.8889086
C	-3.2084774	8.0018654	-5.4856180
O	-3.7927727	9.0886396	-5.4613946
N	-3.8693787	6.8179973	-5.6748246
C	-5.3283001	6.7487753	-5.6513283
C	-5.8433752	6.2644179	-4.3099429
C	-5.4560419	6.9278305	-3.1390955
C	-5.8937125	6.4797914	-1.8960829
C	-6.7363173	5.3676351	-1.7919353
C	-7.1421341	4.7162516	-2.9631511
C	-6.6917540	5.1573688	-4.2074715
C	-7.1468829	4.8301872	-0.4385059
N	-6.4189199	3.6076398	-0.0892987
O	-3.0999946	4.1125796	-0.0514333
C	-2.3498630	4.7457832	-0.8121018
C	-2.5162281	4.6763623	-2.3231883
C	-3.5627542	3.6389582	-2.6978183
C	-3.5655611	3.3340192	-4.1827915
O	-2.8354595	3.9377686	-4.9877458
N	-1.3025696	5.5064113	-0.3511905
C	-0.4260871	6.2229557	-1.2993944
C	-0.8895880	5.4551868	1.0169238
C	-1.8116819	5.4320799	2.0703410
C	-1.3584776	5.3577684	3.3860981
C	0.0094944	5.3143244	3.7022386
C	0.9167050	5.3621762	2.6299171
C	0.4815835	5.4314726	1.3104892
Si	0.5959722	5.2060707	5.4815830
N	-4.4069042	2.3251679	-4.5768297

C	-4.4407130	1.8758943	-5.9357745
C	-4.5431400	2.7823024	-6.9969714
C	-4.6044405	2.3118549	-8.3045098
C	-4.5716493	0.9363939	-8.5994493
C	-4.4716074	0.0465508	-7.5183310
C	-4.4030107	0.5043669	-6.2035004
Si	-4.6658409	0.3063692	-10.3653296
C	-5.2171150	1.5845437	-3.5893429
H	0.9582226	10.0123428	-5.1367129
H	2.1241528	7.8390543	-4.7130379
H	-1.6128530	5.7817744	-5.0172916
H	-1.5189012	10.0364304	-5.4878515
H	-3.3734203	5.9366486	-5.5724401
H	-0.5217717	4.2427259	-5.1973042
H	0.6807075	2.2121054	-5.3106191
H	2.1280607	3.0432950	-4.6923109
H	-5.6869098	7.7606567	-5.8618756
H	-5.6724016	6.0866738	-6.4528416
H	-1.0404116	1.3745819	-3.7670444
H	-1.7079764	0.8025465	-1.4581898
H	1.6619556	2.9781835	0.0601959
H	2.3334443	3.5541852	-2.2615939
H	-4.8102608	7.7995664	-3.2100613
H	-5.5772171	6.9984303	-0.9934567
H	-7.7998292	3.8538391	-2.8876736
H	-7.0011252	4.6330853	-5.1093563
H	-0.1443039	2.3199409	1.5732078
H	-0.0538371	0.5848938	1.2020428
H	-8.2078208	4.5688073	-0.4285130
H	-6.9578112	5.5757578	0.3402857

H	-2.4855779	2.2392849	0.9056173
H	-5.4440937	3.6996847	0.1767144
H	-4.2366301	2.2044528	0.1547866
H	-6.5840983	-2.0923202	0.9169943
H	-7.8609614	-0.0739852	0.1710782
H	-4.1131445	-1.9245076	1.2760356
H	-1.5534255	4.4208783	-2.7790108
H	-4.5594330	3.9772933	-2.3966848
H	1.2107759	5.4471389	0.5077443
H	1.9882789	5.3377046	2.8174907
H	-2.0974047	5.3394768	4.1838596
H	-2.8720975	5.4703044	1.8623595
H	-4.3126453	-0.2068794	-5.3874734
H	-4.4396463	-1.0253955	-7.6983243
H	-4.6880660	3.0393740	-9.1093319
H	-4.5758980	3.8454426	-6.7955650
H	-1.0222797	6.6607368	-2.0991964
H	0.0698590	7.0317365	-0.7624463
H	0.3278253	5.5601468	-1.7377574
H	-5.9859409	1.0302438	-4.1266120
H	-4.6108135	0.8859722	-3.0021434
H	-5.7107436	2.2834509	-2.9130932
H	-3.3764951	2.7147181	-2.1405258
H	-2.7986019	5.6594725	-2.7172347
H	-0.5884381	5.1948991	6.3754927
H	1.3896540	3.9690475	5.6977488
H	1.4596279	6.3664743	5.8202338
H	-4.6104695	-1.1766445	-10.3441767
H	-5.9308963	0.7432365	-11.0102497
H	-3.5354097	0.8317489	-11.1729251

Table 9: Cartesian coordinates and total energy in a.u. of complex **3e@1**

E(RI-TPSS-D3/def2-TZVP)=-2715.812036735

C	-4.7245002	1.1105806	0.2691639
C	-4.0062950	-0.0316930	0.6363674
C	-4.6894050	-1.2418406	0.8104814
C	-6.0683330	-1.3017877	0.6208892
C	-6.7783358	-0.1601532	0.2531370
C	-6.1100736	1.0586306	0.0847405
C	-2.5099382	-0.0433805	0.7939900
O	-1.8804732	-1.1044116	0.8094613
C	-6.9337543	2.2591910	-0.2865872
O	-8.0821646	2.1415037	-0.7260888
N	-1.8975510	1.1748302	0.9029075
C	-0.4552879	1.3059086	0.7560294
C	-0.0489006	1.6790052	-0.6612258
C	-0.7989971	1.2578537	-1.7636778
C	-0.4234926	1.6131520	-3.0572578
C	0.7134891	2.3948696	-3.2834340
C	1.4826601	2.7949028	-2.1831956
C	1.1016085	2.4408369	-0.8903516
C	1.0602491	2.8722333	-4.6774551
N	0.4898090	4.1972193	-4.9462603
C	1.1580481	5.3364651	-4.6001781
O	2.3094626	5.3133872	-4.1506560
C	0.4279452	6.6332998	-4.8077624

C	-0.9568324	6.7002723	-4.9854415
C	-1.6039049	7.9295340	-5.1437047
C	-0.8459934	9.1071730	-5.1218852
C	0.5355546	9.0473877	-4.9464245
C	1.1727432	7.8186075	-4.7823316
C	-3.0949678	8.0557365	-5.2965768
O	-3.6558988	9.1522711	-5.2157545
N	-3.7828472	6.8953191	-5.5283714
C	-5.2426131	6.8565912	-5.4955000
C	-5.7579058	6.3165993	-4.1758886
C	-5.3549474	6.9189695	-2.9777465
C	-5.7936940	6.4204863	-1.7545365
C	-6.6519937	5.3169569	-1.6974527
C	-7.0709510	4.7251267	-2.8951716
C	-6.6205530	5.2172722	-4.1202182
C	-7.0677835	4.7261676	-0.3682579
N	-6.3560889	3.4802741	-0.0744677
O	-3.0370980	3.9391680	-0.0406484
C	-2.2812252	4.5932796	-0.7793892
C	-2.4431652	4.5760942	-2.2921463
C	-3.5059122	3.5710570	-2.7064751
C	-3.5255266	3.3397954	-4.2048423
O	-2.7990369	3.9793644	-4.9860439
N	-1.2370213	5.3360587	-0.2905417
C	-0.3381232	6.0637680	-1.2083333
C	-0.8550144	5.2606562	1.0885445
C	-1.8072995	5.2928327	2.1148098
C	-1.3961003	5.2032798	3.4432084
C	-0.0432194	5.0913727	3.7661892
C	0.9048653	5.0791786	2.7439500

C	0.5043744	5.1621996	1.4115467
H	0.2686717	5.0213754	4.8038362
N	-4.3788297	2.3616589	-4.6414659
C	-4.4291308	1.9885576	-6.0255010
C	-4.5752188	2.9546843	-7.0253346
C	-4.6461913	2.5633663	-8.3603353
C	-4.5803080	1.2129883	-8.7082412
C	-4.4400893	0.2509351	-7.7087250
C	-4.3596369	0.6349517	-6.3706407
H	-4.6398282	0.9136833	-9.7502940
C	-5.1903263	1.5781218	-3.6892580
H	1.1183913	9.9640762	-4.9333472
H	2.2453090	7.7504153	-4.6295174
H	-1.5401311	5.7863354	-4.9521792
H	-1.3637750	10.0542135	-5.2375199
H	-3.3054961	5.9992179	-5.4724668
H	-0.4908645	4.2336326	-5.2049440
H	0.6683675	2.1833601	-5.4314818
H	2.1420882	2.9582471	-4.8016561
H	-5.5803091	7.8854644	-5.6515300
H	-5.6076251	6.2438100	-6.3265011
H	-1.0270607	1.2880438	-3.9026608
H	-1.6828544	0.6463743	-1.6084013
H	1.7040311	2.7622938	-0.0439683
H	2.3655456	3.4052876	-2.3509569
H	-4.6960749	7.7831905	-3.0120939
H	-5.4656676	6.8922727	-0.8305827
H	-7.7396275	3.8686083	-2.8563271
H	-6.9416433	4.7394754	-5.0437024
H	-0.0906761	2.0623145	1.4584937

H	-0.0230650	0.3385102	1.0317184
H	-8.1320760	4.4784155	-0.3676365
H	-6.8682662	5.4349554	0.4417947
H	-2.4364762	2.0311367	0.8092873
H	-5.3773975	3.5475171	0.1852190
H	-4.1944442	2.0393812	0.0810546
H	-6.5929532	-2.2430918	0.7590845
H	-7.8516397	-0.1856571	0.0929897
H	-4.1143917	-2.1213394	1.0830014
H	-1.4829914	4.3201166	-2.7534835
H	-4.4954966	3.9078050	-2.3808509
H	1.2491128	5.1354988	0.6232657
H	1.9625014	4.9981080	2.9780630
H	-2.1432590	5.2277703	4.2313859
H	-2.8575855	5.3840776	1.8723435
H	-4.2365448	-0.1162453	-5.5957691
H	-4.3854196	-0.8027102	-7.9665990
H	-4.7606067	3.3197213	-9.1316835
H	-4.6310062	4.0016415	-6.7556942
H	-0.9180366	6.5501810	-1.9924864
H	0.1799506	6.8345258	-0.6375664
H	0.3976060	5.3969804	-1.6709641
H	-5.9660066	1.0590715	-4.2513974
H	-4.5871258	0.8437549	-3.1436371
H	-5.6738905	2.2437507	-2.9729807
H	-3.3273629	2.6189303	-2.1956301
H	-2.7061167	5.5771042	-2.6532256

Table 10: Cartesian coordinates and total energy in a.u. of complex **3f@1**

E(RI-TPSS-D3/def2-TZVP)=-3030.504804719

N	-6.0508616	2.0331519	1.4320260
C	-7.1097832	3.0121625	1.6474866
C	-7.4747399	3.7400594	0.3647750
C	-7.7097168	5.1182914	0.3648749
C	-8.0183129	5.7933466	-0.8148732
C	-8.0915334	5.0998574	-2.0292256
C	-7.8809542	3.7172096	-2.0260861
C	-7.5798544	3.0439634	-0.8444578
C	-8.3275187	5.8471430	-3.3240051
N	-7.1083795	6.5017472	-3.8104979
C	-6.8062289	7.7884239	-3.4639445
O	-7.5654042	8.4784230	-2.7744703
C	-5.5113434	8.3384611	-3.9903939
C	-4.4845220	7.5255934	-4.4801552
C	-3.2944150	8.0817075	-4.9590248
C	-3.1334057	9.4726712	-4.9365812
C	-4.1512055	10.2881595	-4.4455466
C	-5.3341883	9.7271853	-3.9676732
C	-2.1415653	7.2491848	-5.4510127
O	-1.0209591	7.7435553	-5.6069917
N	-2.4051289	5.9322272	-5.7018447
C	-1.3306033	4.9736484	-5.9361935
C	-0.9808584	4.2045212	-4.6747994
C	-0.8155972	2.8166906	-4.7004951
C	-0.5197455	2.1088268	-3.5366153
C	-0.3928209	2.7784084	-2.3135806

C	-0.5386196	4.1694717	-2.2909027
C	-0.8245045	4.8757605	-3.4566408
C	-0.1655898	1.9996630	-1.0361374
N	-1.3970726	1.3729048	-0.5454285
C	-1.7263464	0.0897981	-0.8803174
O	-0.9911299	-0.6167992	-1.5788991
C	-3.0181059	-0.4364522	-0.3221350
C	-4.0226416	0.3957287	0.1815183
C	-5.2053607	-0.1389038	0.7014657
C	-5.3825516	-1.5281290	0.7049725
C	-4.3883286	-2.3626891	0.1980031
C	-3.2121703	-1.8230373	-0.3199700
C	-6.3360284	0.7152439	1.2082481
O	-7.4590146	0.2381385	1.3958501
H	-4.0208706	11.3666497	-4.4322916
H	-2.1982444	9.8871582	-5.2999478
H	-4.5947484	6.4463882	-4.4438226
H	-6.1354479	10.3430925	-3.5712804
H	-6.4202299	5.9221859	-4.2803243
H	-3.3119679	5.5345767	-5.4724357
H	-1.6365682	4.2797875	-6.7256408
H	-0.4698137	5.5499218	-6.2893582
H	-9.0718827	6.6349557	-3.1869935
H	-8.6741820	5.1623097	-4.1038251
H	-0.9286328	2.2857039	-5.6425732
H	-0.4000028	1.0286686	-3.5607845
H	-0.4302725	4.7048763	-1.3495394
H	-0.9258936	5.9572390	-3.4246947
H	-8.1866601	6.8670519	-0.8102918
H	-7.6397888	5.6686225	1.2998913

H	-7.4266772	1.9684771	-0.8592594
H	-7.9484567	3.1619252	-2.9598249
H	0.2127621	2.6589640	-0.2487753
H	0.5518417	1.1916425	-1.1969740
H	-7.9715398	2.4614869	2.0375655
H	-6.7817560	3.7320975	2.4045234
H	-2.0698007	1.9676609	-0.0722301
H	-5.1426600	2.4126725	1.1792137
H	-3.9012977	1.4729191	0.1239350
H	-4.5313727	-3.4396356	0.2044013
H	-6.3119135	-1.9263011	1.1001397
H	-2.4287431	-2.4544630	-0.7274449
C	-3.3843154	1.0216966	-6.5794228
C	-4.3245432	1.8681272	-5.9861394
C	-5.2242379	2.5565538	-6.8088021
C	-5.1547711	2.4060411	-8.1892066
C	-4.2085744	1.5726104	-8.8070739
C	-3.3323206	0.8792838	-7.9659709
N	-4.3460444	1.9979052	-4.5588287
C	-4.0309633	0.7957948	-3.7627878
C	-4.4669848	3.2214340	-3.9529698
O	-4.7392133	4.2559818	-4.5874528
C	-4.2173304	3.2493552	-2.4544676
C	-4.2343136	4.6707418	-1.9139599
C	-4.0010402	4.6901585	-0.4117947
C	-4.4825137	6.3465927	5.9878978
C	-4.1727759	1.4500208	-10.3351737
N	-4.1541546	5.9032965	0.2082734
C	-4.2070526	6.0135450	1.6362177
C	-3.3341867	5.3052495	2.4718655

C	-3.4420425	5.4304507	3.8522865
C	-4.4004126	6.2575540	4.4593300
C	-4.4816336	7.1089566	-0.5768743
C	-5.2453076	6.9751949	3.6067121
C	-5.1548120	6.8590381	2.2196667
C	-3.0614260	0.5022746	-10.8194473
C	-5.5305958	0.9042291	-10.8360132
C	-3.9239311	2.8461046	-10.9528345
C	-5.6006511	7.2929789	6.4589565
C	-4.7621781	4.9389312	6.5651036
C	-3.1365101	6.8679842	6.5436685
O	-3.7161337	3.6531400	0.2126338
H	-5.8415176	7.4229232	1.5967909
H	-6.0016476	7.6380969	4.0136210
H	-2.7496220	4.8651714	4.4706229
H	-2.5800755	4.6588065	2.0437987
H	-2.6761523	0.4755537	-5.9643306
H	-2.5821257	0.2151503	-8.3820222
H	-5.8681720	2.9547283	-8.7985333
H	-5.9696155	3.2067125	-6.3702907
H	-3.9079839	7.1190399	-1.5032030
H	-5.5480452	7.1624140	-0.8190556
H	-4.1996144	7.9837976	0.0096786
H	-4.2917587	-0.0815104	-4.3547687
H	-2.9709809	0.7492298	-3.4934045
H	-4.6280887	0.7835545	-2.8508026
H	-3.2541281	2.7730367	-2.2410932
H	-5.1940378	5.1491745	-2.1367728
H	-3.0759944	0.4530449	-11.9133519
H	-2.0695190	0.8533786	-10.5144260

H	-3.2020891	-0.5152288	-10.4382767
H	-3.9043145	2.7759760	-12.0464937
H	-4.7095391	3.5556515	-10.6742510
H	-2.9648112	3.2534073	-10.6156385
H	-5.5220369	0.8185944	-11.9286594
H	-5.7299568	-0.0863853	-10.4133602
H	-6.3570568	1.5650307	-10.5557789
H	-4.8166397	4.9844981	7.6589379
H	-5.7128376	4.5474571	6.1878173
H	-3.9733207	4.2293501	6.2958490
H	-3.1784255	6.9268037	7.6373440
H	-2.3072038	6.2067517	6.2729674
H	-2.9159901	7.8667234	6.1520662
H	-5.6192018	7.3180976	7.5536523
H	-5.4404458	8.3173641	6.1048037
H	-6.5854652	6.9570820	6.1162260
H	-3.4654290	5.2665294	-2.4184568
H	-4.9775596	2.6515702	-1.9398839

Table 11: Cartesian coordinates and total energy in a.u. of complex **3g@1**

E(RI-TPSS-D3/def2-TZVP)=-2866.339344806

C	-4.7930534	1.2078977	0.2908928
C	-4.0812937	0.0569663	0.6441037
C	-4.7715839	-1.1512403	0.8038085
C	-6.1506924	-1.2015047	0.6133527
C	-6.8538748	-0.0517840	0.2585902

C	-6.1788935	1.1652585	0.1042040
C	-2.5846408	0.0307986	0.8018295
O	-1.9681049	-1.0378446	0.8238407
C	-6.9982268	2.3714504	-0.2586154
O	-8.1545998	2.2600701	-0.6787882
N	-1.9571340	1.2417326	0.9039762
C	-0.5128995	1.3516843	0.7559196
C	-0.1028570	1.7218929	-0.6609090
C	-0.8667971	1.3267927	-1.7635354
C	-0.4877353	1.6823453	-3.0559345
C	0.6657425	2.4404873	-3.2799410
C	1.4475390	2.8145738	-2.1798332
C	1.0645317	2.4576914	-0.8884759
C	1.0178334	2.9207412	-4.6715294
N	0.4411653	4.2425979	-4.9426903
C	1.0974061	5.3856944	-4.5869610
O	2.2452075	5.3708922	-4.1276420
C	0.3581265	6.6774061	-4.7970641
C	-1.0231070	6.7304606	-5.0029047
C	-1.6820841	7.9528646	-5.1636255
C	-0.9391624	9.1392775	-5.1152854
C	0.4390042	9.0939153	-4.9103421
C	1.0877990	7.8712132	-4.7446723
C	-3.1712247	8.0601930	-5.3466843
O	-3.7460081	9.1514345	-5.2942794
N	-3.8422326	6.8881886	-5.5712696
C	-5.3018864	6.8328294	-5.5524678
C	-5.8237029	6.3332364	-4.2193222
C	-5.4339936	6.9779722	-3.0390549
C	-5.8696413	6.5105513	-1.8025415

C	-6.7131576	5.3980569	-1.7143803
C	-7.1241298	4.7671366	-2.8950932
C	-6.6749661	5.2269797	-4.1329717
C	-7.1162699	4.8362639	-0.3688328
N	-6.4096924	3.5901429	-0.0627556
O	-3.0614134	4.0103334	-0.0332883
C	-2.3100035	4.6629534	-0.7805256
C	-2.5025155	4.6635271	-2.2905564
C	-3.5625337	3.6518613	-2.6969714
C	-3.5412528	3.3624850	-4.1850516
O	-2.8160257	3.9918393	-4.9779334
N	-1.2443243	5.3808567	-0.3067151
C	-0.3751631	6.1315106	-1.2337545
C	-0.8009513	5.2538056	1.0507425
C	-1.6966182	5.2150043	2.1250558
C	-1.2245339	5.0625423	3.4264708
C	0.1470657	4.9591923	3.6755431
C	1.0477755	5.0202699	2.6098266
C	0.5732127	5.1639314	1.3114612
O	0.6702199	4.8047725	4.9349979
N	-4.3521729	2.3430427	-4.5994807
C	-4.3275310	1.8913523	-5.9612851
C	-4.4951771	2.7820681	-7.0229407
C	-4.4798960	2.3179752	-8.3352594
C	-4.3059894	0.9546313	-8.5967417
C	-4.1442436	0.0581805	-7.5373753
C	-4.1488362	0.5300047	-6.2294494
O	-4.2866536	0.4355339	-9.8669868
C	-5.1854751	1.5906288	-3.6419856
H	1.0101783	10.0173929	-4.8754388

H	2.1576998	7.8145322	-4.5700015
H	-1.5956138	5.8095671	-4.9894990
H	-1.4655293	10.0812993	-5.2335196
H	-3.3576596	5.9982924	-5.4830265
H	-0.5410742	4.2708176	-5.1975011
H	0.6335344	2.2308820	-5.4285901
H	2.0998281	3.0124850	-4.7894014
H	-5.6515360	7.8505368	-5.7495898
H	-5.6486436	6.1840981	-6.3639428
H	-1.1010704	1.3769696	-3.9016504
H	-1.7646995	0.7357364	-1.6092710
H	1.6769641	2.7582389	-0.0417696
H	2.3424099	3.4077752	-2.3458098
H	-4.7854930	7.8485487	-3.0972329
H	-5.5486499	7.0127622	-0.8922822
H	-7.7828278	3.9044337	-2.8321461
H	-6.9861050	4.7166955	-5.0423710
H	-0.1357905	2.1015493	1.4588897
H	-0.0949577	0.3776670	1.0298385
H	-8.1822467	4.5966064	-0.3487759
H	-6.9000829	5.5584661	0.4248976
H	-2.4830112	2.1052423	0.7984521
H	-5.4236765	3.6537448	0.1696793
H	-4.2573851	2.1361401	0.1167224
H	-6.6805459	-2.1415210	0.7399292
H	-7.9270952	-0.0691337	0.0970554
H	-4.2012143	-2.0368849	1.0658662
H	-1.5495182	4.4184379	-2.7720777
H	-4.5591076	4.0034417	-2.4106718
H	1.2845345	5.1907572	0.4933003

H	2.1122458	4.9469560	2.8080061
H	-1.9323790	5.0308083	4.2522872
H	-2.7601004	5.2969270	1.9468219
H	-4.0073409	-0.1689986	-5.4100226
H	-4.0071252	-0.9968872	-7.7507502
H	-4.6096868	3.0191193	-9.1572210
H	-4.6308975	3.8381491	-6.8240376
H	-0.9767145	6.6189304	-2.0002632
H	0.1442584	6.9026764	-0.6643385
H	0.3604142	5.4806786	-1.7191127
H	-5.9192533	1.0191794	-4.2098445
H	-4.5895710	0.9039259	-3.0301009
H	-5.7187840	2.2792904	-2.9843157
H	-3.4028307	2.7186511	-2.1472448
H	-2.7789010	5.6658130	-2.6377628
H	-0.0552685	4.7724650	5.5792179
H	-4.4016604	1.1559823	-10.5074296

Table 12: Cartesian coordinates and total energy in a.u. of complex **3h@1**

E(RI-TPSS-D3/def2-TZVP)=-2826.590231228

C	-4.8171303	1.2018557	0.3166197
C	-4.4609504	-0.1248038	0.0561727
C	-5.4155093	-1.1353957	0.2223765
C	-6.6989772	-0.8178666	0.6639065
C	-7.0441111	0.5052079	0.9328374
C	-6.1015504	1.5266650	0.7625249

C	-3.0852646	-0.5254773	-0.3954318
O	-2.8684789	-1.6332117	-0.8995249
C	-6.5416038	2.9432122	1.0163108
O	-7.7384370	3.2320023	1.1093739
N	-2.0884437	0.3836884	-0.1798505
C	-0.7011138	0.1036703	-0.5611348
C	-0.3627514	0.6439049	-1.9337857
C	-0.8035040	-0.0218890	-3.0839257
C	-0.5643066	0.5185878	-4.3465639
C	0.1262420	1.7250671	-4.4927925
C	0.5914430	2.3743359	-3.3436366
C	0.3458038	1.8410113	-2.0807394
C	0.3614389	2.3324781	-5.8639785
N	0.0429619	3.7560340	-5.8808009
C	1.0150632	4.7062757	-5.7445451
O	2.2194738	4.4397293	-5.8044718
C	0.5422983	6.1150875	-5.5081537
C	-0.7610921	6.4186779	-5.1043895
C	-1.1487682	7.7398933	-4.8621362
C	-0.2084854	8.7670880	-5.0069594
C	1.0933571	8.4708411	-5.4071509
C	1.4709686	7.1526223	-5.6556663
C	-2.5414454	8.1165484	-4.4437536
O	-2.7905806	9.2245721	-3.9554735
N	-3.5150517	7.1837592	-4.6645030
C	-4.9094724	7.4305234	-4.2842624
C	-5.2217329	6.9161294	-2.8954208
C	-4.8119613	7.6350711	-1.7656759
C	-5.0191092	7.1180513	-0.4879265
C	-5.6434501	5.8807196	-0.3046320

C	-6.0806724	5.1791830	-1.4333333
C	-5.8696873	5.6905019	-2.7117305
C	-5.8395230	5.3042476	1.0871088
N	-5.5485788	3.8760545	1.1248350
O	-2.6621142	3.6168461	0.0383647
C	-2.2424914	4.5134871	-0.7173442
C	-2.3631215	4.3974151	-2.2282406
C	-3.1669774	3.1688313	-2.6250408
C	-3.3041638	3.0581151	-4.1343488
O	-2.8725540	3.9448928	-4.8951163
N	-1.6748481	5.6639298	-0.2422896
C	-1.1805100	6.6965696	-1.1721282
C	-1.7117288	5.9935432	1.1536917
C	-1.4306554	5.0500475	2.1488529
C	-1.5039106	5.3994530	3.4917419
C	-1.8483041	6.7028856	3.8875724
C	-2.1020292	7.6511935	2.8839539
C	-2.0370644	7.2987163	1.5407079
N	-1.9975257	7.0278034	5.2367671
N	-3.9046429	1.9218438	-4.6026459
C	-3.8994809	1.5973832	-6.0004780
C	-4.2012240	2.5464829	-6.9836565
C	-4.1665222	2.2018468	-8.3293227
C	-3.8408339	0.8975458	-8.7385486
C	-3.5623423	-0.0554045	-7.7458798
C	-3.5885284	0.2927043	-6.4001340
N	-3.7328894	0.5761533	-10.0925889
C	-4.4127239	0.9004594	-3.6676103
H	1.8177158	9.2719781	-5.5251851
H	2.4824382	6.8983052	-5.9571179

H	-1.4694711	5.6140314	-4.9342227
H	-0.5218926	9.7860321	-4.8019151
H	-3.2694368	6.2448758	-4.9634929
H	-0.9348732	3.9935141	-5.7349332
H	-0.2516445	1.8198362	-6.6122628
H	1.4138183	2.2450243	-6.1514364
H	-5.0617901	8.5111065	-4.3405774
H	-5.5504732	6.9428461	-5.0252260
H	-0.9239516	0.0022245	-5.2331860
H	-1.3477454	-0.9567124	-2.9756903
H	0.7050767	2.3636693	-1.1960236
H	1.1498570	3.3014932	-3.4418599
H	-4.3135298	8.5914100	-1.9017621
H	-4.6842993	7.6770875	0.3822434
H	-6.5904429	4.2276351	-1.3093349
H	-6.2064546	5.1261689	-3.5795957
H	-0.0478299	0.5496896	0.1954310
H	-0.5846320	-0.9825104	-0.5332744
H	-6.8786685	5.4210375	1.4107506
H	-5.1892556	5.8207164	1.8008682
H	-2.3095003	1.3260549	0.1273668
H	-4.5798863	3.6175431	0.9564303
H	-4.0997147	1.9941112	0.1274202
H	-7.4341896	-1.6063636	0.7988414
H	-8.0409988	0.7758864	1.2668332
H	-5.1273187	-2.1587682	0.0026372
H	-1.3659383	4.3469479	-2.6793999
H	-4.1591909	3.2146519	-2.1631537
H	-2.2514624	8.0520297	0.7895135
H	-2.3638192	8.6709309	3.1564227

H	-1.2888901	4.6480802	4.2481365
H	-1.1603021	4.0395493	1.8727719
H	-3.3570736	-0.4636973	-5.6567148
H	-3.3122406	-1.0750262	-8.0293435
H	-4.3975818	2.9568817	-9.0772656
H	-4.4576357	3.5575751	-6.6957822
H	-0.6516094	6.2315604	-2.0045304
H	-1.9945485	7.3117612	-1.5701875
H	-0.4785383	7.3318671	-0.6312949
H	-5.1088828	0.2608113	-4.2103638
H	-3.6056198	0.2876986	-3.2527798
H	-4.9511578	1.3754123	-2.8464627
H	-2.6894511	2.2617651	-2.2392557
H	-2.8366285	5.3024427	-2.6235703
H	-1.4920747	6.4377449	5.8864486
H	-1.9035379	8.0105471	5.4626648
H	-3.8373259	-0.4055972	-10.3182790
H	-4.2555596	1.1697877	-10.7251607

Table 13: Cartesian coordinates and total energy in a.u. of complex **3a′@1**

E(RI-TPSS-D3/def2-TZVP)=-3125.071518298

C	-4.7570835	0.5814623	-0.1830660
C	-4.0682767	0.0474997	0.9121861
C	-4.7163917	-0.8409981	1.7703916
C	-6.0362304	-1.1769772	1.5053455
C	-6.7447067	-0.6628257	0.4220065

C	-6.0871520	0.2300723	-0.4195275
N	-2.7206150	0.4369019	1.1714494
C	-2.3862734	1.8739570	1.1043474
H	-7.7741673	-0.9583962	0.2605654
C	-1.7589969	-0.5100920	1.4333154
O	-2.0177047	-1.7237298	1.4428478
C	-0.3680107	0.0331504	1.6825322
C	0.6582944	-1.0737004	1.8612190
C	2.0647881	-0.4974967	1.8625859
N	3.0970573	-1.4106665	1.8692423
C	4.4421339	-0.9981622	1.6404080
C	4.9528097	0.1711561	2.2146822
C	6.2643979	0.5303366	1.9314658
C	7.1041495	-0.2243983	1.1177112
C	6.5901653	-1.3979921	0.5742088
C	5.2738513	-1.7828469	0.8270703
H	8.1184065	0.1058972	0.9310568
O	2.2622222	0.7252897	1.8235867
C	2.8204345	-2.8608046	1.8865125
N	2.7734236	2.0583046	-0.9156083
C	3.2945309	0.9530122	-1.7071221
C	2.3406519	-0.2298385	-1.7478111
C	2.8326576	-1.5379290	-1.7993746
C	1.9677537	-2.6303486	-1.8322118
C	0.5805350	-2.4369045	-1.8074850
C	0.0875359	-1.1286362	-1.7775922
C	0.9551344	-0.0389226	-1.7510083
C	-0.3582597	-3.6228517	-1.7370297
N	-0.5104403	-4.1093069	-0.3609656
C	0.3611688	-5.0181737	0.1662097

O	1.2512997	-5.5437498	-0.5123437
C	0.1847180	-5.3584468	1.6178520
C	-0.5344482	-4.5495524	2.5030721
C	-0.6344695	-4.8801821	3.8580287
C	-0.0030035	-6.0389061	4.3266873
C	0.7106834	-6.8523510	3.4484028
C	0.8135986	-6.5125293	2.1008289
C	-1.3455440	-4.0144160	4.8618433
O	-1.1733814	-4.1702402	6.0739225
N	-2.1741212	-3.0542891	4.3542986
C	-2.7729078	-2.0289948	5.2054945
C	-1.9834433	-0.7339866	5.1569545
C	-2.5912815	0.4851607	4.8408792
C	-1.8491029	1.6665572	4.8050604
C	-0.4747623	1.6484360	5.0733257
C	0.1330725	0.4279395	5.3901862
C	-0.6106093	-0.7478808	5.4358296
C	0.3430956	2.9189299	4.9753896
N	0.6132187	3.3016462	3.5862081
C	-0.1710221	4.2048378	2.9269143
O	-1.1450952	4.7448608	3.4618424
C	0.2242057	4.5248551	1.5137390
C	1.0037361	3.6684691	0.7287165
C	1.3321119	4.0059608	-0.5887907
C	0.8717553	5.2181191	-1.1186445
C	0.0956042	6.0747911	-0.3409813
C	-0.2377971	5.7281576	0.9669706
C	2.1002688	3.0927902	-1.5054256
O	2.0995174	3.2702243	-2.7262122
H	1.1941431	-7.7524334	3.8174279

H	-0.0794559	-6.2735517	5.3836707
H	-0.9747894	-3.6251617	2.1424606
H	1.3797819	-7.1218580	1.4032179
H	-1.1640334	-3.6166257	0.2379185
H	-2.2179074	-2.9002440	3.3527715
H	-3.8101845	-1.8685848	4.8963185
H	-2.7757934	-2.4364633	6.2207930
H	0.0253171	-4.4566595	-2.3295538
H	-1.3517837	-3.3518217	-2.1053884
H	-3.6582951	0.5135516	4.6301347
H	-2.3277888	2.6121296	4.5631359
H	1.1983608	0.4012387	5.6108403
H	-0.1317724	-1.6866440	5.7026284
H	2.3577043	-3.6442408	-1.8560506
H	3.9078834	-1.7012651	-1.8131657
H	0.5532751	0.9700937	-1.7363774
H	-0.9880638	-0.9617435	-1.7686638
H	1.3035373	2.7949926	5.4843576
H	-0.1912881	3.7574105	5.4288045
H	3.4607997	1.3421007	-2.7168029
H	4.2590820	0.6387604	-1.2944196
H	1.3731680	2.8381279	3.1027741
H	2.6840708	1.8904481	0.0818952
H	1.3115079	2.7079121	1.1323157
H	-0.2553909	7.0147166	-0.7572554
H	1.1255360	5.4578309	-2.1463894
H	-0.8550143	6.3724431	1.5853159
H	0.4706596	-1.6153115	2.7956331
H	-0.0743673	0.6751688	0.8456210
H	4.8888923	-2.6902557	0.3750236

H	7.2140320	-2.0171137	-0.0628359
N	6.7917512	1.7790425	2.5429035
H	4.3585553	0.7924883	2.8662916
H	-4.2508126	1.2677806	-0.8551716
H	-6.6092415	0.6511383	-1.2728338
N	-6.7184091	-2.1089565	2.4389511
H	-4.2158514	-1.2571303	2.6310943
H	1.9868164	-3.0715056	2.5551567
H	2.5823123	-3.2422706	0.8884611
H	3.7018360	-3.3741850	2.2728324
H	-3.3151017	2.4428215	1.1109510
H	-1.8080603	2.1626433	1.9834387
H	-1.8173002	2.1189879	0.2014949
H	-0.3895325	0.6784523	2.5680008
H	0.5714527	-1.8068795	1.0514209
O	7.9647313	2.0758284	2.3049165
O	6.0251343	2.4401460	3.2487462
O	-7.8803467	-2.4264868	2.1789315
O	-6.0799197	-2.5044293	3.4197368

Table 14: Cartesian coordinates and total energy in a.u. of complex **3g′@1**

E(RI-TPSS-D3/def2-TZVP)=-2866.341199704

C	-5.0393184	0.8204586	0.2009420
C	-4.2119772	-0.1896238	0.7065139
C	-4.6811929	-1.5060494	0.7404036
C	-5.9527545	-1.8271938	0.2564955

C	-6.7671844	-0.8087841	-0.2537791
C	-6.3090480	0.5070274	-0.2811005
C	-2.8492339	0.2004461	1.2025566
O	-2.5711966	1.3753939	1.4669842
C	-6.5151445	-3.2213050	0.3047554
O	-7.7227869	-3.4281941	0.1516832
N	-1.9340951	-0.8072251	1.3133940
C	-0.5772964	-0.5581358	1.8085279
C	-0.4686957	-0.7793682	3.3019564
C	-0.9921330	0.1641779	4.1949439
C	-0.9683383	-0.0759950	5.5677873
C	-0.4128109	-1.2512500	6.0817858
C	0.1332696	-2.1812275	5.1898629
C	0.1003045	-1.9487335	3.8169310
C	-0.4006968	-1.5229003	7.5751127
N	-0.8431668	-2.8787421	7.8809061
C	0.0505251	-3.8889359	8.1004857
O	1.2594763	-3.6861978	8.2493010
C	-0.5159051	-5.2814652	8.1475938
C	-1.7926317	-5.5965937	7.6735151
C	-2.2657766	-6.9116149	7.7057310
C	-1.4376177	-7.9263759	8.2004833
C	-0.1628625	-7.6189735	8.6729825
C	0.2994912	-6.3045879	8.6467445
C	-3.6326857	-7.2955284	7.2164255
O	-3.9164573	-8.4690627	6.9518208
N	-4.5442409	-6.2841494	7.1099819
C	-5.9019412	-6.5268020	6.6137530
C	-6.0063981	-6.3102051	5.1192844
C	-5.4938269	-7.2636574	4.2304987

C	-5.5126716	-7.0283788	2.8567996
C	-6.0518173	-5.8478309	2.3374588
C	-6.5873214	-4.9078068	3.2251273
C	-6.5597057	-5.1357054	4.5990112
C	-6.0587090	-5.5833977	0.8426079
N	-5.6192386	-4.2281504	0.5309382
O	-2.8035957	-3.9903461	1.8204256
C	-2.5597675	-4.6947069	2.8146249
N	-2.0204347	-5.9520869	2.7109854
C	-1.7625260	-6.7634091	3.9169973
C	-2.8734190	-4.2188452	4.2227368
C	-3.5868397	-2.8760948	4.2049510
C	-3.9087500	-2.4021428	5.6113809
O	-3.6626591	-3.1022864	6.6081279
N	-4.4610124	-1.1502981	5.7103367
C	-4.7182842	-0.3423609	4.5018095
C	-4.6486293	-0.5181585	6.9823062
C	-4.2712859	0.8231858	7.1366323
C	-4.4539306	1.4475424	8.3676203
C	-4.9999302	0.7566720	9.4472671
C	-5.3794319	-0.5784375	9.2779306
C	-5.2115974	-1.2183021	8.0492323
O	-5.9375770	-1.3260630	10.2853163
C	-1.8520490	-6.5884288	1.4385715
C	-1.3081512	-5.8925116	0.3587265
C	-1.1606614	-6.5376348	-0.8698320
C	-1.5411539	-7.8738595	-1.0272735
C	-2.0667228	-8.5607642	0.0649402
C	-2.2289285	-7.9314756	1.2962467
O	-0.6210524	-5.7932858	-1.8897380

H	-5.1339128	1.2487833	10.4081257
H	-1.4232346	-8.3698740	-1.9882204
H	0.4737455	-8.4081576	9.0629664
H	1.2929438	-6.0442963	8.9985151
H	-2.4084157	-4.8188639	7.2327294
H	-1.8148327	-8.9442934	8.2044894
H	-4.2562790	-5.3191829	7.2343956
H	-1.8166544	-3.0870768	7.6768205
H	-1.0504175	-0.8094383	8.0922127
H	0.6133048	-1.4284070	7.9765668
H	-6.1494714	-7.5586119	6.8741821
H	-6.5807451	-5.8541329	7.1468332
H	-1.3911866	0.6554946	6.2523820
H	-1.4287968	1.0776627	3.7995167
H	0.5205505	-2.6858766	3.1351940
H	0.5891946	-3.0887756	5.5764835
H	-5.0689155	-8.1808785	4.6299445
H	-5.0983300	-7.7678731	2.1756281
H	-7.0310850	-3.9958599	2.8348485
H	-6.9717035	-4.3906624	5.2772231
H	0.1051107	-1.2244924	1.2720827
H	-0.3369023	0.4761897	1.5513569
H	-7.0709546	-5.6832414	0.4378802
H	-5.4050993	-6.2978974	0.3318557
H	-2.2178912	-1.7735063	1.1895310
H	-4.6467263	-4.0160565	0.7353225
H	-4.0668225	-2.2798939	1.1900213
H	-6.9448766	1.2925859	-0.6795770
H	-7.7566980	-1.0737799	-0.6130526
H	-4.6653142	1.8395585	0.1958136

H	-1.9460036	-4.1371567	4.8007254
H	-4.5112082	-2.9563316	3.6220417
H	-2.6525753	-8.4754681	2.1328437
H	-2.3620754	-9.5998496	-0.0477414
H	-1.0019289	-4.8598932	0.4535859
H	-3.8322474	1.3693344	6.3092688
H	-4.1588196	2.4855692	8.4902447
H	-5.5168447	-2.2503896	7.9439727
H	-1.3043792	-6.1476686	4.6915346
H	-2.6807116	-7.2078001	4.3142374
H	-1.0625671	-7.5549740	3.6503772
H	-5.3998097	0.4639479	4.7714189
H	-3.7968531	0.0824891	4.0912875
H	-5.1973475	-0.9544899	3.7365597
H	-2.9686300	-2.1240551	3.7021670
H	-3.4896517	-4.9694046	4.7300033
H	-0.5556935	-6.3464725	-2.6847962
H	-6.0152782	-0.7762314	11.0815921

0.3 Gas phase results on the structures with the C=C axle

Table 15: Cartesian coordinates and total energy in a.u. of complex **2a@1**

E(RI-TPSS-D3/def2-TZVP)=-3123.834770161

C	-5.1227283	-1.1830483	0.4686801
C	-4.2667596	-0.4025756	-0.3208438
C	-4.7702363	0.7108669	-1.0079034

C	-6.1156103	1.0373855	-0.9127898
C	-6.9488278	0.2456090	-0.1252714
C	-6.4712821	-0.8621956	0.5681030
N	-2.8940781	-0.7660450	-0.4423681
C	-1.9100978	0.1858325	-0.2889497
C	-0.5155016	-0.2931380	-0.4372182
C	0.4941898	0.3862512	0.1183340
C	1.8843895	-0.1247594	0.0257348
O	2.1192469	-1.3125280	-0.2561815
N	-8.3841173	0.5907820	-0.0259262
O	-9.0987157	-0.1353812	0.6713420
O	-2.1702414	1.3708362	-0.0179345
N	2.8814948	0.7809850	0.3193981
C	4.2280101	0.3637695	0.5185742
C	4.8364138	-0.5941935	-0.3075199
C	6.1573195	-0.9628847	-0.0845863
C	6.8623391	-0.3730176	0.9607986
C	6.2817389	0.5837051	1.7885317
C	4.9621209	0.9509051	1.5624317
N	8.2692930	-0.7648631	1.1926377
O	8.7544752	-1.6191484	0.4451729
O	8.8703215	-0.2115336	2.1186275
O	-8.7759903	1.5829201	-0.6470947
N	0.8694179	-3.1873364	-2.6567444
C	0.9633231	-2.4605087	-3.9257657
C	0.1537836	-1.1813297	-3.9131112
C	0.7782192	0.0665859	-3.8170630
C	0.0270609	1.2371715	-3.7664399
C	-1.3714857	1.1862805	-3.8027627
C	-1.9967117	-0.0607963	-3.8943119

C	-1.2447101	-1.2340884	-3.9571147
C	-2.1739618	2.4710826	-3.7383398
N	-1.7979878	3.2838713	-2.5844755
C	-0.9511436	4.3523110	-2.7149021
O	-0.6275576	4.8098792	-3.8133778
C	-0.0229006	-4.2081264	-2.4802018
O	-0.7855095	-4.5787457	-3.3784406
C	-0.0137576	-4.8720991	-1.1336172
C	-0.5835467	-6.1466036	-1.0276319
C	-0.5982367	-6.8053477	0.2006546
C	-0.0599532	-6.1959219	1.3322922
C	0.5069913	-4.9186205	1.2413190
C	0.5203522	-4.2633298	0.0064898
C	1.0263860	-4.2873590	2.5031519
N	1.8670676	-3.2202327	2.3432715
C	2.2451349	-2.3734351	3.4686595
C	1.3938573	-1.1191626	3.5495793
C	0.0064458	-1.1913605	3.3806686
C	-0.7759496	-0.0418550	3.4510296
C	-0.1951573	1.2064121	3.6972030
C	1.1911052	1.2788249	3.8835488
C	1.9736672	0.1273845	3.8035166
C	-1.0348722	2.4662482	3.7111061
N	-0.9257945	3.2153066	2.4543405
C	0.0145687	4.1906339	2.2864835
O	0.7972040	4.5125684	3.1876760
O	0.7003447	-4.7153267	3.6131069
C	-0.4280674	4.9481385	-1.4374968
C	-0.5002649	4.2919457	-0.2046589
C	0.0449920	4.8680241	0.9468469

C	0.6787649	6.1132981	0.8570471
C	0.7464867	6.7760416	-0.3670750
C	0.2011769	6.1971641	-1.5112915
H	-1.0333677	-7.7977479	0.2760283
H	-1.0073422	-6.5984482	-1.9190838
H	0.9060031	-3.2496248	-0.0586399
H	-0.0747047	-6.6844922	2.3016027
H	2.0444212	-2.8492950	1.4157520
H	1.4398758	-2.8620616	-1.8839100
H	2.0186529	-2.2423784	-4.1161977
H	0.5987063	-3.1407171	-4.6998821
H	2.1179397	-2.9821704	4.3693154
H	3.3031155	-2.1064270	3.3775813
H	1.8647194	0.1217185	-3.7883009
H	0.5240878	2.2020133	-3.7053082
H	-3.0831413	-0.1161317	-3.9274373
H	-1.7347768	-2.2007205	-4.0434312
H	-0.4608034	-2.1552534	3.1991061
H	-1.8533835	-0.1132729	3.3135531
H	1.6456566	2.2457537	4.0831236
H	3.0502369	0.1951486	3.9459274
H	-3.2462936	2.2506632	-3.6981694
H	-1.9865974	3.0905959	-4.6202954
H	-0.7081520	3.1392099	4.5076968
H	-2.0900617	2.2219837	3.8636495
H	-1.9464159	2.8707326	-1.6690070
H	-1.5151634	2.9264838	1.6814437
H	-0.9427625	3.3013744	-0.1453151
H	1.2305963	7.7463283	-0.4301439
H	1.1108828	6.5387281	1.7574648

H	0.2578995	6.6876853	-2.4779399
C	-2.5618669	-2.1976648	-0.6218190
H	-0.3067984	-1.1948395	-1.0015353
H	0.2819904	1.2798082	0.6946882
C	2.5722464	2.2124030	0.5416461
H	-4.7320741	-2.0328808	1.0193316
H	-7.1498018	-1.4474726	1.1772774
H	-6.5303978	1.8855059	-1.4445378
H	-4.1086968	1.3076343	-1.6212387
H	4.4953571	1.6846355	2.2106155
H	6.8598928	1.0189975	2.5949451
H	6.6509544	-1.6917378	-0.7164039
H	4.2805942	-1.0382676	-1.1208079
H	-3.4902670	-2.7474794	-0.7669714
H	-1.9545024	-2.3285390	-1.5198943
H	-2.0325358	-2.6006275	0.2452052
H	3.4851491	2.7851086	0.3788294
H	1.8327815	2.5454013	-0.1868048
H	2.2022449	2.3982474	1.5540799

Table 16: Cartesian coordinates and total energy in a.u. of complex **2b@1**

E(RI-TPSS-D3/def2-TZVP)=-3389.022865141

C	-0.0001890	6.1290970	-1.2404001
C	-0.5576513	4.8487156	-1.1350477
C	-0.5637388	4.2060649	0.1066252
C	-0.0319216	4.8308627	1.2391854

C	0.5284009	6.1082359	1.1185338
C	0.5362487	6.7541041	-0.1165322
C	-1.0749180	4.2024653	-2.3905813
O	-0.7646152	4.6323413	-3.5045839
C	-0.0136283	4.1828641	2.5938270
O	0.7400694	4.5798080	3.4884310
N	-1.8938234	3.1204519	-2.2214464
C	-2.2665003	2.2667809	-3.3421633
C	-1.3948675	1.0266774	-3.4356965
C	-0.0171873	1.1016494	-3.2024706
C	0.7785311	-0.0381530	-3.2854493
C	0.2200749	-1.2796712	-3.6061161
C	-1.1554870	-1.3538346	-3.8588633
C	-1.9505768	-0.2120489	-3.7697522
C	1.0682093	-2.5337333	-3.6157473
N	0.9566873	-3.2774884	-2.3558471
C	0.0029685	-4.2380119	-2.1789123
O	-0.7769791	-4.5659588	-3.0804099
C	-0.0435077	-4.8933053	-0.8283976
C	0.5148899	-4.3101153	0.3128979
C	0.4333423	-4.9442108	1.5564432
C	-0.2224884	-6.1778794	1.6522574
C	-0.7839095	-6.7628857	0.5187640
C	-0.7036691	-6.1230135	-0.7168583
C	0.9795955	-4.3401796	2.8206416
O	0.6729118	-4.7883407	3.9283498
N	1.8283694	-3.2771339	2.6671371
C	2.2327786	-2.4587723	3.8073443
C	1.4139229	-1.1859163	3.9017364
C	0.0157139	-1.2548031	3.9019378

C	-0.7488721	-0.0936262	3.9679006
C	-0.1382765	1.1622505	4.0456455
C	1.2601396	1.2325610	4.0603835
C	2.0251782	0.0689606	3.9795719
C	-0.9627245	2.4317153	4.0588920
N	-0.8878669	3.1487701	2.7828442
O	2.1805565	-1.4122536	0.0968759
C	1.9007699	-0.2341672	0.3836256
N	2.8614256	0.7440094	0.4940562
C	4.2299177	0.4380765	0.2248542
C	4.8481013	-0.6690064	0.8204605
C	6.1865440	-0.9323748	0.5637703
C	6.9187306	-0.0965441	-0.2860081
C	6.3068747	1.0055371	-0.8789123
C	4.9634220	1.2702493	-0.6265304
C	8.3678910	-0.4098819	-0.5406632
F	9.0996282	-0.3835406	0.6086272
C	0.5014945	0.2110427	0.5759916
C	-0.5039403	-0.4568313	-0.0005125
C	-1.8903600	0.0665144	0.0762297
O	-2.1133224	1.2522895	0.3822547
N	-2.8897636	-0.8120815	-0.2747256
C	-4.2119674	-0.3574038	-0.5573176
C	-4.8977927	-0.9263494	-1.6389541
C	-6.1821458	-0.4995088	-1.9618397
C	-6.7937391	0.4969311	-1.2031364
C	-6.1208891	1.0528883	-0.1121828
C	-4.8389969	0.6306478	0.2166194
C	-8.1693445	1.0023425	-1.5403155
F	-8.1404109	2.3020512	-1.9519039

F	-9.0000452	0.9628631	-0.4612592
F	-8.7626952	0.2877430	-2.5287494
F	8.5280688	-1.6562453	-1.0670470
F	8.9473713	0.4634207	-1.4013696
H	-1.2895888	-7.7208937	0.5991523
H	-1.1458436	-6.5545219	-1.6094936
H	0.9768418	-3.3299490	0.2367053
H	-0.2861848	-6.6517451	2.6267993
H	1.9603404	-2.8673756	1.7471925
H	1.5333213	-2.9722366	-1.5794166
H	2.1229738	-2.2839013	-3.7620951
H	0.7496842	-3.2107862	-4.4118827
H	2.0890854	-3.0798112	4.6963819
H	3.2986631	-2.2202198	3.7229927
H	1.8473762	0.0345788	-3.0928304
H	0.4328085	2.0590053	-2.9561852
H	-3.0189369	-0.2814881	-3.9621020
H	-1.5937905	-2.3160635	-4.1101587
H	-0.4706436	-2.2255845	3.8524324
H	-1.8348985	-0.1623174	3.9611858
H	1.7400016	2.2054805	4.1328141
H	3.1114068	0.1379767	3.9829431
H	-3.3183397	1.9796041	-3.2399643
H	-2.1605948	2.8771974	-4.2446686
H	-0.5988148	3.1228504	4.8235140
H	-2.0136395	2.2025239	4.2607345
H	-2.0626063	2.7499835	-1.2917447
H	-1.4440911	2.7958563	2.0114152
H	-0.9414768	3.1902619	0.1831753
H	0.9647655	7.7485125	-0.2033252

H	0.9508433	6.5719530	2.0044878
H	0.0088947	6.6072418	-2.2149946
C	-2.5991206	-2.2454047	-0.5015658
H	-0.2881576	-1.3325547	-0.6024048
H	0.2853069	1.0971814	1.1613443
C	2.5023811	2.1622863	0.7160067
H	-4.4236391	-1.6908932	-2.2442597
H	-6.7021153	-0.9376942	-2.8065842
H	-6.6070220	1.8139232	0.4902019
H	-4.3266944	1.0595445	1.0654431
H	4.4849600	2.1189463	-1.1046618
H	6.8724141	1.6504365	-1.5421034
H	6.6687924	-1.7861636	1.0294331
H	4.2813044	-1.3122285	1.4804448
H	-1.8682992	-2.5919101	0.2290181
H	-3.5215648	-2.8056666	-0.3465690
H	-2.2255187	-2.4333657	-1.5125719
H	3.4217301	2.7218858	0.8820145
H	1.9676948	2.5855218	-0.1387367
H	1.8899520	2.2543273	1.6145057

Table 17: Cartesian coordinates and total energy in a.u. of complex **2c@1**

E(RI-TPSS-D3/def2-TZVP)=-3633.846602797

C	-5.1788402	-0.9637269	0.4661218
C	-4.2660855	-0.2821163	-0.3442614
C	-4.6988953	0.8030762	-1.1119673

C	-6.0301649	1.2034752	-1.0724518
C	-6.9314815	0.5116669	-0.2624006
C	-6.5151638	-0.5730522	0.5071546
N	-2.9025087	-0.7148368	-0.3969144
C	-1.8899400	0.1889802	-0.1924287
C	-0.5131509	-0.3537667	-0.2434966
C	0.5131957	0.3532202	0.2433406
C	1.8900338	-0.1894244	0.1920806
O	2.1141205	-1.3933901	-0.0398762
Cl	-8.6033439	1.0104636	-0.2138232
O	-2.1139235	1.3929153	0.0395855
N	2.9025545	0.7144349	0.3963243
C	4.2661467	0.2817272	0.3439024
C	4.6989239	-0.8032266	1.1119626
C	6.0302078	-1.2035747	1.0727045
C	6.9315961	-0.5119366	0.2625784
C	6.5153146	0.5725363	-0.5073480
C	5.1789602	0.9631400	-0.4665663
Cl	8.6034723	-1.0106895	0.2142969
N	1.1084336	-3.1261783	-2.5259248
C	1.3279998	-2.3381162	-3.7396558
C	0.5028471	-1.0684485	-3.7528410
C	1.1067951	0.1866997	-3.6310421
C	0.3396651	1.3483638	-3.6233390
C	-1.0546188	1.2796821	-3.7277424
C	-1.6599401	0.0245393	-3.8380645
C	-0.8914832	-1.1388737	-3.8586994
C	-1.8749346	2.5541107	-3.7070882
N	-1.5937350	3.3529701	-2.5167555
C	-0.7338238	4.4174806	-2.5642493

O	-0.3260041	4.8933937	-3.6270010
C	0.2042858	-4.1486445	-2.4916022
O	-0.4408431	-4.4949825	-3.4872161
C	0.0408142	-4.8503389	-1.1736083
C	-0.6000214	-6.0953072	-1.1660131
C	-0.7781903	-6.7823367	0.0337818
C	-0.3305560	-6.2321560	1.2335268
C	0.3066174	-4.9850973	1.2403318
C	0.4816032	-4.3020226	0.0337341
C	0.7336223	-4.4175564	2.5644271
N	1.5937541	-3.3531844	2.5169330
C	1.8752497	-2.5546273	3.7074288
C	1.0552600	-1.2799874	3.7281953
C	-0.3390850	-1.3484402	3.6244390
C	-1.1059981	-0.1866293	3.6321311
C	-0.5017505	1.0684331	3.7532855
C	0.8926389	1.1386263	3.8585501
C	1.6608697	-0.0249326	3.8379041
C	-1.3266973	2.3382350	3.7401698
N	-1.1074666	3.1261941	2.5263008
C	-0.2033434	4.1486454	2.4915627
O	0.4428579	4.4943875	3.4866927
O	0.3263581	-4.8938929	3.6272087
C	-0.3075507	4.9855344	-1.2401435
C	-0.4815179	4.3020633	-0.0336125
C	-0.0411279	4.8508193	1.1736975
C	0.5982033	6.0965490	1.1661711
C	0.7752613	6.7839671	-0.0335735
C	0.3280820	6.2333834	-1.2332844
H	-1.2691225	-7.7514074	0.0335409

H	-0.9485522	-6.5012190	-2.1106365
H	0.9287143	-3.3123485	0.0370265
H	-0.4686439	-6.7446497	2.1804361
H	1.8063846	-2.9206598	1.6229625
H	1.5670210	-2.8047819	-1.6787232
H	2.3939408	-2.0996784	-3.8029628
H	1.0583338	-2.9813035	-4.5813769
H	1.6318984	-3.1891240	4.5644444
H	2.9449868	-2.3217198	3.7459780
H	2.1898448	0.2537655	-3.5472281
H	0.8205891	2.3201985	-3.5432926
H	-2.7430749	-0.0445823	-3.9198900
H	-1.3649520	-2.1118088	-3.9647896
H	-0.8202266	-2.3202080	3.5449067
H	-2.1890982	-0.2535243	3.5488359
H	1.3663364	2.1115019	3.9641440
H	2.7440551	0.0440115	3.9192061
H	-2.9446144	2.3210027	-3.7457199
H	-1.6316117	3.1885028	-4.5642065
H	-1.0566527	2.9814708	4.5817491
H	-2.3926470	2.0999675	3.8038724
H	-1.8076388	2.9213710	-1.6226570
H	-1.5681704	2.8062901	1.6797169
H	-0.9272100	3.3117156	-0.0369072
H	1.2649928	7.7536447	-0.0333046
H	0.9465539	6.5027025	2.1107534
H	0.4653773	6.7461875	-2.1801421
C	-2.6385397	-2.1581030	-0.5917994
H	-0.3233479	-1.3243537	-0.6859120
H	0.3233598	1.3237737	0.6858194

C	2.6384133	2.1577083	0.5909320
H	-4.8468456	-1.7971892	1.0783713
H	-7.2246631	-1.0985314	1.1367966
H	-6.3709713	2.0419849	-1.6699735
H	-3.9938955	1.3302715	-1.7422552
H	4.8470036	1.7964050	-1.0791089
H	7.2248540	1.0978797	-1.1370507
H	6.3709701	-2.0419380	1.6704561
H	3.9938729	-1.3303043	1.7423027
H	-3.5928735	-2.6525581	-0.7670086
H	-2.0112820	-2.3122062	-1.4732463
H	-2.1578444	-2.6022920	0.2836644
H	3.5927399	2.6524269	0.7653911
H	2.1570257	2.6014663	-0.2843623
H	2.0116632	2.3119493	1.4727266

Table 18: Cartesian coordinates and total energy in a.u. of complex **2d@1**

E(RI-TPSS-D3/def2-TZVP)=-3296.040016287

C	-0.1816330	6.1587515	-1.5699167
C	-0.7465092	4.8891567	-1.3975342
C	-0.6202082	4.2493253	-0.1609080
C	0.0518988	4.8635443	0.9003869
C	0.6196810	6.1292162	0.7114824
C	0.4945091	6.7736366	-0.5179702
C	-1.4167443	4.2502290	-2.5838134
O	-1.2507887	4.6924498	-3.7239423

C	0.2094727	4.2176121	2.2474007
O	1.0666282	4.6024459	3.0507365
N	-2.2027449	3.1623230	-2.3228611
C	-2.6894268	2.2995440	-3.3934569
C	-1.8324976	1.0555590	-3.5484888
C	-0.4366822	1.1456250	-3.5114294
C	0.3497885	0.0027684	-3.6273212
C	-0.2355046	-1.2573373	-3.7854171
C	-1.6314663	-1.3472221	-3.8466931
C	-2.4174736	-0.2017701	-3.7256617
C	0.6147568	-2.5087631	-3.8330751
N	0.6572031	-3.2040621	-2.5417393
C	-0.2102052	-4.2175532	-2.2478445
O	-1.0679081	-4.6024203	-3.0505745
C	-0.0511306	-4.8638349	-0.9011939
C	0.6201943	-4.2488830	0.1601714
C	0.7481955	-4.8892196	1.3963655
C	0.1857157	-6.1599318	1.5682960
C	-0.4897009	-6.7754994	0.5162845
C	-0.6165127	-6.1306442	-0.7127722
C	1.4177874	-4.2497013	2.5826680
O	1.2519187	-4.6918150	3.7228496
N	2.2031632	-3.1613833	2.3216021
C	2.6895018	-2.2982551	3.3920410
C	1.8320345	-1.0546292	3.5470867
C	0.4362750	-1.1451155	3.5092217
C	-0.3506293	-0.0025703	3.6253176
C	0.2341698	1.2576395	3.7843647
C	1.6300816	1.3479367	3.8464321
C	2.4165127	0.2027954	3.7252659

C	-0.6165038	2.5087962	3.8320398
N	-0.6584913	3.2044751	2.5408971
O	2.1230523	-1.3453604	-0.2381322
C	1.9075347	-0.1470004	0.0261596
N	2.9182492	0.7642097	0.2217097
C	4.2765836	0.3546776	0.3966247
C	4.8536513	-0.6756419	-0.3589356
C	6.1791497	-1.0364748	-0.1333015
C	6.9739447	-0.3961716	0.8333488
C	6.3794332	0.6441866	1.5656269
C	5.0553265	1.0186587	1.3541311
Si	8.7556072	-0.9036673	1.1369877
C	0.5247602	0.3613761	0.2038643
C	-0.5249362	-0.3609894	-0.2056585
C	-1.9077223	0.1472513	-0.0275629
O	-2.1233841	1.3456800	0.2363580
N	-2.9183753	-0.7642118	-0.2219918
C	-4.2769620	-0.3547895	-0.3954571
C	-5.0563082	-1.0177542	-1.3531303
C	-6.3807392	-0.6434297	-1.5628869
C	-6.9748764	0.3957836	-0.8286711
C	-6.1794200	1.0350614	0.1381160
C	-4.8536067	0.6743266	0.3620121
Si	-8.7568607	0.9031967	-1.1305781
H	-0.9225164	-7.7621524	0.6556229
H	-1.1482620	-6.5872375	-1.5416689
H	1.0083096	-3.2422034	0.0356398
H	0.2825798	-6.6379956	2.5382040
H	2.2468672	-2.7774704	1.3821683
H	1.3023217	-2.8581405	-1.8393758

H	1.6432014	-2.2610589	-4.1132021
H	0.2129025	-3.2216318	-4.5574682
H	2.6704144	-2.8983733	4.3071352
H	3.7265439	-2.0152910	3.1824141
H	1.4341088	0.0892689	-3.5877922
H	0.0347060	2.1168704	-3.3883159
H	-3.5012170	-0.2839729	-3.7627871
H	-2.0918737	-2.3234033	-3.9773309
H	-0.0347442	-2.1164375	3.3853333
H	-1.4349011	-0.0894139	3.5851615
H	2.0901028	2.3241953	3.9778215
H	3.5002090	0.2853186	3.7630244
H	-3.7266161	2.0170469	-3.1839522
H	-2.6699655	2.8997539	-4.3084761
H	-0.2152375	3.2215515	4.5568612
H	-1.6450182	2.2607034	4.1115696
H	-2.2459524	2.7778553	-1.3836246
H	-1.3023994	2.8578833	1.8377533
H	-1.0105151	3.2435747	-0.0358479
H	0.9291452	7.7594389	-0.6576592
H	1.1519038	6.5852940	1.5403570
H	-0.2772770	6.6364747	-2.5401117
C	-2.6223007	-2.2060943	-0.3780558
H	-0.3544573	-1.2967489	-0.7254705
H	0.3540994	1.2971133	0.7236525
C	2.6221512	2.2061241	0.3774618
H	-4.6257103	-1.8154551	-1.9499903
H	-6.9549845	-1.1725904	-2.3196262
H	-6.5994458	1.8347206	0.7449645
H	-4.2703797	1.1826727	1.1166271

H	4.6244350	1.8171810	1.9496648
H	6.9530920	1.1741027	2.3222829
H	6.5994089	-1.8371835	-0.7386038
H	4.2710364	-1.1848638	-1.1134078
H	-9.3511627	-0.0185111	-2.1303574
H	-8.8273313	2.2954864	-1.6436117
H	-9.5375161	0.8390206	0.1310728
H	9.3473140	0.0142872	2.1417351
H	8.8255791	-2.2980427	1.6443782
H	9.5390350	-0.8338958	-0.1226675
H	-1.8273585	-2.4960625	0.3087250
H	-3.5195579	-2.7644035	-0.1099826
H	-2.3329945	-2.4566707	-1.4028242
H	3.5194650	2.7644025	0.1094927
H	1.8273866	2.4959521	-0.3095705
H	2.3325861	2.4568717	1.4021190

Table 19: Cartesian coordinates and total energy in a.u. of complex **2e@1**

E(RI-TPSS-D3/def2-TZVP)=-2714.577052918

C	-5.0358483	-0.7409196	-1.4780077
C	-4.2719947	-0.2314802	-0.4213814
C	-4.8255314	0.7211379	0.4407709
C	-6.1305492	1.1621918	0.2347899
C	-6.8924844	0.6612060	-0.8217871
C	-6.3414273	-0.2949363	-1.6742559
N	-2.9343914	-0.7119867	-0.2336481

C	-1.9007858	0.1735013	-0.0518945
C	-0.5360816	-0.3999187	-0.0315739
C	0.5343775	0.4020126	0.0296840
C	1.8989704	-0.1717273	0.0503384
O	2.0894784	-1.3988519	-0.0683044
O	-2.0916263	1.4006314	0.0662945
N	2.9327622	0.7133630	0.2331358
C	4.2700649	0.2322850	0.4214724
C	4.8236509	-0.7204115	-0.4405817
C	6.1283371	-1.1621368	-0.2339719
C	6.8899384	-0.6617482	0.8231288
C	6.3388849	0.2945256	1.6754463
C	5.0336311	0.7411878	1.4785684
N	0.8739287	-3.1782297	-2.4477683
C	0.9865875	-2.4554715	-3.7164805
C	0.1456771	-1.1970293	-3.7375172
C	0.7375814	0.0675040	-3.6694785
C	-0.0428428	1.2204098	-3.6473875
C	-1.4393429	1.1339264	-3.6862092
C	-2.0311502	-0.1300780	-3.7575972
C	-1.2506573	-1.2849319	-3.7891767
C	-2.2831442	2.3939116	-3.6356713
N	-1.8935693	3.2511109	-2.5194285
C	-1.0327692	4.2993928	-2.6919933
O	-0.7165604	4.7239277	-3.8073255
C	-0.0006566	-4.2158390	-2.2933794
O	-0.7109899	-4.6243559	-3.2191448
C	-0.0403038	-4.8618235	-0.9366885
C	-0.6982660	-6.0916612	-0.8129222
C	-0.7592760	-6.7303928	0.4249160

C	-0.1785547	-6.1466200	1.5494565
C	0.4788823	-4.9150089	1.4408031
C	0.5391791	-4.2830454	0.1960095
C	1.0415369	-4.2985812	2.6921582
N	1.8989780	-3.2479120	2.5173165
C	2.2880212	-2.3889228	3.6322982
C	1.4406535	-1.1313226	3.6836640
C	0.0444512	-1.2213130	3.6427534
C	-0.7390162	-0.0704659	3.6655978
C	-0.1505128	1.1954850	3.7364526
C	1.2455344	1.2868897	3.7904859
C	2.0290311	0.1341116	3.7581750
C	-0.9945356	2.4518609	3.7152086
N	-0.8800174	3.1765575	2.4477442
C	-0.0054553	4.2146696	2.2966411
O	0.7012267	4.6234758	3.2250729
O	0.7293876	-4.7239457	3.8083128
C	-0.4718117	4.9143778	-1.4391713
C	-0.5383670	4.2832959	-0.1942573
C	0.0396017	4.8604718	0.9400195
C	0.7020025	6.0880912	0.8178138
C	0.7692486	6.7259962	-0.4201284
C	0.1902904	6.1436221	-1.5463096
H	7.9060907	-1.0115458	0.9786254
H	-7.9089005	1.0104621	-0.9767795
H	-1.2648494	-7.6878605	0.5135211
H	-1.1518609	-6.5248079	-1.6989734
H	1.0137262	-3.3107235	0.1157712
H	-0.2281790	-6.6193542	2.5254556
H	2.0484400	-2.8617996	1.5899669

H	1.3584163	-2.7828639	-1.6468959
H	2.0415485	-2.2093558	-3.8755555
H	0.6652007	-3.1491402	-4.4975537
H	2.1592218	-2.9830325	4.5418242
H	3.3464751	-2.1284828	3.5302458
H	1.8224084	0.1506465	-3.6348335
H	0.4311136	2.1977279	-3.6050985
H	-3.1154685	-0.2085274	-3.7907966
H	-1.7151065	-2.2659532	-3.8547479
H	-0.4270154	-2.1997255	3.5980819
H	-1.8235709	-0.1563817	3.6290934
H	1.7073737	2.2689872	3.8582055
H	3.1130900	0.2153242	3.7931649
H	-3.3425326	2.1365681	-3.5356086
H	-2.1509448	2.9882050	-4.5445722
H	-0.6770253	3.1451531	4.4981715
H	-2.0493238	2.2028993	3.8709237
H	-2.0458775	2.8649045	-1.5925962
H	-1.3607953	2.7804094	1.6449680
H	-1.0168132	3.3128243	-0.1150885
H	1.2783771	7.6816879	-0.5075433
H	1.1541858	6.5201058	1.7051394
H	0.2447626	6.6156648	-2.5223813
C	-2.7031588	-2.1656523	-0.3926642
H	-0.3838947	-1.4701389	-0.0890880
H	0.3823085	1.4722601	0.0871796
C	2.7018263	2.1669279	0.3933817
H	-4.6081032	-1.4769513	-2.1517968
H	-6.9234110	-0.6939236	-2.5000089
H	-6.5548937	1.8996847	0.9099046

H	-4.2368225	1.1103552	1.2618092
H	4.6059238	1.4773284	2.1522493
H	6.9206143	0.6930949	2.5015806
H	6.5526943	-1.8996911	-0.9090120
H	4.2352284	-1.1091562	-1.2620440
H	-3.6712368	-2.6629613	-0.3586483
H	-2.2125564	-2.3949269	-1.3428825
H	-2.0990498	-2.5459752	0.4332188
H	3.6699626	2.6641269	0.3592419
H	2.0973668	2.5480026	-0.4318801
H	2.2116788	2.3955076	1.3440173

Table 20: Cartesian coordinates and total energy in a.u. of complex **2f@1**

E(RI-TPSS-D3/def2-TZVP)=-3029.268726161

C	0.3676077	6.1989916	-1.2122111
C	-0.2985275	4.9671156	-1.2130586
C	-0.4863254	4.2926332	-0.0036956
C	-0.0302989	4.8345974	1.2007901
C	0.6389274	6.0644808	1.1871982
C	0.8310564	6.7425772	-0.0155166
C	-0.7418937	4.4056840	-2.5345836
O	-0.3437667	4.8856374	-3.5997769
C	-0.2051388	4.1413793	2.5221489
O	0.4279220	4.4993361	3.5217896
N	-1.6037576	3.3436294	-2.4832389
C	-1.8987346	2.5520548	-3.6753019

C	-1.0753905	1.2800450	-3.7137097
C	0.3198999	1.3519800	-3.6270468
C	1.0897075	0.1922393	-3.6459822
C	0.4870718	-1.0639325	-3.7624431
C	-0.9082796	-1.1374749	-3.8513470
C	-1.6792902	0.0238207	-3.8187451
C	1.3149360	-2.3318851	-3.7561523
N	1.1014825	-3.1208617	-2.5420566
C	0.1989070	-4.1443982	-2.5064714
C	0.0259192	-4.8372729	-1.1847298
C	-0.6446041	-6.0664219	-1.1693531
C	-0.8341657	-6.7442232	0.0339390
C	-0.3667468	-6.2011573	1.2293584
C	0.3006013	-4.9699461	1.2284118
C	0.4855209	-4.2956640	0.0184988
C	0.7487877	-4.4091550	2.5486327
O	0.3559281	-4.8908450	3.6150056
O	-0.4384408	-4.4998926	-3.5042652
N	1.6091501	-3.3459118	2.4946386
C	1.9060947	-2.5536689	3.6858672
C	1.0804525	-1.2832155	3.7257788
C	-0.3149654	-1.3580495	3.6438987
C	-1.0869982	-0.1997839	3.6628869
C	-0.4864937	1.0577982	3.7747605
C	0.9089804	1.1342488	3.8590854
C	1.6821907	-0.0255788	3.8264068
C	-1.3169769	2.3240394	3.7683775
H	-1.3496478	-7.7005386	0.0398595
H	-1.0071730	-6.4669561	-2.1110010
H	0.9569452	-3.3175948	0.0162358

H	-0.5120344	-6.7066718	2.1789527
H	1.8077061	-2.9045567	1.6012234
H	1.5451996	-2.7832162	-1.6925927
H	2.3800909	-2.0907467	-3.8236004
H	1.0427717	-2.9750054	-4.5970728
H	1.6792848	-3.1940805	4.5429132
H	2.9750132	-2.3154453	3.7070026
H	2.1733356	0.2615820	-3.5723519
H	0.7994899	2.3246333	-3.5494533
H	-2.7633699	-0.0477554	-3.8832646
H	-1.3811969	-2.1113412	-3.9510598
H	-0.7928851	-2.3317923	3.5696343
H	-2.1706953	-0.2714083	3.5927671
H	1.3803888	2.1092007	3.9554446
H	2.7663339	0.0482681	3.8872642
H	-2.9680334	2.3158949	-3.6996916
H	-1.6682900	3.1922461	-4.5315671
H	-1.0471446	2.9672012	4.6100257
H	-2.3816992	2.0805825	3.8345272
H	-1.8090775	2.9048868	-1.5901311
H	-1.5443913	2.7751779	1.7045926
H	-0.9567371	3.3140308	-0.0028843
H	1.3455249	7.6994457	-0.0199776
H	0.9986322	6.4652438	2.1298409
H	0.5152384	6.7044952	-2.1614485
N	-1.1035657	3.1141487	2.5550956
O	2.1132388	-1.3983976	-0.0629215
C	1.8930066	-0.1925175	0.1699993
N	2.9053858	0.7113497	0.3580937
C	2.6403700	2.1538355	0.5551254

C	0.5168160	0.3504753	0.2407376
C	-0.5187141	-0.3552996	-0.2275646
C	-1.8945633	0.1889446	-0.1590069
O	-2.1139634	1.3945933	0.0760077
N	-2.9074761	-0.7131045	-0.3527564
C	-2.6437798	-2.1555753	-0.5517311
C	-4.2747045	-0.2848160	-0.3007597
C	-5.1837755	-0.9594284	0.5134475
C	-6.5259879	-0.5789717	0.5354487
C	-6.9978240	0.4834535	-0.2430438
C	-6.0607031	1.1540025	-1.0476475
C	-4.7223707	0.7837585	-1.0825956
C	-8.4667149	0.9235748	-0.2504078
C	4.2730447	0.2855193	0.2982649
C	4.7275260	-0.7818559	1.0778317
C	6.0663363	-1.1496099	1.0345112
C	6.9971281	-0.4777839	0.2236399
C	6.5184715	0.5834405	-0.5523274
C	5.1757425	0.9614226	-0.5219726
C	8.4668028	-0.9154041	0.2216607
C	-9.3361453	0.0733731	0.6924652
C	-8.5610799	2.4001320	0.1996136
C	-9.0309880	0.7892166	-1.6843611
C	9.3289080	-0.0633298	-0.7262632
C	9.0398988	-0.7807447	1.6520789
C	8.5607431	-2.3916022	-0.2296530
H	-0.3368886	-1.3277444	-0.6692303
H	0.3349572	1.3230906	0.6819764
H	-4.8462905	-1.7805680	1.1401862
H	-7.2028623	-1.1276612	1.1813702

H	-6.3819065	1.9832499	-1.6719954
H	-4.0226993	1.3146087	-1.7166639
H	4.8327910	1.7816211	-1.1469875
H	7.1901979	1.1330359	-1.2028202
H	6.3930292	-1.9780106	1.6571380
H	4.0328731	-1.3138871	1.7164484
H	-3.6015372	-2.6522624	-0.6984096
H	-2.0392001	-2.3112198	-1.4490537
H	-2.1390888	-2.5963352	0.3118168
H	3.5977545	2.6516533	0.7004401
H	2.1344879	2.5930042	-0.3085673
H	2.0363034	2.3100056	1.4526671
H	-10.3718181	0.4263376	0.6495751
H	-9.3324163	-0.9834781	0.4031823
H	-9.0005638	0.1509157	1.7325111
H	-10.0806667	1.1037810	-1.7062055
H	-8.4775906	1.4116654	-2.3946070
H	-8.9748450	-0.2493810	-2.0277520
H	-9.6069046	2.7281992	0.1944679
H	-8.1658789	2.5226140	1.2136869
H	-7.9968141	3.0609316	-0.4658463
H	10.3654826	-0.4143390	-0.6896932
H	8.9872346	-0.1412918	-1.7642970
H	9.3249027	0.9934499	-0.4367301
H	10.0903921	-1.0929184	1.6669435
H	8.9835532	0.2574742	1.9965851
H	8.4925019	-1.4049548	2.3654243
H	9.6070157	-2.7182577	-0.2304425
H	8.0009997	-3.0534929	0.4385367
H	8.1601223	-2.5140395	-1.2416029

Table 21: Cartesian coordinates and total energy in a.u. of complex **2g@1**

E(RI-TPSS-D3/def2-TZVP)=-2865.106176949

C	-0.4728078	-5.6877397	-1.5919420
C	0.5354128	-4.7379591	-1.3845441
C	0.7841340	-4.2825428	-0.0865723
C	0.0082608	-4.7193109	0.9907370
C	-0.9986172	-5.6673276	0.7682985
C	-1.2277420	-6.1551852	-0.5168642
C	1.2405569	-4.1910503	-2.5965636
O	1.0545733	-4.6755700	-3.7162698
C	0.1627464	-4.1737407	2.3824267
O	-0.4510797	-4.6654983	3.3352546
N	2.0418243	-3.1018636	-2.3858013
C	2.4675505	-2.2577160	-3.5008212
C	1.5499656	-1.0565141	-3.6440487
C	2.0617243	0.2413450	-3.7312999
C	1.2103548	1.3423940	-3.8129798
C	-0.1791937	1.1676305	-3.7976297
C	-0.6906153	-0.1311027	-3.7270781
C	0.1610837	-1.2304099	-3.6554482
C	-1.0979767	2.3709926	-3.7850213
N	-1.0034703	3.0965239	-2.5154769
C	-0.1715051	4.1742241	-2.3831962
C	-0.0085584	4.7180236	-0.9918935
C	-0.7844861	4.2862653	0.0873695

C	-0.5271433	4.7373211	1.3851566
C	0.4896220	5.6783923	1.5904569
C	1.2444072	6.1415025	0.5133801
C	1.0066755	5.6575912	-0.7717133
C	-1.2328670	4.1944238	2.5985672
N	-2.0376590	3.1075280	2.3893530
C	-2.4671039	2.2670255	3.5059408
C	-1.5542241	1.0625313	3.6503679
C	-2.0698789	-0.2344470	3.7265879
C	-1.2217775	-1.3380938	3.8074842
C	0.1682307	-1.1667769	3.8022861
C	0.6834284	0.1310193	3.7426500
C	-0.1649911	1.2329131	3.6720944
C	1.0840974	-2.3723668	3.7902678
N	0.9930457	-3.0954607	2.5191754
O	0.4376626	4.6657308	-3.3390850
O	-1.0446631	4.6799705	3.7174702
O	-2.1290815	1.3185143	-0.1190735
C	-1.8983341	0.0949125	-0.0129741
C	-0.5149958	-0.4240578	-0.0337233
C	0.5183041	0.4267902	0.0406010
C	1.9014650	-0.0926999	0.0181747
N	2.9038060	0.8258362	-0.1657851
C	4.2477962	0.3942067	-0.4109786
C	4.8559582	-0.5937884	0.3705081
C	6.1614386	-0.9927781	0.1008993
C	6.8769846	-0.4088489	-0.9497343
C	6.2783548	0.5857317	-1.7276517
C	4.9736433	0.9791895	-1.4574157
O	8.1635078	-0.7624081	-1.2644207

N	-2.9009821	-0.8245678	0.1644832
C	-4.2457830	-0.3943111	0.4075568
C	-4.8520309	0.5968099	-0.3714494
C	-6.1584893	0.9940670	-0.1039896
C	-6.8768845	0.4052665	0.9419686
C	-6.2801465	-0.5924432	1.7173315
C	-4.9744762	-0.9841794	1.4492566
O	-8.1644779	0.7569295	1.2543914
O	2.1321411	-1.3160155	0.1279427
H	-2.0065595	-6.8939594	-0.6843146
H	-0.6489764	-6.0345855	-2.6054124
H	1.5946395	-3.5859398	0.0897788
H	-1.5869911	-6.0003074	1.6177052
H	1.3132075	-2.5943266	1.6922697
H	2.0283313	-2.6379318	-1.4792442
H	3.4981911	-1.9296488	-3.3310072
H	2.4405043	-2.8839347	-4.3972523
H	0.8104808	-3.0838870	4.5727004
H	2.1237818	-2.0630447	3.9406417
H	3.1391524	0.3890045	-3.7298911
H	1.6153847	2.3492386	-3.8811466
H	-1.7684260	-0.2833346	-3.7211998
H	-0.2534379	-2.2337817	-3.6045385
H	-1.6297394	-2.3443001	3.8676492
H	-3.1476400	-0.3793305	3.7169468
H	0.2521008	2.2356510	3.6297686
H	1.7616372	0.2804712	3.7448199
H	-2.1375139	2.0589585	-3.9306600
H	-0.8290143	3.0815706	-4.5698705
H	-2.4373769	2.8946437	4.4012587

H	-3.4990726	1.9429919	3.3364821
H	-1.3174529	2.5950312	-1.6863325
H	-2.0231570	2.6408961	1.4840924
H	-1.6023793	3.5978946	-0.0874033
H	2.0298205	6.8736405	0.6791923
H	0.6721524	6.0222954	2.6038119
H	1.5947294	5.9870680	-1.6227163
C	-2.6147928	-2.2692127	0.3089037
H	-0.3137031	-1.4875449	-0.0817243
H	0.3172072	1.4903445	0.0883961
C	2.6170430	2.2697743	-0.3161332
H	-4.5164116	-1.7492601	2.0679503
H	-6.8431187	-1.0452992	2.5267815
H	-6.6216332	1.7643009	-0.7172155
H	-4.3038819	1.0595821	-1.1814805
H	4.5140776	1.7417568	-2.0780527
H	6.8391148	1.0348335	-2.5407244
H	6.6260795	-1.7605301	0.7160975
H	4.3099966	-1.0528152	1.1841421
H	8.4612663	-1.4605411	-0.6587336
H	-8.4607876	1.4574662	0.6507711
H	-1.9665749	-2.6119700	-0.4989757
H	-2.1468854	-2.4927870	1.2718756
H	-3.5588840	-2.8064380	0.2318455
H	3.5615628	2.8072774	-0.2469945
H	2.1439674	2.4882929	-1.2777339
H	1.9733189	2.6169145	0.4935097

Table 22: Cartesian coordinates and total energy in a.u. of complex **2h@1**

E(RI-TPSS-D3/def2-TZVP)=-2825.356495064

C	0.5965606	6.0869796	-0.7675173
C	-0.0267454	4.8436479	-0.9295037
C	-0.6704579	4.2570732	0.1637530
C	-0.7105496	4.8956663	1.4059873
C	-0.0889982	6.1420157	1.5521172
C	0.5578726	6.7321891	0.4677586
C	0.0337416	4.1959666	-2.2849791
O	0.7871275	4.6249381	-3.1668775
C	-1.3460038	4.2747520	2.6204603
O	-1.1462069	4.7351298	3.7487326
N	-0.8034566	3.1372860	-2.4925377
C	-0.8336626	2.4262810	-3.7732807
C	0.0389145	1.1897727	-3.7654429
C	1.4332482	1.3112125	-3.7975271
C	2.2410665	0.1773453	-3.7234147
C	1.6795051	-1.0986481	-3.6257656
C	0.2853373	-1.2191312	-3.6144265
C	-0.5227635	-0.0874983	-3.6808634
C	2.5547845	-2.3331499	-3.5126437
N	2.1248490	-3.1895748	-2.4111560
C	1.3267835	-4.2787293	-2.6268605
O	1.1191746	-4.7381686	-3.7541468
C	0.6931286	-4.8957225	-1.4093827
C	0.6667957	-4.2589890	-0.1658229
C	0.0246892	-4.8413489	0.9306369
C	-0.6101657	-6.0791336	0.7707876

C	-0.5851677	-6.7226261	-0.4657497
C	0.0596259	-6.1362969	-1.5534613
C	-0.0234726	-4.1941244	2.2868811
O	-0.7700775	-4.6225750	3.1748001
N	0.8159125	-3.1358537	2.4878052
C	0.8510156	-2.4206608	3.7661709
C	-0.0275013	-1.1883297	3.7586382
C	-1.4210385	-1.3163826	3.8010641
C	-2.2348497	-0.1870004	3.7250557
C	-1.6804057	1.0911201	3.6148239
C	-0.2870042	1.2183099	3.5936238
C	0.5271915	0.0911315	3.6623212
C	-2.5634254	2.3201007	3.5001108
N	-2.1363290	3.1806750	2.4009881
O	2.1126981	-1.3647400	0.1408180
C	1.9031454	-0.1405262	-0.0095927
N	2.9271034	0.7545883	-0.1884152
C	4.2740823	0.2996543	-0.3630472
C	4.8377989	-0.6809018	0.4624968
C	6.1478101	-1.0962097	0.2666869
C	6.9399813	-0.5478597	-0.7572467
C	6.3714888	0.4425645	-1.5755550
C	5.0601826	0.8565526	-1.3793082
N	8.2731600	-0.9223471	-0.9083076
C	0.5313420	0.4061127	-0.0367450
C	-0.5289687	-0.4123388	0.0250288
C	-1.9003745	0.1356373	0.0005279
O	-2.1086821	1.3597244	-0.1524613
N	-2.9249271	-0.7574926	0.1864327
C	-4.2701305	-0.3001156	0.3679856

C	-5.0520601	-0.8559950	1.3881028
C	-6.3611523	-0.4388439	1.5922256
C	-6.9318068	0.5537956	0.7781198
C	-6.1441761	1.1007027	-0.2500733
C	-4.8363307	0.6823199	-0.4537523
N	-8.2630242	0.9317862	0.9373539
H	-1.0735652	-7.6860741	-0.5824511
H	0.0788426	-6.6136854	-2.5283123
H	1.1243221	-3.2814125	-0.0592775
H	-1.1133037	-6.5145877	1.6284754
H	1.3261632	-2.7213189	1.7123348
H	2.1882881	-2.7766030	-1.4841947
H	3.5983054	-2.0414870	-3.3539683
H	2.4941732	-2.9405322	-4.4206870
H	0.5179493	-3.1301908	4.5276654
H	1.8905709	-2.1443465	3.9708910
H	3.3234255	0.2819845	-3.7360479
H	1.8745136	2.3021099	-3.8737363
H	-1.6056405	-0.1968041	-3.6615105
H	-0.1660775	-2.2059509	-3.5515220
H	-1.8569229	-2.3089146	3.8859880
H	-3.3165505	-0.2968520	3.7459434
H	0.1593710	2.2067128	3.5209335
H	1.6093601	0.2057082	3.6348727
H	-1.8731612	2.1555365	-3.9856299
H	-0.4925511	3.1369874	-4.5301822
H	-2.5095769	2.9267934	4.4091020
H	-3.6045053	2.0214336	3.3383178
H	-1.3214749	2.7239793	-1.7216635
H	-2.1951339	2.7688810	1.4731672

H	-1.1179825	3.2749426	0.0559512
H	1.0371337	7.7000210	0.5860923
H	-0.1187289	6.6207669	2.5260440
H	1.1015316	6.5254208	-1.6225992
C	-2.6769136	-2.2070111	0.3529708
H	-0.3589498	-1.4763210	0.1280082
H	0.3610317	1.4700285	-0.1396318
C	2.6781519	2.2043949	-0.3512011
H	-4.6337030	-1.6152300	2.0416936
H	-6.9465208	-0.8855179	2.3921792
H	-6.5671085	1.8603458	-0.9033654
H	-4.2509019	1.1214572	-1.2508182
H	4.6435038	1.6142703	-2.0358029
H	6.9603259	0.8900053	-2.3725348
H	6.5688705	-1.8544046	0.9228680
H	4.2486897	-1.1209334	1.2564208
H	8.6775761	-0.7582861	-1.8220371
H	8.5223003	-1.8294806	-0.5339048
H	-8.5119510	1.8397731	0.5648780
H	-8.6624128	0.7683499	1.8533971
H	-2.0067353	-2.5700744	-0.4275648
H	-2.2525757	-2.4345018	1.3352035
H	-3.6302550	-2.7222793	0.2438628
H	3.6309494	2.7200266	-0.2392710
H	2.2551877	2.4344148	-1.3333935
H	2.0064438	2.5647618	0.4293329

Table 23: Cartesian coordinates and total energy in a.u. of complex **2a′@1**

E(RI-TPSS-D3/def2-TZVP)=-3123.835720823

C	-5.1931071	-1.0085134	0.6139446
C	-4.2668425	-0.3523588	-0.2042149
C	-4.6800679	0.7108343	-1.0071694
C	-6.0136998	1.0907679	-0.9689608
C	-6.9573031	0.4553850	-0.1653808
C	-6.5296642	-0.6068446	0.6261802
N	-2.9057495	-0.7811166	-0.2424796
C	-1.9002370	0.1449666	-0.0846171
C	-0.5189005	-0.3762623	-0.1895966
C	0.5194468	0.3772818	0.1884095
C	1.9007975	-0.1440729	0.0838609
O	2.1397201	-1.3429783	-0.1463598
N	-6.4452405	2.2130910	-1.8409185
O	-5.5911518	2.7355384	-2.5643047
O	-2.1391576	1.3440154	0.1450533
N	2.9063711	0.7818151	0.2429624
C	4.2673963	0.3529793	0.2050715
C	4.6798129	-0.7118292	1.0063224
C	6.0134266	-1.0918386	0.9686131
C	6.9578782	-0.4549823	0.1671919
C	6.5310666	0.6088311	-0.6226869
C	5.1945312	1.0105850	-0.6109651
N	6.4439333	-2.2160198	1.8386795
O	5.5886737	-2.7409490	2.5588911
O	-7.6291172	2.5504869	-1.7904247
O	7.6281601	-2.5524336	1.7898619

N	0.9489269	-3.0326059	-2.5740203
C	1.1113174	-2.2245960	-3.7834175
C	0.2860830	-0.9551892	-3.7497990
C	0.8989796	0.2990573	-3.6751268
C	0.1380540	1.4641654	-3.6425578
C	-1.2607023	1.4021519	-3.6760705
C	-1.8744972	0.1476770	-3.7398793
C	-1.1121599	-1.0201115	-3.7820599
C	-2.0719027	2.6832140	-3.6283222
N	-1.7371080	3.4766663	-2.4464483
C	-0.8096686	4.4787642	-2.4976704
O	-0.3635685	4.9200948	-3.5604203
C	0.0629321	-4.0698059	-2.5231614
O	-0.6271664	-4.3957606	-3.4953435
C	-0.0305058	-4.8097826	-1.2193161
C	-0.6826818	-6.0488649	-1.2174745
C	-0.8137691	-6.7693709	-0.0313851
C	-0.3115062	-6.2557775	1.1626017
C	0.3415692	-5.0171560	1.1752156
C	0.4747857	-4.3023090	-0.0184983
C	0.8091867	-4.4785658	2.4976950
N	1.7370498	-3.4768863	2.4467201
C	2.0717369	-2.6831403	3.6283835
C	1.2604078	-1.4021396	3.6755965
C	-0.1383484	-1.4643793	3.6422115
C	-0.8994544	-0.2993754	3.6745295
C	-0.2867527	0.9549706	3.7491506
C	1.1114801	1.0200985	3.7814552
C	1.8740045	-0.1475746	3.7394369
C	-1.1121980	2.2242692	3.7825941

N	-0.9496821	3.0324680	2.5733362
C	-0.0638093	4.0698404	2.5230090
O	0.6255425	4.3960300	3.4956519
O	0.3621482	-4.9192356	3.5603244
C	-0.3414707	5.0172945	-1.1753564
C	-0.4750125	4.3026735	0.0184055
C	0.0303947	4.8098586	1.2192546
C	0.6831764	6.0486170	1.2174040
C	0.8147097	6.7689355	0.0312381
C	0.3122191	6.2555891	-1.1627718
H	7.9871199	-0.7922298	0.1749054
H	-7.9865968	0.7924847	-0.1728180
H	-1.3137954	-7.7337594	-0.0375637
H	-1.0798968	-6.4224281	-2.1561616
H	0.9356901	-3.3183761	-0.0027820
H	-0.4201930	-6.7897938	2.1014286
H	2.0048187	-3.0910954	1.5483353
H	1.4557133	-2.7339731	-1.7464472
H	2.1730579	-1.9824286	-3.8908056
H	0.8073424	-2.8570954	-4.6218050
H	1.8491627	-3.3132497	4.4939818
H	3.1446169	-2.4703423	3.6219068
H	1.9854026	0.3629273	-3.6526336
H	0.6263019	2.4345540	-3.6035897
H	-2.9603188	0.0826338	-3.7763549
H	-1.5946886	-1.9916851	-3.8569901
H	-0.6264460	-2.4348724	3.6039296
H	-1.9858632	-0.3633997	3.6517582
H	1.5938820	1.9917551	3.8561051
H	2.9598014	-0.0823752	3.7763941

H	-3.1447852	2.4704360	-3.6218517
H	-1.8494284	3.3135871	-4.4937553
H	-0.8085734	2.8567550	4.6211138
H	-2.1739163	1.9818881	3.8897270
H	-2.0033865	3.0896035	-1.5480925
H	-1.4552498	2.7330469	1.7452364
H	-0.9363583	3.3190120	0.0027057
H	1.3152392	7.7330688	0.0373836
H	1.0804436	6.4220811	2.1561104
H	0.4211808	6.7895331	-2.1016099
C	-2.6317713	-2.2234559	-0.4323433
H	-0.3368497	-1.3662628	-0.5895673
H	0.3373918	1.3674635	0.5879146
C	2.6323367	2.2240173	0.4337849
H	-4.8665921	-1.8266429	1.2492128
H	-7.2387500	-1.1218791	1.2664356
H	-3.9873249	1.2263360	-1.6555424
H	4.8687239	1.8298894	-1.2450746
H	7.2408048	1.1250746	-1.2612425
H	3.9864514	-1.2285910	1.6530164
H	-3.5860070	-2.7363029	-0.5433974
H	-2.0556367	-2.3812237	-1.3477611
H	-2.0947100	-2.6452606	0.4204523
H	3.5864679	2.7366170	0.5468998
H	2.0966478	2.6467792	-0.4194034
H	2.0548250	2.3809713	1.3484566

Table 24: Cartesian coordinates and total energy in a.u. of complex **2g′@1**

E(RI-TPSS-D3/def2-TZVP)=-2865.105273388

C	-5.1630982	-0.9412418	-0.6510490
C	-4.2874462	-0.2880863	0.2240436
C	-4.7399925	0.7442674	1.0424054
C	-6.0787646	1.1298396	0.9849899
C	-6.9667663	0.4841370	0.1178525
C	-6.4989850	-0.5484190	-0.6925959
N	-2.9180814	-0.7049279	0.2994552
C	-1.9105102	0.2185926	0.1832311
C	-0.5316004	-0.3103112	0.2867902
C	0.5146930	0.4317799	-0.0936496
C	1.8830081	-0.1374350	-0.0598735
O	2.0736630	-1.3475329	0.1712117
O	-6.4593703	2.1490989	1.8212421
O	-2.1371914	1.4275765	-0.0188316
N	2.9100576	0.7296183	-0.3406039
C	4.2430400	0.2453139	-0.5414230
C	4.8107725	-0.6667948	0.3475358
C	6.1109268	-1.1221203	0.1236768
C	6.8476597	-0.6691330	-0.9751389
C	6.2710317	0.2533693	-1.8459838
C	4.9742783	0.7161525	-1.6410792
O	6.6104149	-2.0175362	1.0358537
N	1.8290988	-3.2279445	-2.4107585
C	2.1756851	-2.3771181	-3.5439586
C	1.2939638	-1.1435000	-3.6121915
C	1.8470277	0.1239025	-3.8161494

C	1.0382117	1.2578874	-3.8714575
C	-0.3484919	1.1467560	-3.7085957
C	-0.9023681	-0.1215141	-3.5106530
C	-0.0933993	-1.2542423	-3.4668042
C	-1.2145631	2.3889441	-3.6958529
N	-1.0443669	3.1622657	-2.4626685
C	-0.1308630	4.1726593	-2.3778620
O	0.5443077	4.5356019	-3.3483433
C	1.0097520	-4.3123619	-2.5570888
O	0.6932142	-4.7631538	-3.6620439
C	0.4987859	-4.9352289	-1.2875143
C	-0.0788871	-6.2085768	-1.3638794
C	-0.6078811	-6.8062130	-0.2213896
C	-0.5710582	-6.1409881	1.0031858
C	0.0093012	-4.8701046	1.0944256
C	0.5318403	-4.2737916	-0.0568450
C	0.0321230	-4.2005133	2.4392069
N	0.8957002	-3.1510739	2.5768656
C	1.0162577	-2.4156164	3.8365114
C	0.2117525	-1.1331475	3.8352270
C	-1.1871930	-1.1800855	3.8270418
C	-1.9328506	-0.0016675	3.7952999
C	-1.2998835	1.2447403	3.7847126
C	0.0993839	1.2897239	3.7965780
C	0.8438645	0.1138024	3.8174108
C	-2.0911006	2.5371923	3.7359687
N	-1.7262033	3.3380966	2.5687438
C	-0.8328984	4.3693368	2.6650572
O	-0.4488325	4.8163937	3.7496440
O	-0.6796653	-4.5974963	3.3681922

C	0.0103768	4.8417895	-1.0397026
C	-0.5040085	4.2955013	0.1394512
C	-0.3336438	4.9413160	1.3671472
C	0.3696055	6.1514691	1.4101124
C	0.8861239	6.7021544	0.2386400
C	0.7145258	6.0507492	-0.9817544
H	7.8594712	-1.0306793	-1.1446619
H	-8.0107291	0.7868138	0.0775135
H	-1.0525844	-7.7953663	-0.2851435
H	-0.1089210	-6.7029536	-2.3299354
H	0.9323233	-3.2658705	-0.0028659
H	-0.9846804	-6.5845538	1.9035826
H	1.4132454	-2.7951414	1.7790255
H	1.9946072	-2.8348811	-1.4889723
H	3.2274197	-2.0836232	-3.4623959
H	2.0526506	-2.9911582	-4.4413514
H	0.6649872	-3.0906096	4.6216350
H	2.0756814	-2.1984222	4.0065098
H	2.9241449	0.2217018	-3.9326029
H	1.4724888	2.2411117	-4.0337055
H	-1.9790496	-0.2219354	-3.3854552
H	-0.5394105	-2.2342714	-3.3197351
H	-1.6842490	-2.1468891	3.8544625
H	-3.0197677	-0.0529701	3.7874468
H	0.6009507	2.2541696	3.7947476
H	1.9309915	0.1638216	3.8257360
H	-2.2712858	2.1183803	-3.7804408
H	-0.9504958	3.0539764	-4.5216666
H	-1.8785568	3.1574398	4.6111897
H	-3.1663902	2.3318496	3.7114688

H	-1.5149305	2.8122749	-1.6329603
H	-1.9210385	2.9251836	1.6615757
H	-1.0084517	3.3348598	0.1058607
H	1.4281061	7.6429329	0.2771022
H	0.5036040	6.6344857	2.3729778
H	1.1180182	6.4563191	-1.9043852
C	-2.6451224	-2.1520412	0.4462361
H	-0.3566436	-1.3088992	0.6681004
H	0.3415580	1.4270711	-0.4845538
C	2.6736944	2.1743168	-0.5600645
H	-4.8017244	-1.7340592	-1.2981642
H	-7.1843629	-1.0476135	-1.3710673
H	-4.0672575	1.2509734	1.7208171
H	4.5308771	1.4241303	-2.3325758
H	6.8383379	0.6098788	-2.7006672
H	4.2608744	-1.0308741	1.2046253
H	-7.4074644	2.3215427	1.7037747
H	7.5183053	-2.2527100	0.7853639
H	-3.6014671	-2.6647219	0.5340848
H	-2.1027531	-2.5487967	-0.4158828
H	-2.0737473	-2.3398753	1.3589562
H	3.6339824	2.6834195	-0.4885580
H	2.2338789	2.3691559	-1.5430081
H	2.0203459	2.5709449	0.2182114

0.4 COSMO results on selected structures

Table 25: Cartesian coordinates and total energy in a.u. of complex **3a@1**

E(RI-TPSS-D3/def2-TZVP)=

C	-4.7555616	1.1516193	0.3380627
C	-4.0239531	0.0462900	0.7853146
C	-4.6951958	-1.1503891	1.0683015
C	-6.0768308	-1.2345867	0.9090590
C	-6.8004514	-0.1296909	0.4636158
C	-6.1441132	1.0758661	0.1849405
C	-2.5279135	0.0650273	0.9276062
O	-1.8779558	-0.9885970	0.9728751
C	-6.9800338	2.2401039	-0.2647992
O	-8.1206706	2.0760640	-0.7206540
N	-1.9371912	1.2903119	0.9989668
C	-0.4948856	1.4577542	0.8788278
C	-0.0679513	1.8205966	-0.5347012
C	-0.7885785	1.3761467	-1.6477682
C	-0.3865553	1.7175449	-2.9379685
C	0.7459713	2.5109102	-3.1477867
C	1.4827764	2.9366747	-2.0348281
C	1.0780273	2.5950904	-0.7456354
C	1.1289286	2.9585895	-4.5427409
N	0.5420160	4.2659530	-4.8663889
C	1.1725355	5.4326156	-4.5652132
O	2.3084608	5.4596230	-4.0684148
C	0.4275133	6.6995120	-4.8735351
C	-0.9608336	6.7332477	-5.0357609
C	-1.6249040	7.9369654	-5.2956238
C	-0.8800942	9.1197939	-5.3911564

C	0.5044406	9.0930111	-5.2302152
C	1.1581349	7.8910087	-4.9643028
C	-3.1183932	8.0302458	-5.4438861
O	-3.6960205	9.1264612	-5.4291867
N	-3.7876545	6.8538983	-5.5960505
C	-5.2469756	6.7745317	-5.5826575
C	-5.7652658	6.2426188	-4.2606018
C	-5.3739764	6.8518895	-3.0619614
C	-5.8342684	6.3696534	-1.8393397
C	-6.7011428	5.2719054	-1.7856282
C	-7.1028726	4.6692164	-2.9841056
C	-6.6337968	5.1472526	-4.2081141
C	-7.1491775	4.7060915	-0.4554611
N	-6.4279151	3.4753420	-0.1133546
O	-3.0527591	3.9860288	-0.0673648
C	-2.3224704	4.6543419	-0.8128580
C	-2.4883901	4.6175459	-2.3233708
C	-3.5287601	3.5817105	-2.7177289
C	-3.5573439	3.3355170	-4.2117934
O	-2.8406721	3.9685797	-5.0031659
N	-1.2889326	5.4309212	-0.3335448
C	-0.4217038	6.1786601	-1.2688738
C	-0.8932396	5.3723648	1.0301877
C	-1.8361632	5.2958805	2.0700339
C	-1.4112907	5.2152924	3.3889905
C	-0.0449969	5.2194500	3.6670578
C	0.9071181	5.3199452	2.6547356
C	0.4780307	5.3979806	1.3377232
N	0.4026473	5.1250369	5.0619271
N	-4.4074189	2.3372885	-4.6308362

C	-4.4780135	1.9517575	-5.9991535
C	-4.5429177	2.9117715	-7.0206135
C	-4.6428582	2.5131163	-8.3457907
C	-4.6773351	1.1507277	-8.6423723
C	-4.6171368	0.1798013	-7.6449492
C	-4.5165026	0.5862588	-6.3211164
N	-4.7862772	0.7274773	-10.0452648
C	-5.1813073	1.5445417	-3.6505291
H	1.0756760	10.0132561	-5.3093480
H	2.2339315	7.8567992	-4.8244038
H	-1.5300225	5.8170670	-4.9132990
H	-1.4030632	10.0503460	-5.5873314
H	-3.2859824	5.9719341	-5.5339673
H	-0.4001054	4.2754104	-5.2403921
H	0.7734127	2.2449153	-5.2901646
H	2.2128081	3.0572845	-4.6344569
H	-5.6166245	7.7876252	-5.7638451
H	-5.5818632	6.1385347	-6.4078192
H	-0.9624000	1.3665595	-3.7920136
H	-1.6695303	0.7564720	-1.5080062
H	1.6593339	2.9340657	0.1082879
H	2.3654149	3.5516382	-2.1859635
H	-4.7066003	7.7099540	-3.0892699
H	-5.5181527	6.8506452	-0.9164214
H	-7.7768344	3.8167212	-2.9528765
H	-6.9476807	4.6644404	-5.1310371
H	-0.1668682	2.2329980	1.5783807
H	-0.0367666	0.5118085	1.1831133
H	-8.2104156	4.4489097	-0.4795258
H	-6.9799287	5.4323283	0.3445867

H	-2.4968893	2.1319764	0.8963193
H	-5.4798281	3.5678367	0.2339784
H	-4.2359211	2.0651724	0.0637636
H	-6.5920125	-2.1640025	1.1331800
H	-7.8764527	-0.1812143	0.3308414
H	-4.1171804	-2.0046336	1.4062762
H	-1.5231861	4.3805751	-2.7845282
H	-4.5230136	3.8972114	-2.3852958
H	1.2166200	5.4553138	0.5471373
H	1.9628214	5.3219928	2.8972788
H	-2.1283861	5.1621697	4.1992297
H	-2.8935160	5.3089023	1.8490592
H	-4.4534280	-0.1626274	-5.5390103
H	-4.6388990	-0.8712606	-7.9058892
H	-4.7067062	3.2432861	-9.1434558
H	-4.5273954	3.9648422	-6.7780316
H	-1.0309415	6.6310639	-2.0501310
H	0.0701884	6.9796217	-0.7183011
H	0.3315683	5.5285322	-1.7258891
H	-5.9729001	1.0184626	-4.1817131
H	-4.5498137	0.8202243	-3.1259643
H	-5.6462467	2.2142146	-2.9266685
H	-3.3209879	2.6384741	-2.2015805
H	-2.7794821	5.6075379	-2.6923480
O	-0.4580709	4.9955028	5.9421820
O	1.6188966	5.1787263	5.2884141
O	-4.8340014	-0.4859645	-10.2853913
O	-4.8257070	1.6057428	-10.9165356

Table 26: Cartesian coordinates and total energy in a.u. of complex **2a@1**

E(RI-TPSS-D3/def2-TZVP)=

C	-5.1532638	-1.1035438	0.4702363
C	-4.2888563	-0.3486441	-0.3351533
C	-4.7780554	0.7548785	-1.0490747
C	-6.1185886	1.0997817	-0.9637044
C	-6.9611950	0.3346733	-0.1573739
C	-6.4970511	-0.7644403	0.5615990
N	-2.9202492	-0.7270646	-0.4442594
C	-1.9292899	0.2176026	-0.3087417
C	-0.5401831	-0.2773608	-0.4475398
C	0.4744740	0.3862111	0.1170047
C	1.8579613	-0.1420001	0.0275977
O	2.0769378	-1.3373972	-0.2380984
N	-8.3835476	0.6976987	-0.0661502
O	-9.1196793	-0.0077564	0.6348819
O	-2.1765596	1.4114345	-0.0587452
N	2.8653089	0.7543782	0.3034299
C	4.2113501	0.3207453	0.4615867
C	4.7896409	-0.6116932	-0.4136316
C	6.1094969	-1.0029996	-0.2337015
C	6.8414586	-0.4590329	0.8205762
C	6.2896240	0.4743871	1.6957636
C	4.9708905	0.8640518	1.5101057
N	8.2394121	-0.8729687	1.0089528
O	8.7055425	-1.7170677	0.2335403
O	8.8795997	-0.3549021	1.9328707
O	-8.7710567	1.6899042	-0.6955666

N	0.8693226	-3.2544750	-2.6322233
C	0.9728783	-2.5316669	-3.9039613
C	0.1910360	-1.2353953	-3.8822111
C	0.8450434	-0.0039508	-3.7658969
C	0.1197246	1.1835216	-3.7116697
C	-1.2789087	1.1661919	-3.7693471
C	-1.9342408	-0.0648254	-3.8751688
C	-1.2079676	-1.2545737	-3.9364742
C	-2.0570150	2.4666913	-3.7200132
N	-1.6798087	3.2783597	-2.5651538
C	-0.8716378	4.3687795	-2.6726460
O	-0.5292589	4.8392299	-3.7667809
C	0.0188917	-4.3021839	-2.4530669
O	-0.7246241	-4.7165095	-3.3541780
C	0.0466895	-4.9525461	-1.0999473
C	-0.4478108	-6.2575771	-0.9826874
C	-0.4228290	-6.9067734	0.2507640
C	0.0854460	-6.2592599	1.3755937
C	0.5764843	-4.9514127	1.2734135
C	0.5433087	-4.3037040	0.0347004
C	1.0830873	-4.2817865	2.5192113
N	1.8666575	-3.1838013	2.3360323
C	2.2511652	-2.3063726	3.4360660
C	1.3677572	-1.0741472	3.5198603
C	-0.0154880	-1.1698629	3.3319838
C	-0.8226672	-0.0371881	3.4158502
C	-0.2685015	1.2169569	3.6914918
C	1.1149099	1.3124547	3.8917470
C	1.9217406	0.1789051	3.8018692
C	-1.1333607	2.4598769	3.7169109

N	-1.0001605	3.2384264	2.4794748
C	-0.1062002	4.2560672	2.3576937
O	0.6168320	4.6183996	3.2979282
O	0.7966762	-4.7168614	3.6437766
C	-0.4149116	4.9900294	-1.3827422
C	-0.5024322	4.3298440	-0.1527944
C	-0.0424888	4.9355645	1.0204126
C	0.5255228	6.2142724	0.9549251
C	0.6134931	6.8791004	-0.2669898
C	0.1494369	6.2712188	-1.4323988
H	-0.7993448	-7.9219374	0.3348357
H	-0.8426293	-6.7487322	-1.8665048
H	0.8686275	-3.2699187	-0.0400539
H	0.1062445	-6.7507495	2.3432499
H	2.0308561	-2.8304433	1.3982883
H	1.4523287	-2.9339551	-1.8665401
H	2.0313100	-2.3364434	-4.0976415
H	0.5914334	-3.2016586	-4.6782212
H	2.1741994	-2.8966163	4.3540567
H	3.2973131	-2.0117668	3.3068741
H	1.9315778	0.0253351	-3.7213656
H	0.6418909	2.1330960	-3.6258693
H	-3.0208045	-0.0949034	-3.9196920
H	-1.7252143	-2.2062696	-4.0298412
H	-0.4652271	-2.1350739	3.1167254
H	-1.8962602	-0.1279012	3.2630343
H	1.5545969	2.2819801	4.1098919
H	2.9947722	0.2670809	3.9570155
H	-3.1328143	2.2671595	-3.6871182
H	-1.8532895	3.0736314	-4.6064338

H	-0.8439757	3.1170979	4.5400257
H	-2.1869883	2.1926984	3.8307178
H	-1.8784450	2.8853960	-1.6494209
H	-1.5670235	2.9554350	1.6874697
H	-0.8952899	3.3178483	-0.1109761
H	1.0465106	7.8739973	-0.3112709
H	0.8908936	6.6713660	1.8691531
H	0.2196818	6.7730815	-2.3921991
C	-2.5978863	-2.1671355	-0.5759188
H	-0.3397715	-1.1799922	-1.0130403
H	0.2729948	1.2817248	0.6937784
C	2.5754454	2.1888607	0.5371664
H	-4.7757505	-1.9461750	1.0399119
H	-7.1752981	-1.3324156	1.1866647
H	-6.5155758	1.9403844	-1.5197772
H	-4.1131326	1.3303214	-1.6788798
H	4.5279828	1.5811052	2.1924858
H	6.8817264	0.8768958	2.5085877
H	6.5751293	-1.7115893	-0.9079408
H	4.2157052	-1.0160176	-1.2356504
H	-3.5292876	-2.7166736	-0.6970078
H	-1.9949719	-2.3295173	-1.4723628
H	-2.0683045	-2.5396274	0.3041950
H	3.5000765	2.7481032	0.4014714
H	1.8576636	2.5414082	-0.2042883
H	2.1884564	2.3636476	1.5453923

Table 27: Cartesian coordinates and total energy in a.u. of complex **3e@1**

E(RI-TPSS-D3/def2-TZVP)=

C	-4.7312587	1.0705960	0.3112350
C	-4.0037757	-0.0593733	0.6976325
C	-4.6867875	-1.2480380	0.9860835
C	-6.0765764	-1.2972235	0.8946978
C	-6.7965273	-0.1645982	0.5169219
C	-6.1269653	1.0321960	0.2318753
C	-2.5032463	-0.0658721	0.7776494
O	-1.8683024	-1.1306240	0.7908267
C	-6.9541337	2.2326406	-0.1319488
O	-8.1395800	2.1168650	-0.4765636
N	-1.8928304	1.1495342	0.8345762
C	-0.4522587	1.2982250	0.6753555
C	-0.0619578	1.6690036	-0.7463968
C	-0.8014664	1.2178306	-1.8441881
C	-0.4358433	1.5717261	-3.1421380
C	0.6799601	2.3817752	-3.3744127
C	1.4371709	2.8130532	-2.2772194
C	1.0672565	2.4615892	-0.9806975
C	1.0174874	2.8571377	-4.7714992
N	0.4727623	4.1980491	-5.0173207
C	1.1845930	5.3264342	-4.7528473
O	2.3712751	5.2928363	-4.3928692
C	0.4665845	6.6321643	-4.9443752
C	-0.9264409	6.7171033	-5.0189704
C	-1.5638699	7.9474157	-5.2054830
C	-0.7886557	9.1105821	-5.2995216

C	0.6010605	9.0348534	-5.2151827
C	1.2294811	7.8033214	-5.0356648
C	-3.0587782	8.0797146	-5.2908414
O	-3.6102314	9.1876175	-5.2151805
N	-3.7586893	6.9246938	-5.4654746
C	-5.2183349	6.8772195	-5.4165100
C	-5.7148169	6.3173226	-4.0980567
C	-5.2866601	6.8888309	-2.8938053
C	-5.7256698	6.3811106	-1.6738138
C	-6.6073028	5.2952400	-1.6284398
C	-7.0430042	4.7282418	-2.8323531
C	-6.5956227	5.2314709	-4.0541967
C	-7.0343040	4.7035609	-0.3031239
N	-6.3412785	3.4429852	-0.0225826
O	-3.0566886	3.8618680	-0.0455137
C	-2.2985270	4.5434302	-0.7598575
C	-2.4274790	4.5439695	-2.2755155
C	-3.4816558	3.5457860	-2.7248534
C	-3.4934186	3.3515053	-4.2282067
O	-2.7748855	4.0246680	-4.9917197
N	-1.2860359	5.3007878	-0.2381143
C	-0.3680382	6.0481464	-1.1215697
C	-0.9671000	5.2405057	1.1592404
C	-1.9672971	5.3253836	2.1348613
C	-1.6241758	5.2549345	3.4842101
C	-0.2908597	5.1085638	3.8736107
C	0.7053367	5.0414645	2.8991131
C	0.3715488	5.1061788	1.5464231
H	-0.0310771	5.0535088	4.9266625
N	-4.3282067	2.3743230	-4.6907996

C	-4.3654778	2.0377373	-6.0858051
C	-4.5689611	3.0244311	-7.0546578
C	-4.6205968	2.6710897	-8.4020921
C	-4.4786711	1.3363477	-8.7895677
C	-4.2834707	0.3530116	-7.8187262
C	-4.2219411	0.7003572	-6.4687016
H	-4.5230798	1.0653318	-9.8402651
C	-5.1281281	1.5486240	-3.7632201
H	1.1970709	9.9395266	-5.2918069
H	2.3102410	7.7299296	-4.9666341
H	-1.5202214	5.8180021	-4.8924173
H	-1.2907722	10.0629330	-5.4373873
H	-3.2774756	6.0282550	-5.4499409
H	-0.5159700	4.2555028	-5.2418008
H	0.5959754	2.1839139	-5.5226730
H	2.0984967	2.9161224	-4.9144367
H	-5.5701251	7.9029612	-5.5581796
H	-5.5865463	6.2723803	-6.2508507
H	-1.0303471	1.2217654	-3.9836511
H	-1.6721401	0.5880297	-1.6861536
H	1.6615382	2.8084151	-0.1389449
H	2.3059340	3.4432801	-2.4460829
H	-4.6056132	7.7362337	-2.9147756
H	-5.3798703	6.8315862	-0.7461338
H	-7.7264155	3.8828516	-2.8086495
H	-6.9347200	4.7751345	-4.9816562
H	-0.0966864	2.0656295	1.3698181
H	0.0018026	0.3435784	0.9580713
H	-8.1028242	4.4776284	-0.3011075
H	-6.8204007	5.4021564	0.5114435

H	-2.4407268	2.0038647	0.7612445
H	-5.3547746	3.4978192	0.2123202
H	-4.2035880	1.9775074	0.0322563
H	-6.6014825	-2.2205362	1.1219277
H	-7.8791768	-0.1875714	0.4433286
H	-4.1127920	-2.1224278	1.2762153
H	-1.4569223	4.2933213	-2.7181096
H	-4.4749569	3.8700632	-2.3974278
H	1.1517124	5.0425545	0.7950753
H	1.7471646	4.9318097	3.1860800
H	-2.4066238	5.3215745	4.2349631
H	-3.0017516	5.4449685	1.8391056
H	-4.0579788	-0.0654795	-5.7161430
H	-4.1711338	-0.6877495	-8.1083656
H	-4.7799643	3.4423925	-9.1503563
H	-4.6871576	4.0580284	-6.7538314
H	-0.9354876	6.5698477	-1.8930960
H	0.1547669	6.7907245	-0.5198633
H	0.3619835	5.3844885	-1.5970584
H	-5.8820232	1.0197021	-4.3447612
H	-4.5077035	0.8202930	-3.2297305
H	-5.6366379	2.1881515	-3.0393181
H	-3.3022949	2.5817594	-2.2377868
H	-2.6813152	5.5502324	-2.6273947

Table 28: Cartesian coordinates and total energy in a.u. of complex **2e@1**

E(RI-TPSS-D3/def2-TZVP)=

C	-5.0898500	-0.4499762	-1.3286415
C	-4.2829866	-0.0538323	-0.2577042
C	-4.7697438	0.8482606	0.6930087
C	-6.0616110	1.3540862	0.5647418
C	-6.8714327	0.9639157	-0.5049568
C	-6.3829618	0.0588128	-1.4481342
N	-2.9586342	-0.5949059	-0.1366334
C	-1.8920389	0.2508285	-0.0089174
C	-0.5527602	-0.3755987	0.0407416
C	0.5516176	0.3763423	-0.0401479
C	1.8908541	-0.2502248	0.0093808
O	2.0331456	-1.4910311	-0.0440290
O	-2.0344569	1.4916128	0.0444125
N	2.9575824	0.5953235	0.1367693
C	4.2818804	0.0539682	0.2571209
C	4.7680179	-0.8478429	-0.6941741
C	6.0598217	-1.3540318	-0.5666749
C	6.8702121	-0.9643909	0.5027984
C	6.3823449	-0.0595443	1.4465345
C	5.0892460	0.4494859	1.3278842
N	1.0595011	-3.2572681	-2.4007899
C	1.2321033	-2.5011424	-3.6416724
C	0.3819075	-1.2482640	-3.6578693
C	0.9662897	0.0208277	-3.6071139
C	0.1758688	1.1687839	-3.6043378
C	-1.2196100	1.0716370	-3.6505814

C	-1.8043462	-0.1980216	-3.6869868
C	-1.0138995	-1.3465793	-3.6934747
C	-2.0751210	2.3243918	-3.6588939
N	-1.7358634	3.2150069	-2.5519898
C	-0.9449798	4.3117845	-2.7057916
O	-0.5958958	4.7357706	-3.8178587
C	0.3087354	-4.3894188	-2.3292585
O	-0.2345340	-4.8952260	-3.3233772
C	0.1966327	-5.0262503	-0.9720858
C	-0.3116243	-6.3282297	-0.8816640
C	-0.4040722	-6.9587049	0.3593229
C	0.0086375	-6.3016214	1.5180906
C	0.5189805	-4.9995217	1.4409509
C	0.5940242	-4.3709280	0.1959368
C	0.9466105	-4.3114531	2.7064199
N	1.7364063	-3.2139333	2.5521975
C	2.0757286	-2.3231309	3.6588847
C	1.2195581	-1.0708079	3.6510650
C	-0.1758438	-1.1685610	3.6042078
C	-0.9668171	-0.0209693	3.6070937
C	-0.3830342	1.2483701	3.6586060
C	1.0127183	1.3473060	3.6949694
C	1.8036886	0.1991166	3.6883057
C	-1.2337391	2.5009017	3.6420675
N	-1.0606576	3.2572299	2.4013400
C	-0.3084370	4.3884234	2.3300270
O	0.2354271	4.8934386	3.3242324
O	0.5982186	-4.7356428	3.8185933
C	-0.5170389	4.9992768	-1.4401337
C	-0.5933121	4.3706781	-0.1952065

C	-0.1953699	5.0253419	0.9729824
C	0.3146323	6.3266642	0.8828450
C	0.4081593	6.9571862	-0.3580379
C	-0.0050560	6.3007523	-1.5169887
H	7.8766534	-1.3614359	0.5972047
H	-7.8778640	1.3608390	-0.5999703
H	-0.7950306	-7.9700341	0.4223387
H	-0.6241302	-6.8320384	-1.7909254
H	0.9442576	-3.3462168	0.1406089
H	-0.0567986	-6.7825355	2.4891318
H	1.9459762	-2.8624212	1.6215777
H	1.4318917	-2.8264629	-1.5570255
H	2.2905986	-2.2422146	-3.7456579
H	0.9552229	-3.1709520	-4.4591631
H	1.9235066	-2.8913523	4.5807958
H	3.1350168	-2.0576950	3.5872989
H	2.0499468	0.1129874	-3.5731197
H	0.6456816	2.1487469	-3.5703141
H	-2.8877241	-0.2874096	-3.7186229
H	-1.4787723	-2.3290943	-3.7317741
H	-0.6452012	-2.1487237	3.5695355
H	-2.0504173	-0.1135975	3.5725361
H	1.4770785	2.3300235	3.7340111
H	2.8870179	0.2889965	3.7203372
H	-3.1346008	2.0596088	-3.5878260
H	-1.9219825	2.8924688	-4.5807364
H	-0.9577437	3.1707649	4.4598011
H	-2.2922082	2.2415757	3.7452337
H	-1.9462759	2.8636687	-1.6215004
H	-1.4327409	2.8264517	1.5574179

H	-0.9449139	3.3464525	-0.1400667
H	0.8003665	7.9680458	-0.4208154
H	0.6277060	6.8298642	1.7922511
H	0.0611056	6.7817633	-2.4879325
C	-2.7932625	-2.0585490	-0.2867362
H	-0.4505321	-1.4494743	0.1341178
H	0.4495008	1.4502098	-0.1336760
C	2.7925444	2.0589520	0.2873228
H	-4.7087128	-1.1472353	-2.0687413
H	-7.0041581	-0.2502755	-2.2837920
H	-6.4377207	2.0517112	1.3075053
H	-4.1407216	1.1468457	1.5237338
H	4.7084999	1.1463934	2.0685114
H	7.0040062	0.2491923	2.2819717
H	6.4353893	-2.0515918	-1.3097799
H	4.1385451	-1.1459462	-1.5247353
H	-3.7854171	-2.5040436	-0.3309167
H	-2.2472009	-2.3046746	-1.2016359
H	-2.2733387	-2.4732218	0.5804724
H	3.7848332	2.5041930	0.3309934
H	2.2722233	2.4739737	-0.5794792
H	2.2470968	2.3049858	1.2026078

Table 29: Cartesian coordinates and total energy in a.u. of complex **3h@1**

E(RI-TPSS-D3/def2-TZVP)=

C	-4.8624773	1.2100872	0.4056991

C	-4.4402222	-0.1215845	0.3644963
C	-5.3528144	-1.1382012	0.6731843
C	-6.6603910	-0.8173419	1.0369331
C	-7.0679362	0.5147180	1.0979958
C	-6.1656574	1.5402107	0.7878001
C	-3.0294598	-0.5088265	0.0202601
O	-2.7362859	-1.6765854	-0.2785467
C	-6.6427391	2.9635883	0.8583813
O	-7.8527844	3.2358273	0.8832966
N	-2.0991657	0.4807129	0.1009864
C	-0.6858533	0.2590621	-0.2184117
C	-0.3479447	0.7481288	-1.6105084
C	-0.7346746	0.0066003	-2.7339604
C	-0.4976153	0.4935678	-4.0184592
C	0.1347309	1.7262851	-4.2121222
C	0.5368081	2.4573543	-3.0889967
C	0.2963220	1.9744408	-1.8040282
C	0.3876116	2.2570731	-5.6122105
N	0.0897243	3.6812280	-5.7192896
C	1.0574578	4.6364770	-5.6706976
O	2.2674820	4.3639541	-5.6937579
C	0.5805888	6.0598230	-5.5979502
C	-0.7235034	6.3892651	-5.2184497
C	-1.1456525	7.7208786	-5.1748408
C	-0.2320229	8.7381013	-5.4784197
C	1.0764285	8.4179001	-5.8395970
C	1.4838826	7.0859263	-5.9031207
C	-2.5572339	8.1074273	-4.8331445
O	-2.8511948	9.2747069	-4.5332262
N	-3.4872468	7.1179206	-4.9174286

C	-4.9011175	7.3386840	-4.5996505
C	-5.2397392	6.8503766	-3.2074638
C	-4.8563004	7.5941524	-2.0843622
C	-5.0930423	7.1076846	-0.7996519
C	-5.7216540	5.8731542	-0.6053366
C	-6.1207754	5.1399584	-1.7281099
C	-5.8807321	5.6224435	-3.0133433
C	-5.9736374	5.3430007	0.7951645
N	-5.6751249	3.9191212	0.9030505
O	-2.7511692	3.5844029	0.0603631
C	-2.3181408	4.4978980	-0.6720836
C	-2.4326641	4.4300442	-2.1841378
C	-3.1525853	3.1694499	-2.6338345
C	-3.2687858	3.1019407	-4.1457351
O	-2.8351166	4.0147723	-4.8786282
N	-1.7383855	5.6190623	-0.1607832
C	-1.2654216	6.7042523	-1.0419817
C	-1.6976232	5.8337733	1.2596897
C	-1.1798797	4.8646654	2.1241350
C	-1.1574360	5.0853373	3.4959754
C	-1.6455890	6.2868281	4.0439246
C	-2.1458972	7.2635323	3.1647519
C	-2.1745742	7.0345331	1.7926937
N	-1.6917017	6.4752460	5.4245265
N	-3.8510997	1.9818127	-4.6564258
C	-3.8944628	1.7673540	-6.0768690
C	-4.4111710	2.7377823	-6.9404746
C	-4.4366681	2.5170416	-8.3122501
C	-3.9528071	1.3141685	-8.8609783
C	-3.4533608	0.3362760	-7.9826346

C	-3.4215409	0.5653381	-6.6106574
N	-3.9099437	1.1254106	-10.2416335
C	-4.3250160	0.8974990	-3.7746751
H	1.7796671	9.2101085	-6.0797927
H	2.4980000	6.8230377	-6.1877017
H	-1.4088913	5.6018522	-4.9224033
H	-0.5642787	9.7705698	-5.4332605
H	-3.2019341	6.1568722	-5.0889581
H	-0.8920229	3.9393028	-5.6417388
H	-0.2268529	1.7122668	-6.3358826
H	1.4389155	2.1298461	-5.8870756
H	-5.0821913	8.4115555	-4.6931096
H	-5.5036259	6.8122120	-5.3459162
H	-0.8089031	-0.0892210	-4.8818689
H	-1.2355846	-0.9483918	-2.5953406
H	0.6061752	2.5610496	-0.9417779
H	1.0382989	3.4126930	-3.2192230
H	-4.3579859	8.5504157	-2.2234264
H	-4.7843420	7.6922572	0.0634816
H	-6.6195308	4.1832420	-1.5974438
H	-6.1882350	5.0341330	-3.8752807
H	-0.0820520	0.7843267	0.5276850
H	-0.5056117	-0.8140536	-0.1260695
H	-7.0248779	5.4698870	1.0704984
H	-5.3591333	5.8886804	1.5181708
H	-2.3838370	1.4418824	0.2728345
H	-4.6934116	3.6611218	0.8245261
H	-4.1779622	1.9968413	0.1058521
H	-7.3628322	-1.6090473	1.2810781
H	-8.0813854	0.7781696	1.3844219

H	-5.0204304	-2.1707094	0.6299998
H	-1.4340909	4.4682715	-2.6320737
H	-4.1506651	3.1309349	-2.1848463
H	-2.5712397	7.8013478	1.1340366
H	-2.5227042	8.2033677	3.5605306
H	-0.7541409	4.3204477	4.1553121
H	-0.7963019	3.9340585	1.7219135
H	-3.0255874	-0.2024517	-5.9526951
H	-3.0797188	-0.6045744	-8.3790017
H	-4.8391793	3.2829053	-8.9709299
H	-4.7915392	3.6694498	-6.5376784
H	-0.6374577	6.2997165	-1.8394621
H	-2.1015118	7.2525055	-1.4877862
H	-0.6645214	7.3870838	-0.4429384
H	-4.9273743	0.2154194	-4.3730978
H	-3.4893527	0.3481875	-3.3293600
H	-4.9517617	1.3030707	-2.9767723
H	-2.6208076	2.2807926	-2.2755441
H	-2.9640357	5.3186220	-2.5432379
H	-1.0292693	5.9264498	5.9612391
H	-1.7165586	7.4411608	5.7317290
H	-3.8887935	0.1593689	-10.5487291
H	-4.5715724	1.6762883	-10.7771923

Table 30: Cartesian coordinates and total energy in a.u. of complex **2h@1**
E(RI-TPSS-D3/def2-TZVP)=

C	0.2080306	6.2922227	-0.8401249
C	-0.2995183	4.9913328	-0.9487280
C	-0.7299296	4.3330122	0.2056552
C	-0.6885537	4.9571665	1.4543992
C	-0.1793506	6.2586113	1.5496515
C	0.2671324	6.9184231	0.4050438
C	-0.3771822	4.3602852	-2.3112809
O	0.1615922	4.8913366	-3.2952061
C	-1.1495669	4.2654226	2.7064042
O	-0.8614071	4.7094508	3.8285119
N	-1.0915806	3.2064612	-2.4016324
C	-1.2292897	2.4622915	-3.6544116
C	-0.3389459	1.2381139	-3.6778593
C	1.0532029	1.3817740	-3.7019525
C	1.8805541	0.2597579	-3.6861733
C	1.3369298	-1.0280774	-3.6535738
C	-0.0550978	-1.1705681	-3.6261846
C	-0.8823890	-0.0492940	-3.6367739
C	2.2323151	-2.2524909	-3.6366356
N	1.8914245	-3.1480324	-2.5338574
C	1.1387365	-4.2688199	-2.7061922
O	0.8452140	-4.7124890	-3.8270703
C	0.6801191	-4.9585129	-1.4521779
C	0.7279590	-4.3338917	-0.2038898
C	0.2997103	-4.9903390	0.9523652
C	-0.2119074	-6.2898585	0.8462764
C	-0.2773539	-6.9165055	-0.3983468
C	0.1667341	-6.2584962	-1.5449271
C	0.3830712	-4.3583705	2.3141376

O	-0.1535885	-4.8876289	3.3002004
N	1.0995965	-3.2056162	2.4012704
C	1.2390474	-2.4586996	3.6522543
C	0.3447712	-1.2373389	3.6759867
C	-1.0468974	-1.3856694	3.7009612
C	-1.8780396	-0.2664932	3.6843480
C	-1.3388268	1.0231752	3.6500989
C	0.0526946	1.1703189	3.6223317
C	0.8838268	0.0518611	3.6336513
C	-2.2385577	2.2444391	3.6318655
N	-1.8979050	3.1422009	2.5308717
O	2.0759712	-1.4383788	0.0676698
C	1.8974751	-0.1992466	-0.0001595
N	2.9406567	0.6723657	-0.1273618
C	4.2840326	0.1756893	-0.2046124
C	4.7832054	-0.7033693	0.7622320
C	6.0907411	-1.1637281	0.6862405
C	6.9408263	-0.7571059	-0.3610532
C	6.4293272	0.1263436	-1.3293867
C	5.1185537	0.5820924	-1.2506858
N	8.2711485	-1.1616504	-0.3957754
C	0.5440621	0.3888426	0.0367485
C	-0.5426392	-0.3916012	-0.0402565
C	-1.8957148	0.1973489	-0.0030880
O	-2.0732171	1.4366000	-0.0715735
N	-2.9395895	-0.6732052	0.1253427
C	-4.2823441	-0.1749726	0.2037663
C	-5.1162605	-0.5799428	1.2508700
C	-6.4261975	-0.1220433	1.3311270
C	-6.9374595	0.7621063	0.3633129

C	-6.0880865	1.1670136	-0.6852179
C	-4.7813772	0.7046057	-0.7627038
N	-8.2669880	1.1690657	0.3997680
H	-0.6722389	-7.9255235	-0.4737005
H	0.1228139	-6.7366152	-2.5185728
H	1.0824513	-3.3118429	-0.1362831
H	-0.5486292	-6.7944461	1.7464229
H	1.4588801	-2.7483212	1.5644529
H	2.0473503	-2.7738072	-1.6005726
H	3.2804952	-1.9530974	-3.5386756
H	2.1216509	-2.8290319	-4.5592497
H	0.9825742	-3.1471344	4.4607089
H	2.2872103	-2.1630937	3.7636592
H	2.9607931	0.3843356	-3.7022069
H	1.4860455	2.3791098	-3.7331676
H	-1.9627426	-0.1764184	-3.6115946
H	-0.4932925	-2.1651747	-3.5953634
H	-1.4762903	-2.3844437	3.7332072
H	-2.9578385	-0.3946649	3.7007517
H	0.4875700	2.1663371	3.5904035
H	1.9637344	0.1826770	3.6078332
H	-2.2780334	2.1702361	-3.7699009
H	-0.9679304	3.1514805	-4.4606634
H	-2.1325390	2.8205139	4.5553290
H	-3.2853776	1.9412866	3.5308461
H	-1.4535081	2.7482852	-1.5664547
H	-2.0499696	2.7681284	1.5968445
H	-1.0810374	3.3099151	0.1362968
H	0.6588607	7.9285271	0.4823530
H	-0.1403911	6.7363727	2.5236889

H	0.5464385	6.7983137	-1.7387897
C	-2.7326653	-2.1302309	0.2852200
H	-0.4118846	-1.4634642	-0.1221309
H	0.4128917	1.4606410	0.1186802
C	2.7325699	2.1293701	-0.2860208
H	-4.7409047	-1.2530511	2.0162236
H	-7.0599284	-0.4468483	2.1525439
H	-6.4630383	1.8453486	-1.4476392
H	-4.1431758	1.0276979	-1.5774212
H	4.7430159	1.2547629	-2.0163432
H	7.0635925	0.4523458	-2.1499205
H	6.4658152	-1.8416379	1.4489771
H	4.1445378	-1.0276156	1.5761228
H	8.7223395	-1.1216232	-1.3025093
H	8.4855302	-2.0181997	0.1018542
H	-8.4804020	2.0260920	-0.0974621
H	-8.7170875	1.1297708	1.3070714
H	-2.2172500	-2.5423143	-0.5864247
H	-2.1639669	-2.3519475	1.1924062
H	-3.7118934	-2.6004052	0.3534704
H	3.7113902	2.6003811	-0.3542589
H	2.1634780	2.3513415	-1.1928927
H	2.2170666	2.5404108	0.5860783

Table 31: Cartesian coordinates and total energy in a.u. of complex **5@1**

E(RI-TPSS-D3/def2-TZVP)=

C	0.9376645	6.2330610	-1.9313735
C	0.4360462	4.9305369	-1.8179329
C	0.0869630	4.4406239	-0.5562806
C	0.2082249	5.2413602	0.5823330
C	0.7099198	6.5421182	0.4552618
C	1.0704820	7.0337013	-0.7979263
C	0.2877057	4.1184676	-3.0735538
O	0.8838020	4.4344472	-4.1153568
C	-0.1717321	4.7570441	1.9516969
O	0.2424983	5.3205020	2.9754902
N	-1.0086692	3.6860488	1.9907696
C	-1.4829188	3.0890341	3.2328895
C	-0.8817620	1.7216029	3.4825465
C	0.4938951	1.5141241	3.3350474
C	1.0491658	0.2600340	3.5770652
C	0.2448985	-0.8160922	3.9683358
C	-1.1308687	-0.6067427	4.1214161
C	-1.6873487	0.6489176	3.8785216
C	0.8366820	-2.1952928	4.1567022
N	0.5607203	-3.0593681	3.0064356
C	-0.2912066	-4.1169384	3.0726355
C	-0.4384750	-4.9297041	1.8173959
C	-0.0888495	-4.4400003	0.5558423
C	-0.2084315	-5.2412731	-0.5825704
C	-0.7091152	-6.5424028	-0.4553168
C	-1.0705316	-7.0336857	0.7977480
C	-0.9392806	-6.2325162	1.9310075
C	0.1727813	-4.7573685	-1.9517511
O	-0.2391682	-5.3221433	-2.9757544

O	-0.8896723	-4.4310615	4.1136231
N	1.0085743	-3.6855069	-1.9903114
C	1.4833203	-3.0881222	-3.2320563
C	0.8816338	-1.7209726	-3.4819080
C	1.6870663	-0.6478107	-3.8769312
C	1.1302478	0.6076368	-4.1200220
C	-0.2457829	0.8162537	-3.9683185
C	-1.0499054	-0.2603106	-3.5779820
C	-0.4942477	-1.5141690	-3.3355934
C	-0.8379732	2.1952268	-4.1570339
N	-0.5622245	3.0593728	-3.0067782
O	-1.7210300	1.7371365	-0.4986966
C	-1.8332698	0.6178111	0.0453230
C	-0.6383840	-0.2879454	0.2944488
C	0.6398407	0.2877522	-0.2950395
C	1.8345901	-0.6183151	-0.0462326
N	3.0484597	-0.1579882	-0.4347516
C	4.2512605	-0.9529168	-0.0960905
N	-3.0470324	0.1576006	0.4345190
C	-4.2500467	0.9520711	0.0954939
O	1.7219267	-1.7380553	0.4969497
H	-1.4566602	-8.0446183	0.8918743
H	-0.8119387	-7.1512055	-1.3479543
H	0.2459988	-3.4120384	0.4559739
H	-1.2183535	-6.6008512	2.9132104
H	0.9851837	-2.7870503	2.1217630
H	1.3464993	-3.2743251	-1.1249658
H	2.5739790	-3.0170044	-3.1956628
H	1.2100395	-3.7796736	-4.0347170
H	0.4089174	-2.6918488	5.0300294

H	1.9218983	-2.1267221	4.2871890
H	2.7590664	-0.7959605	-3.9857824
H	1.7672857	1.4371971	-4.4177916
H	-2.1202990	-0.1087320	-3.4578091
H	-1.1280415	-2.3375602	-3.0187552
H	-1.7679370	-1.4358388	4.4204650
H	-2.7591730	0.7975373	3.9884322
H	1.1277849	2.3371404	3.0174253
H	2.1193513	0.1078790	3.4557881
H	-1.9231561	2.1263115	-4.2876865
H	-0.4102202	2.6917735	-5.0303746
H	-1.2086380	3.7805213	4.0352799
H	-2.5736349	3.0185834	3.1972144
H	-0.9848081	2.7853968	-2.1216953
H	-1.3497285	3.2768498	1.1257606
H	-0.2489784	3.4130187	-0.4564800
H	1.4572779	8.0443912	-0.8919632
H	0.8143039	7.1503311	1.3481230
H	1.2161946	6.6015715	-2.9136638
C	-3.2330936	-1.1244478	1.1197535
C	3.2347177	1.1244688	-1.1193187
C	3.4373304	2.3186412	-0.1993021
H	4.0995763	1.0161699	-1.7811304
H	2.3788454	1.3029804	-1.7749912
C	-3.4366278	-2.3191130	0.2005439
H	-2.3765181	-1.3027185	1.7746729
H	-4.0975136	-1.0154929	1.7820524
C	4.5367353	-2.0703880	-1.0799370
H	5.0872214	-0.2488656	-0.0591196
H	4.1027369	-1.3684146	0.9033251

C	-4.5356006	2.0700525	1.0787357
H	-4.1017187	1.3669741	-0.9041929
H	-5.0858891	0.2478533	0.0590493
C	-4.1955341	3.3907147	0.7634215
C	-4.4579717	4.4268998	1.6609272
C	-5.0624771	4.1525147	2.8890333
C	-5.4087778	2.8379325	3.2122054
C	-5.1509415	1.8053720	2.3098028
H	-3.7153581	3.6003993	-0.1883582
H	-4.1821702	5.4458357	1.4046203
H	-5.2629023	4.9571582	3.5908773
H	-5.8844245	2.6178319	4.1638782
H	-5.4420192	0.7883948	2.5622974
C	5.1519998	-1.8051126	-2.3109188
C	5.4095487	-2.8371939	-3.2139415
C	5.0631369	-4.1519224	-2.8914411
C	4.4587717	-4.4269064	-1.6634055
C	4.1965402	-3.3911747	-0.7653102
H	5.4432297	-0.7880415	-2.5628518
H	5.8850743	-2.6166485	-4.1655761
H	5.2633704	-4.9562231	-3.5937345
H	4.1828037	-5.4459355	-1.4076454
H	3.7162571	-3.6013035	0.1863128
C	3.5802599	3.5894701	-0.7714326
C	3.7504055	4.7124012	0.0343684
C	3.7739421	4.5828531	1.4250129
C	3.6371141	3.3212925	2.0006270
C	3.4720802	2.1944886	1.1905181
H	3.5396154	3.7033463	-1.8526414
H	3.8400266	5.6928933	-0.4226631

H	3.8825262	5.4613565	2.0537239
H	3.6508339	3.2108027	3.0814716
H	3.3607269	1.2146781	1.6460963
C	-3.4705032	-2.1960265	-1.1893654
C	-3.6363800	-3.3233295	-1.9986233
C	-3.7749162	-4.5842607	-1.4220427
C	-3.7522169	-4.7127351	-0.0312844
C	-3.5812845	-3.5893083	0.7736331
H	-3.3577751	-1.2167324	-1.6457001
H	-3.6494446	-3.2136596	-3.0795542
H	-3.8843154	-5.4631405	-2.0500901
H	-3.8431387	-5.6927360	0.4265370
H	-3.5414600	-3.7025462	1.8549805
H	-0.5193953	-0.4352487	1.3739689
H	0.8448618	1.2761514	0.1318525
H	-0.8433404	-1.2763987	-0.1323547
H	0.5207636	0.4352402	-1.3745347

Table 32: Cartesian coordinates and total energy in a.u. of complex **4@1**
E(RI-TPSS-D3/def2-TZVP)=

C	0.6100719	6.0440195	-0.7324363
C	0.0043800	5.1813265	-1.6503815
C	-0.0613066	3.8140276	-1.3622546
C	0.4796760	3.3142777	-0.1794644
C	1.0889761	4.1805026	0.7311124
C	1.1523027	5.5462984	0.4539107

C	-0.5275408	5.7217443	-2.9628383
N	-1.8785494	5.2348132	-3.2851823
C	-2.9885313	5.8112433	-2.4895979
C	-3.7093597	6.9382995	-3.2020669
C	-4.9298721	6.7009133	-3.8461185
C	-5.6016249	7.7352999	-4.4991075
C	-5.0566066	9.0207875	-4.5203620
C	-3.8414758	9.2685891	-3.8772711
C	-3.1764991	8.2342164	-3.2172964
C	-2.1871892	4.2784864	-4.1988831
C	-1.0774010	3.5747212	-4.8839909
C	-1.3229339	2.5097589	-5.6566873
C	-0.2131462	1.8059178	-6.3417409
O	0.9840578	2.0993336	-6.1143048
O	-3.3843916	3.9846032	-4.4256973
N	-0.5217475	0.8500475	-7.2559562
C	-1.8728028	0.3637107	-7.5793217
C	-2.4026172	0.9036399	-8.8928078
C	-3.0028678	0.0401540	-9.8135573
C	-3.5425170	0.5375438	-11.0012480
C	-3.4820819	1.9037744	-11.2768845
C	-2.8782624	2.7708426	-10.3634305
C	-2.3397805	2.2714057	-9.1793910
C	0.5883707	0.2735306	-8.0512912
C	1.3083074	-0.8542873	-7.3391437
C	0.7746789	-2.1499009	-7.3247115
C	1.4388292	-3.1849665	-6.6649997
C	2.6539181	-2.9381871	-6.0214255
C	3.1997218	-1.6530184	-6.0419274
C	2.5287705	-0.6179211	-6.6946185

N	1.6576596	0.2124886	-3.3831303
C	0.7909352	-0.9652066	-3.3738426
C	-0.6757508	-0.5832672	-3.3944461
C	-1.5441483	-1.1076672	-4.3561604
C	-2.8861656	-0.7290273	-4.3830307
C	-3.3829356	0.1965792	-3.4580839
C	-2.5158448	0.7127805	-2.4897761
C	-1.1805146	0.3201564	-2.4529841
C	-4.8197203	0.6643544	-3.5461163
N	-5.0436882	1.4917631	-4.7346018
C	-5.6558585	1.0106983	-5.8496530
O	-6.0967896	-0.1478283	-5.9176340
C	-5.8074168	1.9638062	-7.0030445
C	-6.6427095	1.5808210	-8.0605700
C	-6.8316815	2.4294178	-9.1495299
C	-6.1821593	3.6605579	-9.2004661
C	-5.3390170	4.0548227	-8.1545578
C	-5.1549798	3.1995263	-7.0640606
C	-4.6105693	5.3602412	-8.2899766
N	-4.0577286	5.8718733	-7.1579812
C	-3.1914397	7.0499117	-7.1671171
C	-1.7246076	6.6684434	-7.1463843
C	-1.2196598	5.7644808	-8.0872395
C	0.1156672	5.3719279	-8.0500185
C	0.9826398	5.8888279	-7.0819718
C	0.4857737	6.8151838	-6.1578375
C	-0.8562891	7.1936657	-6.1850461
C	2.4193702	5.4210167	-6.9934540
N	2.6431200	4.5933495	-5.8051022
C	3.2556360	5.0739257	-4.6900266

O	3.6957692	6.2327091	-4.6212935
O	-4.5192409	5.9308070	-9.3885273
C	3.4089293	4.1197228	-3.5377903
C	2.7552263	2.8847076	-3.4762720
C	2.9413168	2.0280645	-2.3871799
C	3.7878193	2.4202347	-1.3432095
C	4.4385876	3.6507060	-1.3946368
C	4.2475010	4.5007095	-2.4821365
C	2.2119836	0.7231844	-2.2514159
O	2.1214783	0.1521415	-1.1530594
H	5.0908061	3.9527037	-0.5803960
H	3.9104864	1.7559075	-0.4939583
H	2.0601243	2.6050081	-4.2620160
H	4.7400641	5.4665146	-2.5319891
H	2.2141137	3.6688150	-5.8155834
H	1.7213951	0.7254111	-4.2563024
H	1.0355695	-1.5831066	-4.2411014
H	1.0332223	-1.5263848	-2.4669504
H	3.1037122	6.2693619	-6.9207718
H	2.6815796	4.8309155	-7.8768321
H	-1.1634176	-1.8112163	-5.0931287
H	-3.5555214	-1.1417279	-5.1342728
H	-2.8890179	1.4304460	-1.7622922
H	-0.5168695	0.7180877	-1.6908067
H	1.1550751	7.2285485	-5.4069109
H	-1.2371404	7.8976489	-5.4485551
H	-1.8831344	5.3660660	-8.8492981
H	0.4889093	4.6537393	-8.7769527
H	-5.0816462	1.2546311	-2.6627738
H	-5.5040312	-0.1840481	-3.6184202

H	-3.4339470	7.6111315	-8.0739231
H	-3.4363959	7.6675975	-6.2998033
H	-4.6134301	2.4157431	-4.7247646
H	-4.1218726	5.3593180	-6.2846457
H	-4.4623662	3.4808098	-6.2767120
H	-7.4812190	2.1258230	-9.9653153
H	-6.3030303	4.3237578	-10.0508491
H	-7.1343636	0.6145416	-8.0110486
H	-1.8297792	-0.7288741	-7.6131328
H	-2.5459957	0.6118871	-6.7585205
H	0.1449117	5.4747304	-3.7846183
H	-0.5712526	6.8142757	-2.9282147
H	0.1435932	-0.0792325	-8.9856853
H	1.2868538	1.0810926	-8.2780406
H	-3.6865004	5.0034636	-2.2620309
H	-2.5434737	6.1648002	-1.5556395
H	-5.3465787	5.6978984	-3.8354822
H	-6.5459571	7.5354860	-4.9974559
H	-5.5759344	9.8253112	-5.0332617
H	-3.4152696	10.2679656	-3.8827954
H	-2.2440050	8.4432137	-2.6982410
H	-0.1577645	-2.3581118	-7.8441728
H	1.0120602	-4.1841082	-6.6600612
H	3.1726199	-3.7432639	-5.5087636
H	4.1439984	-1.4539918	-5.5431599
H	2.9460295	0.3848756	-6.7046107
H	-3.0536124	-1.0248194	-9.5976600
H	-4.0091447	-0.1422087	-11.7088086
H	-3.9075026	2.2945910	-12.1968081
H	-2.8504901	3.8373987	-10.5659606

H	-1.8754542	2.9497524	-8.4677691
H	-0.5298296	3.1362286	-2.0716356
H	0.4494979	2.2480365	0.0243440
H	1.5163321	3.7893766	1.6500046
H	1.6232094	6.2254202	1.1592462
H	0.6630528	7.1086434	-0.9495008
H	-2.3477111	2.1828204	-5.7801191
H	-0.0526329	3.9016904	-4.7605104
